# Closed‐Loop Bioelectronic Artificial Pancreas Patch for Continuous Monitoring and Regulation of Blood Glucose in Diabetic Rats and Pigs

**DOI:** 10.1002/advs.202503536

**Published:** 2025-09-16

**Authors:** Yiqun Liu, Changxi Zhang, Lingyi Xu, Yuan Ma, Yuanyuan Ma, Ying Chen, Difei Lu, Le Ye, Li Yang, Yue Cui

**Affiliations:** ^1^ School of Materials Science and Engineering Peking University Beijing 100871 P. R. China; ^2^ Renal Division Peking University First Hospital Peking University Institute of Nephrology Key Laboratory of Renal Disease Ministry of Health of China Key Laboratory of Chronic Kidney Disease Prevention and Treatment (Peking University) Ministry of Education Beijing 100034 P. R. China; ^3^ Laboratory Animal Center Peking University First Hospital Beijing 100034 P. R. China; ^4^ Department of Endocrinology Peking University First Hospital Beijing 100034 P. R. China; ^5^ School of Integrated Circuits Peking University Beijing 100871 P. R. China

**Keywords:** artificial pancreas, closed‐loop, diabetes, glucose sensor, insulin pump

## Abstract

Integrated artificial pancreas devices with continuous glucose monitoring (CGM) and closed‐loop insulin delivery are crucial for diabetes management. However, design challenges remain, including miniaturization, low cost, stability, and low power consumption. Current commercial products are expensive ($3,000 to $8,000 USD), and bulky, typically exceeding 100 cm^3^. Here, a closed‐loop bioelectronic artificial pancreas patch is presented for continuous blood glucose regulation in diabetic rats and pigs. By designing transiently dissolvable microneedle arrays incorporated with microtube arrays, insulin is efficiently delivered into interstitial fluid, eliminating the need for an external long needle. Leveraging multi‐layer sensing electrodes, Ag/Ag_2_O glass‐fiber pumping electrodes, and a polyethylene‐glycol (PEG) functionalized polycarbonate membrane, the closed‐loop artificial pancreas patch demonstrates highly stable sensing and pumping with low power consumption (only 0.422 mW), enabling long‐term continuous operation to regulate blood glucose. In particular, diabetic pigs are operated on for 3 consecutive days, showing steady blood glucose control with a time in range (3.9–10.0 mm) of ≈67.98%, comparable to a commercial closed‐loop system at ≈74.95%. The total volume of the entire system is ≈2 cm^3^, and the cost is ≈$10. This method opens up promising avenues for the development and application of wearable devices to manage diabetes.

## Graphical abstract

A closed‐loop bioelectronic artificial pancreas patch is presented for glucose monitoring and regulation. Utilizing transiently dissolvable microneedle arrays integrated with microtube arrays, the patch efficiently delivers insulin into interstitial fluid, eliminating the need for external long needles. In diabetic pigs, it demonstrates excellent blood glucose control comparable to commercial closed‐loop systems, while offering advantages such as miniaturization, low cost, and low power consumption.

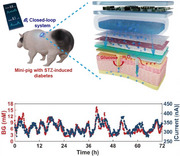

## 1 Introduction

Diabetes mellitus^[^ ^1^ ^]^ is a chronic metabolic disease caused by insulin deficiency (type 1 DM) or insulin resistance (type 2 DM) that manifests as hyperglycemia.^[^ ^2^ ^]^ Diabetes can result in complications such as heart attack, diabetic kidney disease, stroke, high blood pressure, diabetic retinopathy, and neuropathy.^[^ ^3^ ^]^ Globally, more than 415 million people suffer from diabetes and its complications.^[^ ^4^ ^]^ According to the IDF diabetes Map 2021, diabetes has led to at least US $966 billion in medical expenditure, an increase of 316% in the past 15 years.^[^ ^5^ ^]^ Existing therapeutic approaches for diabetes management, encompassing both multiple daily insulin injections and continuous subcutaneous insulin infusion, necessitate patient self‐administration. This regimen making treatment decisions multiple times a day can be challenging for many patients.^[^ ^6^ ^]^ As a result, these methods make it difficult to maintain the blood glucose levels of a patient within the normal range and avoid hypoglycemia and hyperglycemia throughout the day.^[^ ^7^ ^]^

Artificial pancreas diabetes management systems, also known as “artificial pancreas systems”, are the most promising methods of blood glucose regulation to help patients overcome these problems.^[^ ^8^ ^]^ The main components of an artificial pancreas system include an infusion pump for delivering insulin to lower blood glucose levels, a continuous glucose monitoring (CGM) device for measuring blood glucose levels, and a control algorithm for determining when and how much insulin is needed.^[^ ^8^, ^9^ ^]^ For type 1 diabetes patients, the closed‐loop artificial pancreas devices can greatly improve the quality of life of patients.^[^ ^10^ ^]^ To date, there are only a few commercially available artificial pancreas devices on the market. These devices (e.g., the MiniMed 770G/780G system developed by Medtronic) are expensive, ranging from ≈$3000–$8000 USD.^[^ ^11^ ^]^ The high price of commercial systems and their consumables is the major barrier limiting the widespread use of closed‐loop systems in the general diabetic population. They are also uncompact and bulky, with typical dimensions of ≈5 cm × 9 cm × 2.5 cm and a total volume usually in excess of 100 cm^3^.^[^ ^12^ ^]^ These devices are painful and uncomfortable to wear as well.^[^ ^13^ ^]^

Over the past decade, significant advances have been made in digital health and wearable devices for sensing interstitial biomarkers^[^ ^14^ ^]^ and drug delivery.^[^ ^15^ ^]^ More recently, several attempts have been made to develop wearable microneedle‐based devices for closed‐loop management of diabetes, such as sweat glucose biosensors on flexible substrates with thermoresponsive drug‐loaded microneedles,^[^ ^16^ ^]^ an iontophoresis glucose sensor with mesoporous microneedles,^[^ ^14c^ ^]^ a glucose sensor with a microneedle puncher and peristaltic pump,^[^ ^17^ ^]^ and glucose‐responsive microneedles.^[^ ^18^ ^]^ We have also previously developed a thermoplastic microneedle‐based sensor with an electrochemical pump,^[^ ^19^ ^]^ biodegradable microneedle sensors with an electroosmotic micropump^[^ ^20^ ^]^ and an ultrasonic pump.^[^ ^21^ ^]^ These closed‐loop devices have excellent wearability owing to these advances. However, the sharp tips of the microneedles result in limited insulin delivery efficiency, and breakage of the microneedle within the skin can lead to tissue damage.^[^ ^22^ ^]^ To overcome these limitations, we developed a microtube‐based closed‐loop system for improving insulin delivery and absorption.^[^ ^23^ ^]^ However, an external stainless‐steel needle with a mechanical spring of ≈5 mm in length is always required to insert the microtubes into the skin, which not only significantly increases the size of the overall device but also causes skin pain.^[^ ^24^ ^]^

Despite the tremendous promising prospects offered by these technologies, significant challenges remain to fully realize their potential for practical applications in diabetes patients: human applications require miniaturizing the entire artificial pancreas system, including both the glucose sensing and insulin pumping devices, and the miniaturization further requires a low power consumption to reduce the battery size and a wireless and miniaturized design of a printed circuit board (PCB) that controls the sensor and pump; a safe, long‐term, and continuous closed‐loop management of diabetes is critical for real applications, which further requires highly stable and precise performances of the glucose sensor and insulin pump; moreover, the successful demonstration of the closed‐loop device in large animals with body weights and physiological metabolisms similar to those of humans is necessary for further implementation in human diabetics. All these challenges result in current wearable closed‐loop patches that are still far from practical applicability.

Therefore, to address these challenges, we demonstrate for the first time an integrated wearable closed‐loop bioelectronic artificial pancreas patch with transiently dissolvable microneedles for ultra‐low‐cost, low‐power and highly stable closed‐loop diabetes management in diabetic rats and pigs. Dissolvable microneedles were fabricated and further integrated with 3D‐printed microtubes to avoid the low efficacy of insulin delivery by microneedles and exclude the use of external long needles. The microneedles assisted the insertion of the microtubes into the skin. The microneedles were only gently pierced into the skin; the microneedles then dissolved, and the microtubes were then left in the dermis. Sensing electrodes were constructed on the sidewalls of the microtubes with a multi‐layer structure to continuously detect interstitial glucose in dermis with high stability. The pump's energy consumption is several orders of magnitude higher than that of the sensor, and a non‐gassing electroosmotic micropump was constructed to minimize its power consumption, reducing the power consumption of the entire system. The biosensor and micropump were powered and controlled by a PCB that enabled wireless communication with a smartphone via Bluetooth. With a diameter of 1.5 cm and a total thickness of ≈1 cm, the system is compact, convenient, easy to wear, and fairly inexpensive to manufacture at ≈$10 USD. The in‐vivo performance of the closed‐loop artificial pancreas patch was evaluated in diabetic rats and pigs. Both animals showed partial similarities to humans. The skin of diabetic rats is thinner (≈0.2–1 mm^[^ ^25^ ^]^) and more similar to that of humans (≈0.5–2 mm^[^ ^26^ ^]^) than that of pigs. However, the body weight and physiological metabolism of rats are quite different from those of humans. Pigs have much thicker skin than humans (≈2–7 mm^[^ ^27^ ^]^) and have body weight and physiological metabolism very similar to those of humans. Therefore, both diabetic rats and pigs were studied to evaluate the artificial pancreas patch.

## 2 Results

### 2.1 Overall Working Scheme of the System

The most important requirements in designing a wearable patch for long‐term and continuous management of diabetes are miniaturization and integration of the entire device to wear it on the body. Each device component should be small to achieve miniaturization, including the sensor, pump, electronics, and wireless data transmission component, which must be physically integrated into a coin‐scale size.

**Figure 1:**
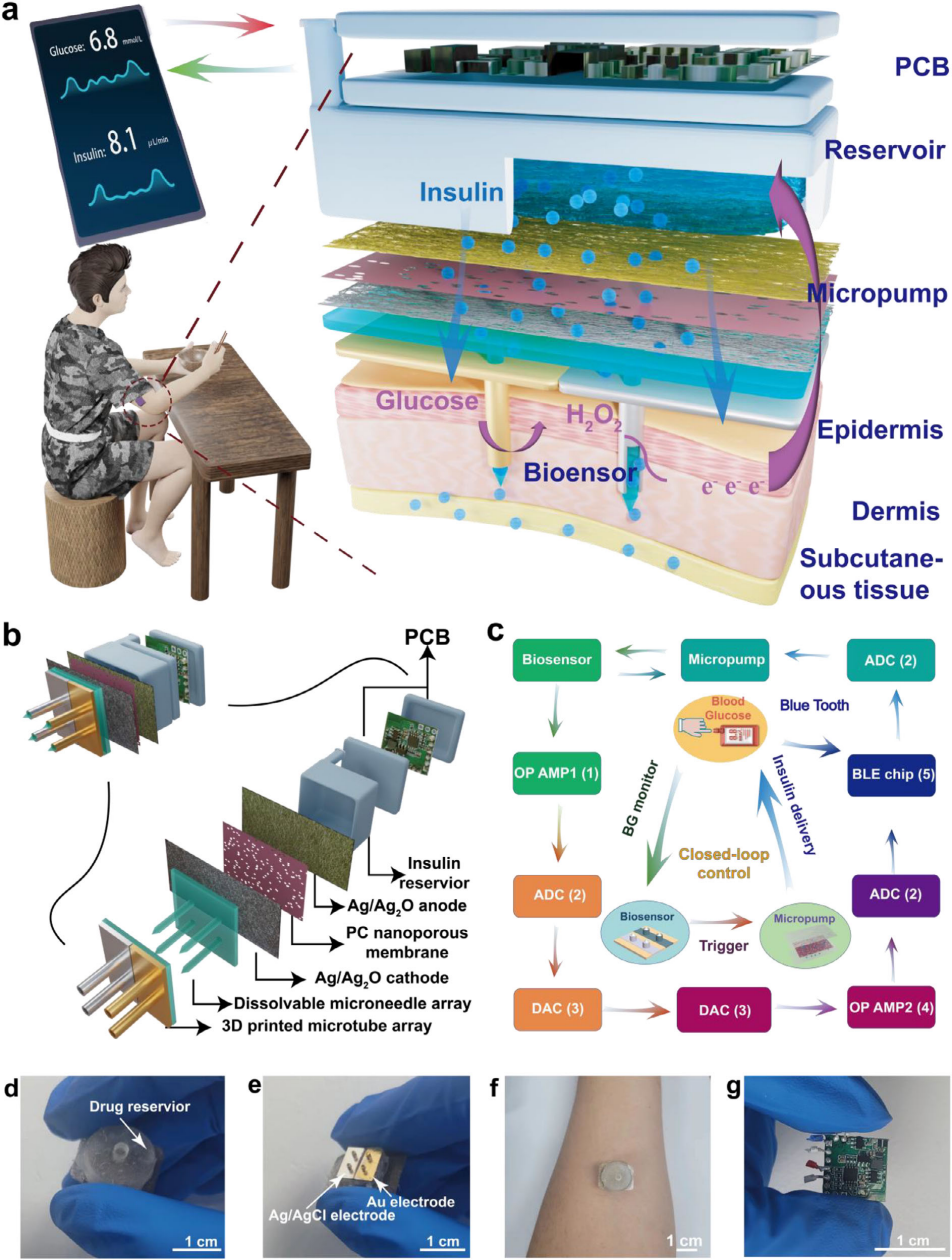
General schemes and images of the closed‐loop system. a) Schematic of the overall principle of the closed‐loop system on human skin. b) Illustration of the whole structure of the system (insulin reservoir is blue, Ag/Ag_2_O anode is yellow, PC nanoporous membrane is purple, Ag/Ag_2_O cathode is gray, dissolvable microneedle array is green, 3d printed microtube array is gold and silver). c) An illustration of the closed‐loop signal and instruction transmit route. (1: OP AMP1 (operational amplifier 1), 2: ADC (analog to digital converter), 3: DAC (digital to analog converter), 4: OP AMP2 (operational amplifier 2), 5: BLE chip (Bluetooth chip)). d) A photograph of this overall system. e) A photograph of the microtube biosensor (the dimensions of a microtube were 23 mm in height, 15 mm in outer diameter, and 10 mm in inner diameter. The dimensions of a dissolvable microneedle were 0.9 mm in diameter, 2.5 mm in cylinder height, and 0.5 mm in top cone height). f) A photograph of the system applied to the human arm. g) Front photograph of the printed circuit board (PCB).

**Figure** **1**a depicts the overall working principle and the sensing and pumping components on a user's skin for diabetes management. Transiently dissolvable microneedles are integrated into 3D‐printed microtubes. The microneedles were manufactured inside the hollow microtubes, and shaped parts appeared on top of the microtubes. The microneedles were inserted into the epidermis and dermis skin layers to bring the microtubes inside the skin. They were then dissolved by interstitial fluid and insulin solution, leaving the microtubes inside the skin. The sensing elements were fabricated on the sidewalls of the microtubes to form working and reference/counter electrodes for achieving CGM in the interstitial fluid of the epidermis and dermis. The working electrode is usually composed of an Au composite material, and the reference/counter electrode is usually composed of an Ag/AgCl material. A low‐power electroosmotic micropump was designed with Ag/Ag_2_O glass fiber membranes as the anode and cathode and operated under low potential (<1 V) without generating gases. The micropump was placed above the hollow microtubes, and a 3D‐printed insulin reservoir was integrated with the micropump to form the complete closed‐loop artificial pancreas system. Interstitial glucose molecules were catalyzed by glucose oxidase (GOD) immobilized on the Au/Prussian blue (PB) electrode on the sidewalls of the microtube to generate H_2_O_2_, which was further reduced to generate a current signal. The PCB further automatically processed the sensing signal according to the control algorithm to direct the electroosmotic micropump to inject an appropriate amount of insulin into the hollow microtubes and, consequently, into the interstitial fluid of the dermis and epidermis to lower the blood glucose.

Figure 1b illustrates the structure of the system. The main components of the system from top to bottom are a miniature wireless PCB (green), an insulin reservoir (blue), an electroosmotic micropump with Ag/Ag_2_O glass‐fiber electrodes (yellow and gray), a nanoporous polycarbonate (PC) membrane (purple), a dissolvable microneedle array (green), and 3D‐printed microtubes (gold and silver). The PCB powers the biosensor, monitors amperometric current, regulates insulin release via a micropump, and transmits information wirelessly via Bluetooth. Figure 1c shows the transmission routes for signals and commands in the artificial pancreas system. The glucose sensing signal from the microtube biosensor is transmitted through two operational amplifiers (OP AMP1 and OP AMP2), an analog‐to‐digital converter (ADC), and a digital‐to‐analog converter (DAC), and then arrives at the Bluetooth chip (BLE chip) which determines whether the current value exceeds the critical value. If the threshold is exceeded, the PCB powers the electroosmotic micropump to continuously deliver insulin.

Figure 1d shows a photograph of the artificial pancreas system. The system has a total diameter of only 1.5 cm and total thickness of ≈1 cm, which includes the sensor (≈5 mm thick), micropump (≈1 mm thick), and insulin reservoir (≈5 mm thick). The insulin reservoir could hold ≈0.88 ml insulin, which could be replaced with a larger volume to accommodate more insulin and eliminate the need for frequent refills. Figure 1e shows a camera image of the microtube biosensor with an Au working electrode (yellow) and an Ag/AgCl counter/reference electrode (gray) deposited. Owing to the high wearability and compactness of the artificial pancreas system, a tight and stable interaction is formed between the system and skin (Figure 1f). Figure 1g and Figure S1 (Supporting Information) show a camera image of the miniature wireless PCB and its front and back (1 × 1 cm, ≈3 mm thick). Two OP AMPs, a DAC, an ADC, and a BLE chip are the main components of this PCB. Figure S2 (Supporting Information) shows the photograph of the entire closed‐loop system, which meets the size requirements for miniaturization.

The entire system is cost‐effective for mass production, and the manufacturing cost is ≈$10 USD, as shown in the section of Manufacturing cost analysis of the entire system in Supporting Information. In comparison, the prices of commercial closed‐loop systems range from $3000–$8000 USD.^[^ ^11^ ^]^ According to annual reports from Medtronic, Dexcom, and Tandem Diabetes Care,^[^ ^28^ ^]^ which indicate a gross profit margin of 50%–65% and a cost of sales of 35%–50%, and considering a typical manufacturing cost proportion of ≈40% for a medical device manufacturing company,^[^ ^29^ ^]^ the manufacturing cost of the commercial closed‐loop system is estimated to be ≈$1000–$4000 USD. Therefore, our system provides significant advantages over commercial systems in both size and cost, which can facilitate its widespread adoption among the general diabetic population. The low manufacturing cost of our system is attributed to its simple structure, integration of glucose measurement and insulin injection into a single device, elimination of the need for steel needles and attached mechanical components for skin insertion, and insulin administration using a thin film pump.

### 2.2 Fabricating a Stable and Partially Transiently Dissolvable Sensor

Since glucose sensing and insulin delivery are performed within one integrated device, they will interact with each other. Therefore, designing the sensing device configuration must consider the insulin delivery process. The sensing structure is designed with transiently dissolvable microneedle arrays in combination with 3D microtube arrays to achieve efficient insulin delivery with simple skin piercing. The microneedle arrays are used to puncture the skin and guide the microtube to be inserted into the skin, followed by full dissolution by interstitial fluid in the skin as well as insulin solution released by the pump, leaving only the microtube arrays inside the skin for sensing and drug delivery. The outer layer of the microtube acts as the sensor, and the hollow inner layer is for drug delivery. In terms of insulin delivery, the microtube can be more effective than the microneedle; the tips of the hollow microneedles could be easily blocked by bubbles, preventing liquid release (Figure S3, Supporting Information).

**Figure 2:**
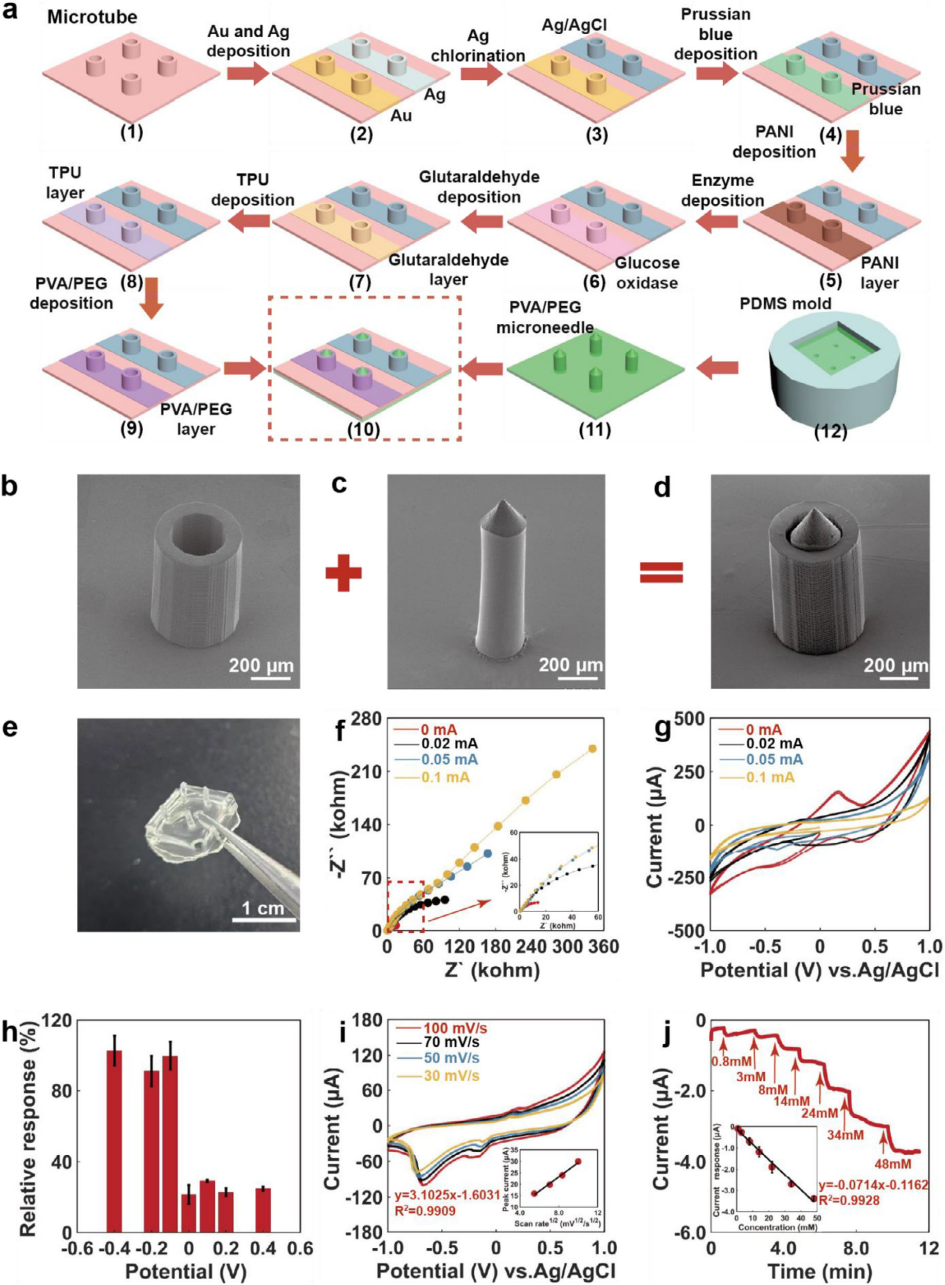
Fabrication process and characterization of the sensing electrode. a) Fabrication procedures of the sensor. b) SEM image of the microtube (height: 1 mm; outside diameter: 0.6 mm; inner diameter: 0.4 mm). c) SEM image of the dissolvable microneedle (diameter: 0.35 mm; cylinder height: 1.2 mm; cone height: 0.2 mm). d) SEM image of the integration of the microtube and dissolvable microneedle. e) A photograph of the dissolvable microneedle array (diameter: 0.9 mm; cylinder height: 2.5 mm; cone height: 0.5 mm). f) The Nyquist plot of the sensor in PBS containing 5 mm H_2_O_2_ with various PANI deposition currents. g) The CV curves of the sensor in PBS containing 5 mm H_2_O_2_ with various PANI deposition currents (deposition time: 10 min). h) The sensitivity of the sensor for detecting H_2_O_2_ at various potentials (*n* = 3). i) The CV curves of the sensor in PBS containing 5 mm H_2_O_2_ at various scanning rates. j) The sensing performance to detect H_2_O_2_ with successive additions to PBS (*n* = 3).

**Figure** **2**a illustrates the fabrication steps for the transient, partially dissolvable microneedle‐microtube sensing device. 3D‐printed microtubes are first fabricated as building blocks for the sensing electrodes (step 1). Then, thin Au and Ag films are deposited on the microtubes (step 2), followed by the chlorination of Ag to Ag/Cl as the reference/counter electrode (step 3). Figure S4 (Supporting Information) shows the SEM image and EDS mapping analysis of the Au and Ag/AgCl electrodes, indicating that the electrodes were uniformly formed on the sidewall of the microtube. Furthermore, PB was deposited to functionalize the Au electrode as the working electrode (step 4). Further, to stabilize the sensing performance, the working electrode on the sidewall of the microtube was electrodeposited with the polyaniline layer (step 5), followed by the sequential depositions of GOD (step 6), glutaraldehyde (GA) (step 7), thermoplastic polyurethane (TPU) (step 8), and polyvinyl alcohol/polyethylene glycol (PVA/PEG) membrane (step 9) with drying in between. Figure S5 (Supporting Information) shows the photographs the working electrode on the sidewall of the microtube during deposition of the aforementioned substances. First, the tube orifice is sealed with hot melt adhesive, then the microtube is inverted and placed in the electrodeposition cell to avoid solution modification on the inner wall of the microtube during this process. The polyvinyl pyrrolidone/polyvinyl alcohol (PVP/PVA) composite with high biocompatibility has been used for transdermal drug delivery^[^ ^30^ ^]^ and extraction of interstitial fluid.^[^ ^31^ ^]^ In this study, transient PVP/PVA microneedles were fabricated and integrated with non‐dissolvable microtubes to form the device. A composite structure of microneedles and microcylinders in PVP/PVA was fabricated via soft lithography using a negative PDMS mold. Finally, the microtube sensing device and dissolvable microneedles were integrated into the biosensing device.

Figure 2b–d shows SEM images of a single microtube, a microneedle, and its integrated form. The tip of the microneedle is cone‐shaped to guide the insertion of the microtube into the skin. Different sizes of the microtubes and dissolvable microneedles were also designed and fabricated (Figure S6, Supporting Information). The dissolution process of the microneedles in phosphate buffer solution (PBS) and stimulated interstitial fluid was studied (Figures S7 and S8, Supporting Information). Four microneedles dissolved within 10 min, and the substrate was almost completely dissolved within 50 min. Figure 2e exhibits a photograph of the dissolvable microneedle array with good solubility and biocompatibility. As shown in Figure S9 (Supporting Information), the mechanical characterization indicates that both dissolvable microneedles and microtubes exhibit excellent mechanical performance. Figure 2f and Figure S10 (Supporting Information) depict the electrochemical impedance spectra of the biosensor in PBS containing 5 mm H_2_O_2_ at different polyaniline (PANI) deposition currents. As the deposition current increases (Figure S11, Supporting Information), the electron transfer resistance of the electrode increases, indicating that the thickness of the PANI layer also increases. As illustrated by the cyclic voltammetry^[^ ^22d^ ^]^ curves in Figure 2g, the current window shrinks with increasing deposition current, and extending the deposition time also shrinks the current window (Figure S12, Supporting Information).

A study of the sensor's sensitivity at different potentials showed that the maximum relative response for detecting H_2_O_2_ was obtained at ‐0.1 V (Figure 2h). Therefore, additional amperometric analyses of H_2_O_2_ and glucose were conducted using a constant voltage of ‐0.1 V. Figure 2i shows the CV curves of the sensor for H_2_O_2_ in PBS at various scanning rates. The linear relationship between the oxidation peak currents and the square roots of the scanning rates is consistent with the Randles–Sevcik equation, indicating that the H_2_O_2_ sensing process is diffusion‐controlled.^[^ ^32^ ^]^ Figure 2j shows the sensing performance for detecting H_2_O_2_. It has a linear range of 0.8 to 48 mM and a sensitivity of 0.0714 ± 0.0027 µA mM^−1^. These results imply that the sensor is expected to detect hyperglycemia effectively because it can detect the H_2_O_2_ concentration accurately.

### 2.3 In Vitro Glucose Sensing Performance of the Biosensor

Before performing in vivo glucose sensing, in‐vitro glucose sensing was evaluated to determine the sensing performance, including the detection range, sensitivity, response time, selectivity, and stability.

**Figure 3:**
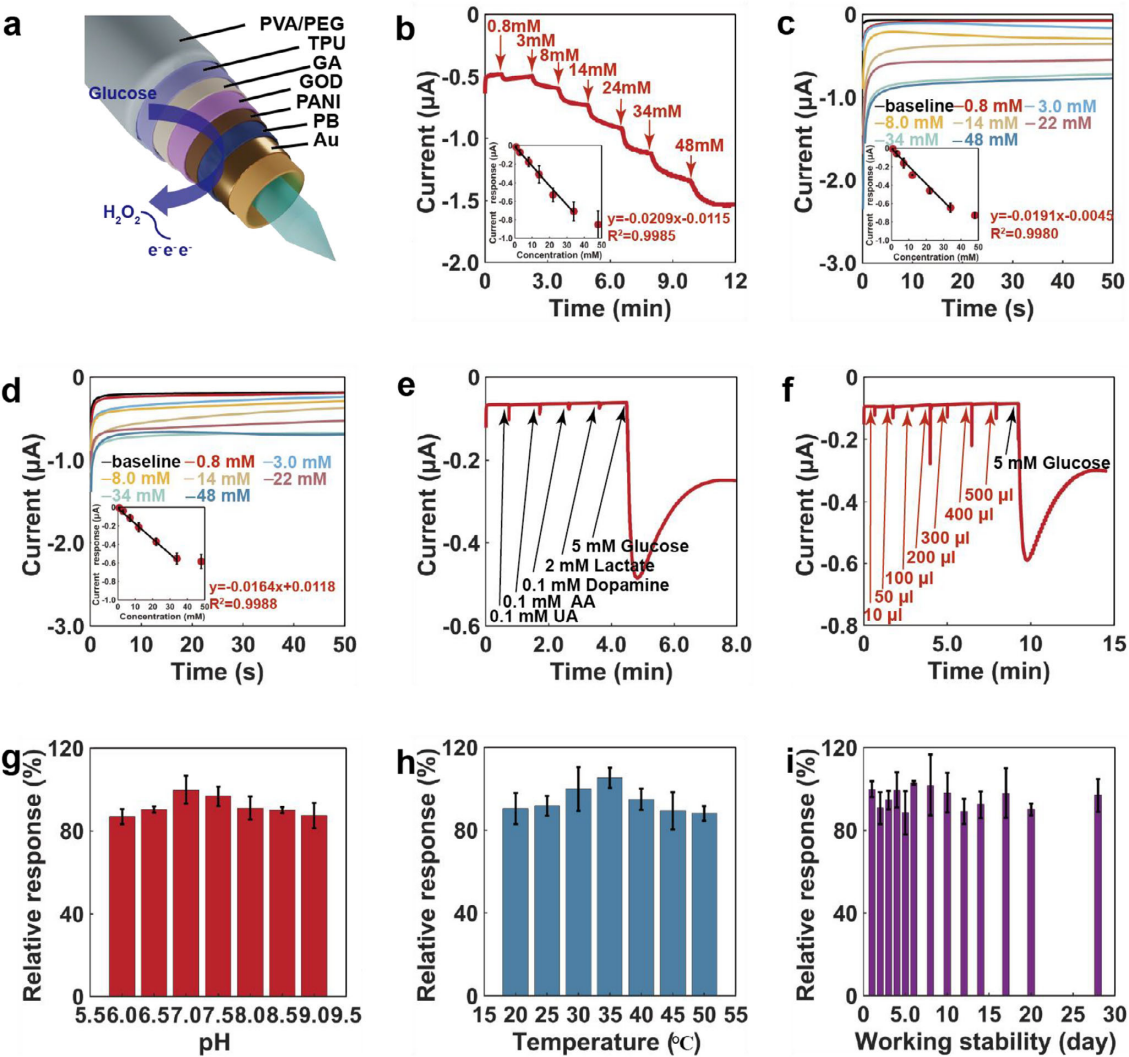
In vitro sensing performance for detecting glucose. a) Scheme of multilayer‐layer structure on the working electrode. b) Characterization of the sensor during rapid addition of glucose to PBS (*n* = 3). c) Baseline response characteristics of the sensor to detect glucose in PBS (*n* = 3). d) Baseline response of the sensor to glucose in simulated interstitial fluid (*n* = 3). e) Electrical interferences to the sensor by various electroactive substances. f) Insulin dose effect on the sensor. g) Stability of the sensor to pH changes (*n* = 3). h) Stability of the sensor to changes in temperature (*n* = 3). i) Storage stability of the sensor in PBS containing 5 mm glucose at room temperature (*n* = 3).

The sensor's working electrode had a multi‐layered structure to ensure biosensor stability (**Figure** **3**a). The PB^[^ ^33^ ^]^ layer was used as an electron mediator to lower the detection potential, increase the selectivity, and expand the detection range. Figure S13 (Supporting Information) shows CV curves for the electrodeposition of the PB layer, in which clear oxidation and reduction peaks were observed. Figure S14 (Supporting Information) shows SEM images of each layer, and Figure S15 (Supporting Information) shows the EDS point analysis of the working and counter/reference electrodes. There were obvious peaks of Au on the original working electrode and Ag and Cl on the counter/reference electrode. Fe and C were also evident after PB (Fe_4_[Fe(CN)_6_]_3_) and PANI (C_6_H_7_N) deposition, respectively. The PANI membranes interconnect to form a network structure, providing sufficient active surfaces for subsequent enzyme immobilization, which facilitates high‐density enzyme loading.^[^ ^34^ ^]^ The goal of the GA was to cross‐link the immobilization of GOD. The TPU membrane served as a barrier to stop enzyme leakage and regulate the amount of glucose diffused to the surface of the working electrode.^[^ ^35^ ^]^

The performance of the microtube biosensor in detecting glucose was evaluated using amperometric glucose measurements in PBS and stimulated interstitial fluid. Figure S16 (Supporting Information) depicts the CV curves of the sensor measured at different scan speeds in PBS containing 5 mm glucose. The linear relationship between the peak current and the square root of the scan rate further suggests that diffusion is responsible for regulating glucose detection. Figure 3b illustrates the sensor's response to different glucose concentrations in PBS. The linear detection range is from 0.8 to 34 mm with a sensitivity of 0.0209 ± 0.0027 µA mm ^−1^. The blood glucose levels of both diabetics and healthy individuals can be detected within this range. The average response time of the sensor to different glucose concentrations was 95.54 s ± 16.59 s. Figure 3c shows the current baselines of the sensor at various glucose concentrations. The sensing device performs similarly to that in Figure 3b with a sensitivity of 0.0191 ± 0.0019 µA mm ^−1^ and a detection range of 0.8 to 34 mm. Figure 3d demonstrates the capability of the sensor to identify glucose in the stimulated interstitial fluid. The results showed a linear range of 0.8 to 34 mm and a calibration curve with a slope of 0.0164 ± 0.0016 µA mm. The difference in viscosity between the stimulated interstitial fluid and PBS may be responsible for this decrease in sensitivity.

The effect of interference from electroactive compounds such as ascorbic acid, dopamine, uric acid, and lactate on the biosensor was also evaluated (Figure 3e). The results showed that the biosensor had the highest selectivity for glucose compared to other interferents. In addition, insulin solutions may affect the accuracy of glucose sensing in practical applications. The interference effect of different volumes of insulin solution (100 U ml^−1^) on the sensor was investigated, as shown in Figure 3f. The sensor's response to 5 mm glucose was significantly greater than the interference response to the insulin solution.

The sensing devices were evaluated at different pH values, room‐temperature storage times, and temperature conditions. When exposed to 5 mM glucose in PBS, the biosensor displayed the highest relative response (100%) at pH 7.0 and the lowest relative response (86.90%) at pH 6.0 (Figure 3g). The sensing response to 5 mm glucose in PBS was also temperature‐dependent, peaking at 35 °C (105.26%) and bottoming out at 50 °C (88.16%) (Figure 3h). Finally, owing to its multi‐layer design and the improved stability effect of the multi‐layer membrane, the biosensor maintained good stability in PBS containing 5 mm glucose for 4 weeks at ambient temperature. At day 28, the sensor maintained a relative response of 96.94% in PBS (Figure 3i). For comparison, the stability of the sensor was reduced if it contained only the enzyme‐GA layer or lacked one of the PANI, TPU, or PVA/PEG layers (Figure S17, Supporting Information). Even after 50 consecutive measurements of 5 mm glucose in PBS, the biosensor maintained a relative response of 86.94%, demonstrating its superior stability (Figure S18, Supporting Information). Figure S19 (Supporting Information) demonstrates the stability of the Ag/AgCl reference electrode in different chloride ion (Cl^−^) concentrations. Long‐term experiments in synthetic interstitial fluid solution further confirmed the stability of the sensor's Ag/AgCl reference electrode (Figure S20, Supporting Information). These results demonstrate that the sensor can reliably, accurately, and effectively monitor glucose levels.

### 2.4 Fabrication of a Low‐Power, Stable, Compact Electroosmotic Micropump

**Figure 4:**
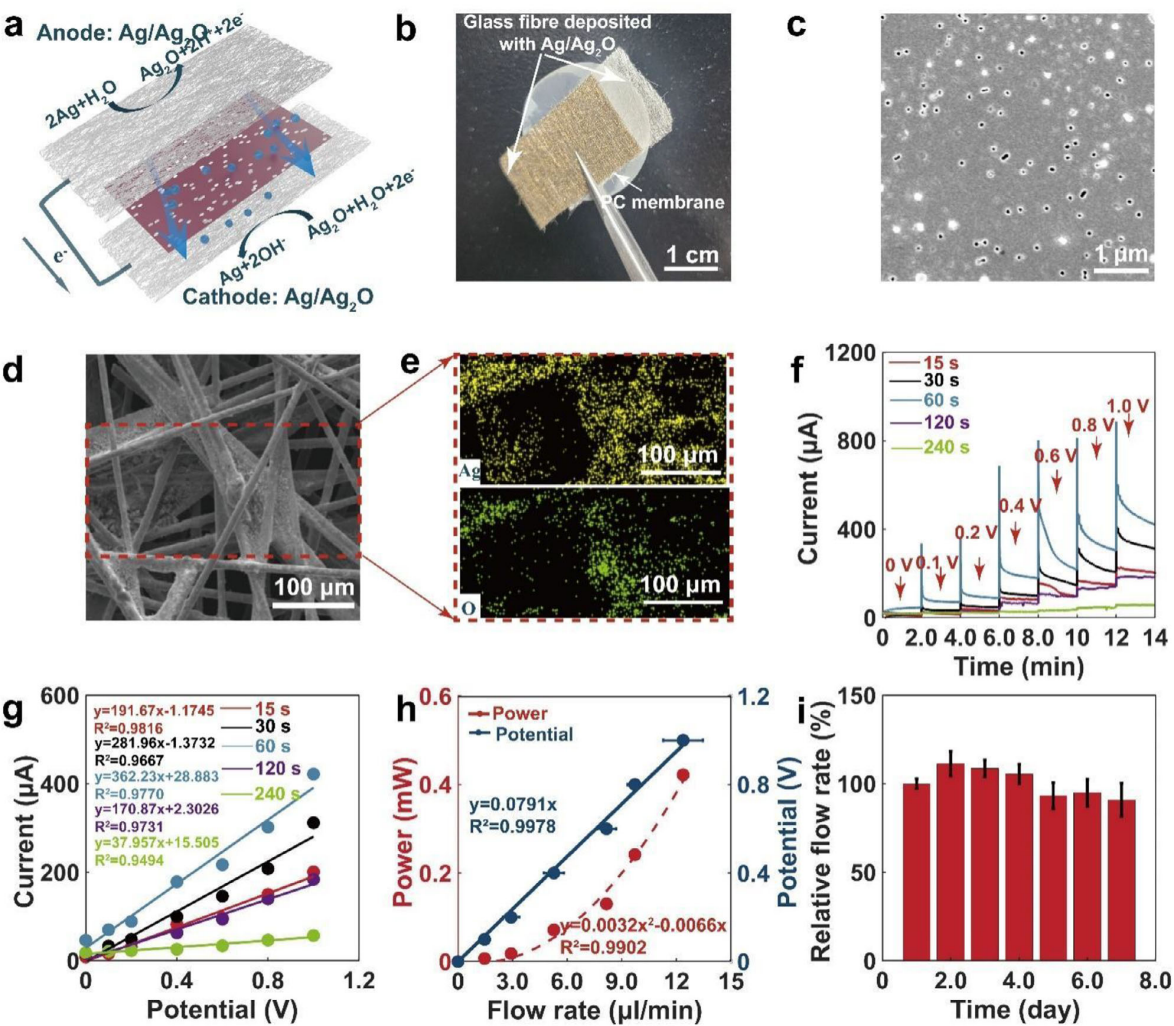
Development and characterization of a low‐power electroosmotic micropump. a) Illustration of the structure and operation principle of the micropump. b) Camera image of the electroosmotic micropump. c) SEM image of the PC membrane. d) SEM image of the Ag/Ag_2_O glass fiber electrode. e) EDS mapping analysis of the Ag/Ag_2_O glass fiber electrode (oxidation time = 60 s). f) Current‐versus‐time curves of the micropump operated at different potentials and different oxidation times for the Ag/Ag_2_O glass fiber electrodes. g) Relationship between the potential and current of the micropump at different oxidation times for Ag/Ag_2_O glass fiber electrodes. h) Power and potential versus flow rate of the micropump for delivering insulin (*n *= 3). i) Stability of the micropump in 7 days (*n* = 3).

Electrically controlled micropumps can provide accurate insulin delivery and are highly desirable for drug delivery. The micropump must fulfill several criteria, including low power consumption, high‐precision stability, and compactness to achieve long‐term and continuous in‐vivo performance for diabetes management. To meet the above requirements, we studied a new electroosmotic micropump for the delivery of a high concentration of insulin, based on a nanoporous polycarbonate (PC) membrane sandwiched between two glass‐fiber substrates, with deposited Ag/Ag_2_O as the anode and cathode (**Figure** **4**a). The protons generated at the anode and those consumed by OH^−^ ions at the cathode do not diffuse through the bulk water phase. Instead, they propagate via water‐bounded P‐OH and Si‐OH groups on the phosphosilicate surface, thereby inducing adjacent water layer movement. This enables directional transport of insulin molecules in solution across the PC membrane.

Electroosmotic micropumps, which are among the simplest, smallest, and most cost‐effective pumps for delivering insulin solutions, are composed of two flow‐through electrodes separated by a porous membrane.^[^ ^36^ ^]^ Although we recently reported wearable electroosmotic micropumps for insulin delivery, these operate at relatively high voltages (10 V), sufficient to electrolyze water in the insulin solution to produce H_2_ and O_2_ while consuming high power (2.6 to 3.2 mW).^[^ ^20^, ^23^ ^]^ It is challenging to maintain a steady pumping rate because the generated bubbles adhere to and clog the pores of the electrodes and membranes and consume relatively large amounts of power. The energy consumption of the pump dominates the overall energy consumption of the closed‐loop system, because the energy consumption of the sensor is only ≈0.1 µW, which is thousands of times lower than the energy consumption of the pump. Therefore, to reduce the overall energy consumption, it is highly desirable to reduce the energy consumption of the micropump.

To minimize power consumption, lowering the power supply and avoiding air bubbles during liquid pumping are key to improving the efficiency of electroosmotic pumps. As shown in Figure 4a, we studied an electroosmotic micropump with a nanoporous polycarbonate membrane sandwiched between two glass‐fiber substrates with deposited silver/silver‐oxide (Ag/Ag_2_O) as the anode and cathode. The two porous Ag/Ag_2_O electrodes were formed by physical vapor deposition of Ag onto the glass‐fiber substrates, followed by the electrooxidation of Ag to Ag/Ag_2_O in NaOH. During the operation of the micropump, the DC voltage between the anode and the cathode was 1 V. Ag was electrooxidized to Ag_2_O at the anode, generating a stream of protons. After passing through the PC membrane, the protons combine with hydroxide anions produced by the cathode reaction to electrically reduce Ag/Ag_2_O to Ag. As shown in the Figure S21 (Supporting Information), the potential difference between the anodic and cathodic reactions occurring on the Ag/Ag_2_O‐coated glass fiber is very small (E = 0.10 V), indicating that reactions 1 and 2 are nearly reversible. In contrast, in a conventional platinum‐electrode electroosmotic system, the redox reactions exhibit a much larger potential difference (E = 2.50 V) between the anode and cathode. Here, most of the applied voltage is consumed by water electrolysis rather than driving liquid flow, thereby reducing the energy efficiency of the pump.

Figure 4b shows a camera image of the entire electroosmotic micropump. The side of each electrode deposited with Ag/Ag_2_O is adhered to both sides of the polycarbonate membrane. Figure 4c shows the SEM image of the polycarbonate membrane with many nanopores. The pore size of the PC membrane is ≈0.1 µm. Figure S22 (Supporting Information) shows the SEM image and EDS spectra of the Ag‐deposited glass‐fiber substrate, indicating that Ag is uniformly distributed on the fibers with almost no O deposited. As the oxidation time increases, the surface of the glass‐fiber substrate gradually darkens, indicating an increase in the Ag_2_O content (Figure S23, Supporting Information). Figure 4d,e shows the SEM images and EDS analysis spectra of the Ag/Ag_2_O electrodes (oxidation time: 60 s). O elements were uniformly distributed on each fiber.

Figure 4f shows the current‐versus‐time curves for releasing insulin solution (100 U ml^−1^) at different potentials. The current at each voltage gradually stabilized during the 2‐min measurement. The 2‐min terminal currents at different potentials also increased linearly with increasing potential (Figure 4g). As shown in Figure 4f,g, and Figure S24 (Supporting Information), as the oxidation time of the Ag/Ag_2_O electrode was increased from 15 to 60 s, the electroosmotic current at the same potential gradually increased. This suggests that as the oxidation time increases, the content of Ag_2_O gradually increases, and more electrons are generated by the electrode reaction at the same voltage, leading to a higher electroosmotic flow rate. However, when the oxidation time of the Ag/Ag_2_O electrode was increased from 60 to 120 s, the increase in the Ag_2_O content led to a gradual decrease in the conductivity of the electrode, and consequently, to a decrease in the electroosmotic current. Therefore, an Ag/Ag_2_O electrode with an oxidation time of 60 s was adopted for further flow‐rate measurements and animal experiments. With this electrode, the current was relatively stable during the continuous operation of the pump for more than 12 h (Figure S25, Supporting Information).

Figure 4h depicts the relationship between the flow rate and the power or potential required for the micropump to release the insulin solution (100 U ml^−1^). The flow rate increases linearly from 1.458 to 12.371 µl min^−1^ when the potential is increased from 0.1 to 1 V. A linear relationship is shown between the insulin flow rate and the micropump potential with a linear regression slope of 0.0791 V/(µl min^−1^). By varying the DC voltage applied to the micropump, the user can consequently vary the rate of insulin release. Furthermore, as the potential increases from 0.1 to 1 V, the power required to deliver insulin increases from 0.007 to 0.422 mW. By measuring the time–current curve of the closed‐loop system operating with a simulated 3.7 V lithium battery using an electrochemical workstation (Figure S26, Supporting Information), it was found that the power consumption was 46.73 mW in standby mode and 47.69 mW when supplying 1 V to the electroosmotic pump. The total power consumption of the entire circuit board increased to 0.96 mW. The micropump requires only a relatively small amount of power (0.422 mW) to achieve the 12.371‑µl min^−1^ insulin flow rate. A square relationship can be observed between the power required by the micropump and the insulin flow rate, with the equation y = 0.0032x2 ‐ 0.0066x, where y is the power and x is the flow rate. When the micropump was integrated with the hollow microneedle array, its insulin flow rate decreased to 9.4655‐µl min^−1^ at 1 V (Figure S27, Supporting Information), which may be due to the capillary force in the tip of microneedles, indicating the superior capability of microtubes for insulin delivery compared to that of microneedles.

In addition, the power to flow rate ratio increases as the potential increases, suggesting that using a lower potential to deliver insulin is beneficial for energy conservation (Figure S28 Supporting Information).This advantage enables the 1500 mAh lithium battery to achieve 116 h of full‐power operation (Figure S29, Supporting Information). The low power of the micropump originated from the Ag/Ag_2_O electrodes. When using the same size of aluminum mesh as the anode and stainless steel mesh as the cathode, the maximum flow rate of the micropump was only 6.545‐µl min^−1^ with a power consumption of 30.7 mW at 10 V (Figure S30, Supporting Information). As can be seen in Figure 4h, to achieve the same flow rate of 6.545‐µl min^−1^, the power consumption of the micropump in the present study was only 0.094 mW, which is ≈326.6 times lower than that of the conventional pump. In addition, the micropump had excellent stability, and at day 7, it maintained its 90.97% relative flow rate (Figure 4i). The accuracy of the micropump's basal flow rate was determined to be 96.16% ± 10.05% over 72 h and 126 measurements, with the measurements conducted at 1 V (Figure S31, Supporting Information). During prolonged full‐power operation, the pump's lifespan can reach up to 4 days (Figure S32, Supporting Information). Moreover, SEM images showed that the pores of the PC membrane were not clogged after 4 days use (Figure S33, Supporting Information). These findings suggest the remarkable efficiency of the micropump in delivering insulin.

### 2.5 In Vivo Blood Glucose Management in Diabetic Rats

Sprague Dawley (SD) rats with streptozotocin (STZ) induced diabetes mellitus were initially selected as experimental subjects to evaluate the in‐vivo performance of the closed‐loop artificial pancreas patch in rats. Because the thickness of the stratum corneum and dermis varies across different skin regions, with significant differences between the back and abdomen. Given that the prone sleeping posture of experimental animals makes abdominal sensor placement unsuitable for long‐term studies due to mechanical stress and detachment risks, our experimental design integrated the sensor module into the dorsal region of the subjects. The dorsal skin of SD rats is more similar to human skin of the arm than that of pigs, although it is slightly thinner.^[^ ^37^ ^]^ HE staining experiments on the dorsal skin of pigs and SD rats confirmed this (Figure S34, Supporting Information).

**Figure 5:**
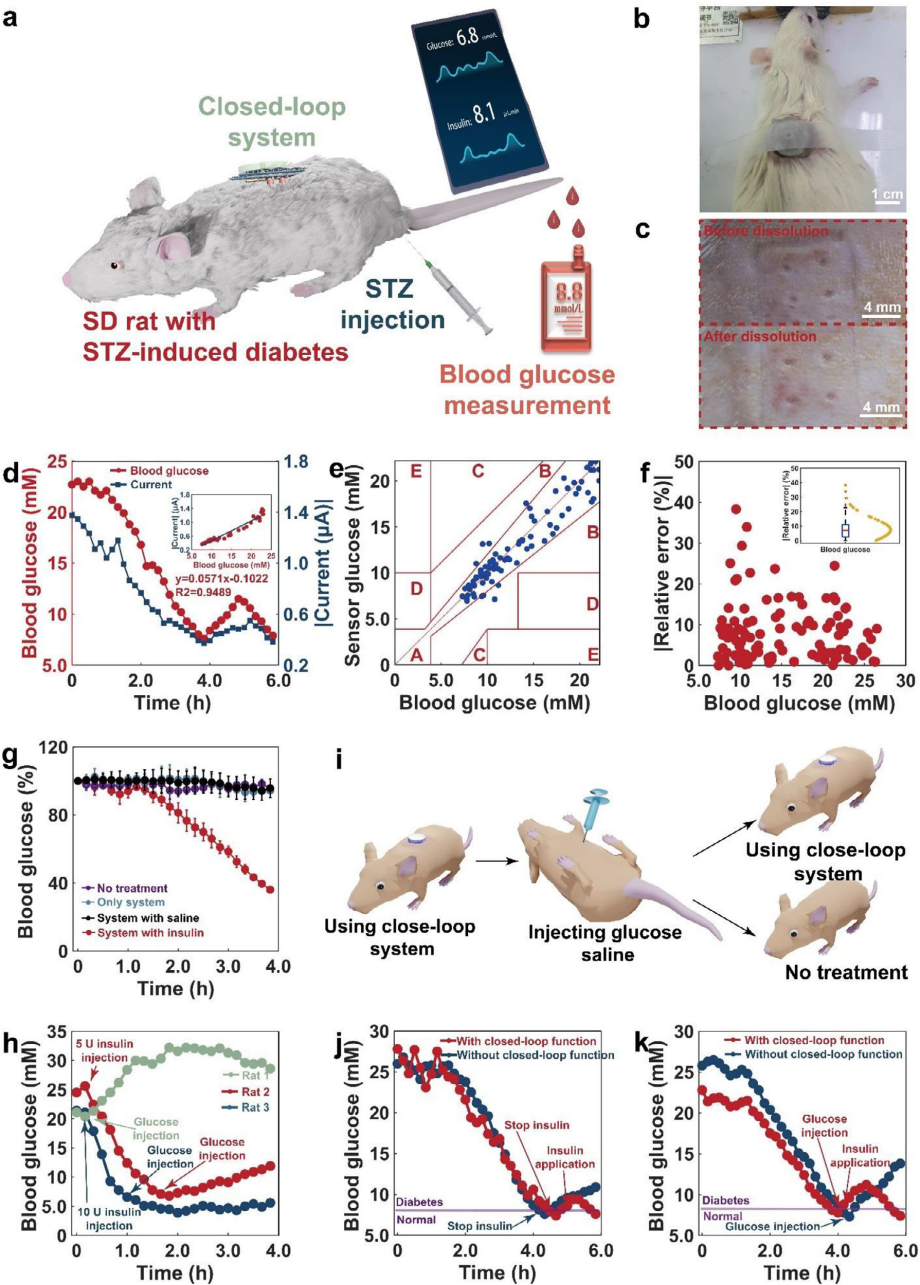
In vivo blood glucose management in diabetic rats. a) Diagram of the system being placed onto the skin of a diabetic rat. b) Photograph of the rat with the system application. c) Pressure marks left on the back skin of a rat after wearing the system. (The microtube was 0.6 mm in height, 0.6 mm in outside diameter, and 0.4 mm in inner diameter. The dissolvable microneedle was 0.3 mm in diameter, 0.8 mm in cylinder height, and 0.2 mm in top cone height.) d) Correlation between the blood glucose level and the sensing current over time. e) Clarke error grid of blood glucose levels obtained by a commercial glucometer and a microtube sensor. f) Glucose sensing error of the sensor in comparison to a commercial glucometer. g) Change in blood glucose level over time under different situations. h) Blood glucose levels change over time when manually infusing single glucose and insulin doses. i) Illustration process of simulating food intake for the closed‐loop rat's blood glucose management. j) Changes in blood glucose levels over time without glucose infusion. k) Changes in blood glucose levels over time with glucose infusion.

**Figure** **5**a shows a closed‐loop device applied to an SD rat intraperitoneally injected with STZ following a diabetes induction procedure.^[^ ^38^ ^]^ The system was secured to the skin on the back of the rat using medical tape and was powered and controlled by a PCB. Blood was drawn from the tail vein, and blood glucose was measured using a commercial glucometer from Sinocare or Sibionics to validate the system's functionality for glucose management.

Figure 5b,c shows camera images of the device immobilized on the skin of the rat's back and the hole it left in the skin, respectively. When the device was first mounted on the skin, the holes it left were conical, consistent with the tip shape of the dissolvable microneedles, and the microtubes also penetrated the skin. After the microneedles were completely dissolved, only the microtubes remained in the skin, leaving cylindrical holes. The skin of rats wearing only microtubes showed visible grooves (Figure S35, Supporting Information). Hematoxylin‐ and eosin‐stained pierced rat back‐skin sections demonstrated that the microneedles and microtubes were successfully inserted into the skin (Figure S36, Supporting Information). The separated sensing and drug delivery two‐step working model was chosen to achieve the closed‐loop control of blood glucose levels in diabetic rats (Figure S37, Supporting Information). By injecting 1 U kg^−1^ doses of insulin, either stored in the reservoir for 7 days or freshly opened from the same batch, into diabetic rats, the hypoglycemic effects were essentially the same in both groups (Figure S38, Supporting Information). These results indicate that insulin maintains good stability at room temperature for at least 7 days. The storage duration fully meets the usage requirements of diabetic patients.

Due to the stable environment in the interstitial fluid, the sensor performed an amperometric measurement for 50 s, before transmitting the final current to the microcontroller of the PCB. If the current exceeded the critical threshold, the micropump would be operated at a constant potential (1.0 V) for 10 min. The sensing and pumping procedures would be repeated alternately until the blood glucose level was lower than the critical threshold. The insulin release was then stopped, and only the glucose level was measured. From the in‐vivo biocompatibility evaluation of the device, when bare microneedles (composed solely of 3D‐printed material) were attached to rat skin for 8 days using non‐biocompatible adhesive tape, significant erythema and edema were observed due to the exposed 3D‐printed microneedles. However, after coating the microneedles with TPU and PVA/PVP layers, the sensor's biocompatibility improved markedly, owing to the enhanced biocompatibility of the multilayer film on the electrodes. Following 8 days of sensor attachment, almost no erythema or edema was observed (Figure S39, Supporting Information). Figure S40 (Supporting Information) presents hematoxylin and eosin (H&E) staining results of skin in rats wearing biosensors for 7 days. Figure S41 (Supporting Information) presents H&E staining results of major organs (heart, liver, spleen, lungs, and kidneys) in rats wearing biosensors for 7 days. Compared with the control group, the histological sections of these organs show no significant changes, indicating that the microneedle material (PVP/PVA) used in the sensor exhibits excellent biocompatibility.

The initial step toward attaining autonomous blood glucose control was to determine the relationship between the sensing current values and blood glucose levels. Figure 5d shows the current feedback corresponding to blood glucose changes in a closed‐loop system worn by rats for 6 h (72 measurement points), alongside blood glucose levels obtained from a commercial glucometer. Because the sensor uses –0.1 V as the detection voltage, the current values are negative. To better visualize the quantitative correlation between current magnitude and glucose concentration in the calibration curve, since analyte concentrations are inherently positive, we adopted absolute values (|Current|) as the label for the Y‐axis. A linear calibration curve was obtained with a slope of 0.0571 µA mM^−1^ and an R^2^ of 0.9489. The Clarke error grid was used to determine whether there was a difference between the blood glucose levels measured by the microtube biosensor and the commercial glucometer (Figure 5e). The clinically accepted zones A and B contained all 108 points from three rats that were equally distributed throughout them. The error of the sensor ranged from 0.013% to 38.3%, and 75% of the points were below 10.9% (Figure 5f). The mean absolute relative difference (MARD) value between the two approaches was found to be 8.3% ± 7.3%. During the experiment, the blood glucose level of rats was decreased and increased by the regulation of the pump. The results demonstrated that the sensor exhibited excellent reversibility, maintained reliable detection even under complex glycemic fluctuations, and met the accuracy limitation requirements of ISO15197:2013.^[^ ^39^ ^]^

A comparative study examined the effects of no treatment, using only microtubes to deliver saline and insulin and no treatment whatsoever (Figure 5g). In the control group, blood glucose levels remained essentially stable. Thus, not using the device, using the device without releasing any fluid, or releasing only saline had little effect on blood glucose management. When the system was administered to rats and insulin was released into the interstitial fluid via the microtube, blood glucose decreased to 36.03% of the starting glucose level within 230 min (n = 4, the results were from 4 different diabetic rats). These findings suggest that the closed‐loop device can intelligently inject insulin solution into the skin and effectively reduce blood glucose levels in diabetic rats.

Figure 5h shows the blood glucose changes in diabetic rats after manual glucose and insulin one‐dose infusions. The first rat was manually administered glucose intraperitoneally with the syringe at minute 20 (green line). Its blood glucose was rapidly increased from 20.4 to 32.2 mm at minute 110, and almost no decrease was observed in the following 2 h, which demonstrated that a large amount of glucose intake at one time would make the blood glucose of diabetic patients rise rapidly and not fall for a period. The second rat was manually administered 5 U insulin subcutaneously at minute 20 (red line). Its blood glucose rapidly decreased from 25.6 to 7.8 mm at minute 90. Then, the rat was also manually administered glucose intraperitoneally, and its blood glucose gradually increased from 7.1 mM at minute 100 to 11.9 mm at minute 230. The third rat was administered 10 U insulin subcutaneously at minute 20 (red line). Its blood glucose rapidly decreased from 21.1 to 7.8 mM at minute 50. Then, glucose was manually administered to the rat intraperitoneally; however, its blood glucose levels showed almost no increase in the following 3 h. The results of the second and third rats proved that injecting a small amount of insulin at once can cause a rapid dip in blood glucose. However, after glucose intake, blood glucose gradually increased again, and a large amount of insulin injection at once carried the risk of hypoglycemia. Therefore, for diabetic patients, it is difficult to decide the precise amount of insulin injected once and to predict the blood‐glucose control effect.

Figure 5j illustrates how the system effectively lowers blood glucose levels in diabetic rats without glucose intake. Considering that insulin takes ≈10 min to lower blood glucose levels in rats, and that microneedles take 80–110 min to dissolve, when the device was first used in rats, the insulin and interstitial fluid gradually dissolved the microneedles over 1.5 to 2 h, during which the blood glucose level remained relatively stable. Afterward, blood glucose gradually decreased over 2 to 2.5 h to reach a critical value (8.3 mm). Insulin injections were then abruptly terminated. Thereafter, one scenario (black and green lines) was performed without any medical intervention, and due to the lack of insulin therapy, the blood glucose level gradually increased to 10.9 mm at minute 350. In the second scenario (red line), insulin injections were automatically resumed when the blood glucose increased to 8.6 mm (above the critical value of 8.3 mm) at minute 300. At minute 350, the blood glucose dropped to 7.6 mm. These results suggest that the system can effectively achieve closed‐loop intelligent management in the absence of glucose intake.

Figure 5k exhibits the impact of the closed‐loop artificial pancreas patch on the blood glucose levels of diabetic rats administered manually intraperitoneal glucose injections to simulate swallowing food. As illustrated in Figure 5i, two rats were first administered the system. When their blood glucose levels were normal, the two rats were manually administered glucose intraperitoneally. Afterward, one rat continued to be treated with the system, whereas the other rat received no treatment. Similar to Figure 5h, the blood glucose of the two situations remained relatively stable at the beginning of 1.5 to 2 h. Insulin injections were stopped immediately when the blood glucose of the first rat dropped from 25.8 to 7.8 mm below the threshold (8.3 mmol) at minute 250 (black‐green line). Glucose (1 g kg^−1^) was manually injected intraperitoneally when the blood glucose level fell to 7.3 mm at minute 260. Without insulin, blood glucose gradually increased, reaching 13.8 mm at minute 350. The blood glucose level in the second case dropped from 22.8 to 8.1 mm (red line) at minute 230, which was below the threshold level (8.3 mm), and the insulin infusion was automatically stopped. This was similar to the experience of the first rat. Glucose was also manually administered intraperitoneally at minute 240 when the blood glucose level dropped to 7.8 mm. The closed‐loop device immediately started the insulin infusion when the blood glucose level exceeded the 8.3 mm threshold at minute 250. The blood glucose level continued to rise because only a small amount of insulin was initially injected. At minute 290, blood glucose peaked at 11.2 mm and then dropped to 7.9 mm at minute 340.

These findings indicate that the system can successfully pierce the skin of rats, which is much closer to human skin than that of pigs and can successfully automate the control of blood glucose levels in diabetic rats in a closed‐loop fashion in a short time.

### 2.6 In Vivo Blood Glucose Management in Diabetic Pigs

Bama pigs with STZ‐induced diabetes were chosen as experimental subjects to test the in‐vivo performance of the closed‐loop system in large animals. The diabetic pigs have similar endogenous insulin levels (25.78 ± 2.19 μIU ml^−1^ for the first pig, 21.65 ± 1.10 μIU ml^−1^ for the second pig, 18.59 ± 3.84 μIU ml^−1^ for the third pig, *n* = 3 for each pig) to healthy pig (23.72 ± 0.56 μIU ml^−1^, *n* = 3) and high glucose levels. Compared to diabetic rats, pigs weigh 25–37 kilograms, which is fairly close to human weight.

**Figure 6:**
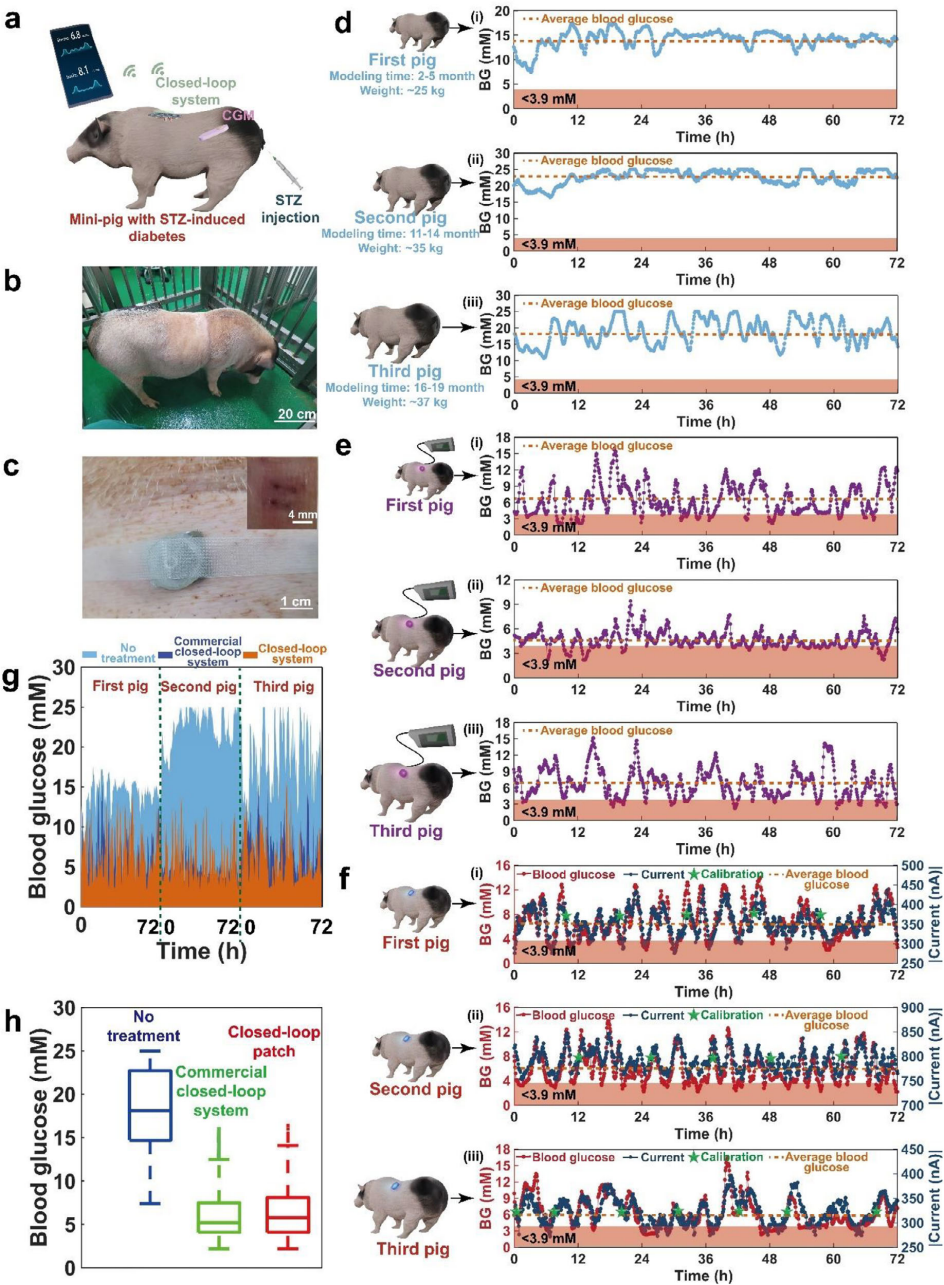
In vivo blood glucose management in diabetic pigs. a) Diagram of the system being worn on a diabetic pig. b) Photograph of a Bama pig. c) Photograph of the Bama pig skin with the system application and the holes left on the skin. d) Blood glucose changes of three pigs without any treatment over 3 days (The blue line represents the blood glucose measured by the commercial CGM, and the orange line represents the average blood glucose). e) Blood glucose changes of three pigs with the commercial closed‐loop system over 3 days (The purple line represents the blood glucose measured by the commercial CGM, and the orange line represents the average blood glucose). f) Blood glucose and measured current changes of three pigs with the closed‐loop system over 3 days (The red line represents the blood glucose measured by the commercial CGM, the black‐green line represents the current measured by the biosensor, the green pentagram mark represents the calibration time, and the orange line represents the average blood glucose). g) Blood glucose levels of the three pigs under different conditions over 3 days. h) Distribution of blood glucose levels of all three pigs under different conditions in 3 days.

**Figure** **6**a shows the closed‐loop artificial pancreas patch applied to the Bama pig that was injected intraperitoneally with STZ to induce diabetes. The patch, secured to the skin on the back of the pig with medical tape and an elastic bandage, is also powered and controlled by a PCB, which communicates wirelessly with a smartphone via Bluetooth to transmit sensing data and commands. To confirm the system's effectiveness in controlling blood glucose, a commercially available CGM system was applied to the pig to detect its blood glucose levels.

Figure 6b,c shows the camera images of the pig and the skin on its back after the application of the system. Owing to the much thicker and more rigid skin of pigs than that of humans and rats,^[^ ^27^, ^40^ ^]^ stain‐steel needles (1.6 mm in diameter) were used to puncture holes in the pig's skin to guide our devices when penetrating the skin (back skin thickness: 3 to 6 mm). The inset in Figure 6c shows the holes left in the pig skin. Pigs of different weights and disease severity were used to simulate patients with diabetes.

Figure 6d–f shows the changes in blood glucose in pigs under different conditions. The pigs weighed ≈25–37 kg, and the diabetes modeling time was ≈2–16 months. Since the glucose detection range of the commercial CGM was from 2.2 to 25 mm, the maximum blood glucose value observed in experiments in pigs was 25 mm. All blood glucose levels of the three pigs exceeded the healthy threshold (7.0 mm) for three days without any treatment (Figure 6d). The blood‐glucose change range was from 7.4 to 17.5 mm for the first pig, from 16.4 mm to more than 25 mm for the second pig, and from 10.7 mm to more than 25 mm for the third pig. The average blood glucose levels were 14.1 mm for the first pig, 22.6 mm for the second pig, and 18.8 mm for the third pig, as shown by the orange dashed lines in Figure 6d.

Further, a commercial closed‐loop system with the AndroidAPS control algorithm was applied in pigs, with basal insulin rates, insulin sensitivity factors (ISF), and insulin‐to‐carbohydrate (ICR) ratios entered into the AndroidAPS control algorithm (Figure 6e). The basal insulin rate settings were optimized for the pigs, resulting in 0.6 U h^−1^ for the first pig, 1.5 U h^−1^ for the second pig, and 1.2 U h^−1^ for the third pig (Figure S42, Supporting Information). The ISF and ICR values for each pig were studied, resulting in ISF values of 1.40 ± 0.08, 2.16 ± 0.05, and 2.17 ± 0.10 mM per unit insulin for the first, second and third pigs, and ICR values of 30.00, 37.50, and 37.50 g carb per unit insulin for the first, second and third pigs (Table S1, Supporting Information). Under the commercial system, the time in tight range (TITR, 3.9–7.8 mm) in 3 days was 51.91% for the first pig, 78.84% for the second pig, and 52.14% for the third pig. The time in range (TIR, 3.9–10.0 mm) was 71.79% for the first pig, 79.42% for the second pig, and 73.64% for the third pig. The time below range (TBR, <3.9 mm) was 16.30% for the first pig, 20.58% for the second pig, and 14.68% for the third pig. The average blood glucose levels declined to 6.5 mm in the first pig, 4.6 mm in the second pig, and 6.8 mm in the third pig, as shown by the orange dashed lines in Figure 6e. These performances in pigs using commercial systems were not as good as those typically seen in humans, most likely because pig skin is thicker and harder than human skin, making it unsuitable for the installation of CGM and insulin pump.

Figure 6f shows the results of glycemic management in pigs using a closed‐loop patch worn on pigs for 72 h (864 measurement points). Dawn and Somogyi diabetes phenomena and food intake may elevate blood glucose levels.^[^ ^41^ ^]^ The trend of current changes measured by the biosensor was relatively consistent with the blood glucose fluctuations. Considering the weight and varying severity of diabetes in each pig, personalized control algorithms (Figures S43 and S44, Supporting Information) were designed and applied to optimize blood glucose management for each pig, resulting in excellent outcomes. A mobile application was designed to control the system and display blood glucose data (Figure S45, Supporting Information). To achieve higher accuracy, the biosensor was calibrated ≈every 12 h to adjust the calibration curve between the current measured by the biosensor and the blood glucose measured by the commercial CGM with the control algorithm shown in Figure S46 (Supporting Information), or to adjust the threshold current value when the blood glucose was ≈7.0 mm, as measured by the commercial CGM with the control algorithm in Figure S47 (Supporting Information). As depicted in Figure 6f‐i, for the first pig utilizing an optimized control algorithm illustrated in Figure S43 (Supporting Information), the TITR over 3 days was 51.10%, the TIR was 67.63%, the TBR was 15.95%, and the average blood glucose level was 6.8 mm. As shown in Figure 6f‐ii, for the second pig utilizing an optimized control algorithm illustrated in Figure S43 (Supporting Information), the TITR in 3 days was 58.50%, the TIR was 68.F%, the TBR was 21.04%, and the average blood glucose level was 5.9 mm. As shown in Figure 6f‐iii, for the third pig utilizing an optimized control algorithm illustrated in Figure S44 (Supporting Information), the TITR in 3 days was 51.21%, the TIR was 67.51%, the TBR was 22.20%, and the average blood glucose level was 6.2 mm. The injection time for administering insulin at different voltages are depicted in Figures S46 and S47 (Supporting Information). Furthermore, with other studied control algorithms, these values for the pigs could vary at certain extend (Figures S48–S51, Supporting Information).

The Clarke error grid was used to analyze the differences between the blood glucose levels measured by the sensor and the commercial CGM in the pigs. 93.76% (811/865, first pig), 94.91% (821/865, second pig), and 92.83% (803/865, third pig) of the points from the sensing results were located in clinically accepted zones A and B (Figure S52, Supporting Information). The experimental results showed that under the control of the closed‐loop system, the blood glucose levels of pigs fluctuated within a healthy range for 72 h, clearly demonstrating that the sensor had good reversibility and excellent detection capability in a complex glycemic environment. The MARD value between the biosensor and the commercial CGM was found to be 56.8% ± 23.5% for the first pig, 30.5% ± 25.6% for the second pig, and 36.1% ± 30.4% for the third pig. The main sources of blood‐glucose measurement errors include detection noise caused by pig movement, interference caused by insulin release and insufficient absorption, and noise during wireless Bluetooth transmission by the PCB. The MARD of a commercial CGM in human trials was reported to be 8.8%.^[^ ^42^ ^]^ However, the MARD between two commercial CGMs in the same pig was measured in this study, ranging from 22.5% ± 13.7% to 42.8% ± 43.3% over 72 h. This difference may be due to the pig's skin being much thicker and harder than human skin. It is expected that the biosensor could achieve comparable accuracy to a commercial CGM on the human body.

In addition, although glucose sensing occurs at the pumping site and several factors may affect glucose sensing in interstitial fluid, such as the perturbation of interstitial glucose levels by local insulin delivery, the dilution of interstitial fluid glucose by insulin, and insulin interference in amperometric measurements, in this study, the sensing accuracy has not been adversely affected by these factors, which is consistent with the reported literature.^[^ ^43^ ^]^ The reasons for the high accuracy may be the negative cooperativity and competition between insulin absorption and local action,^[^ ^43d^ ^]^ slow glucose uptake into cells in the skin interstitial fluid,^[^ ^44^ ^]^ as well as the use of mediator‐based sensors with a low working potential to avoid insulin interference in amperometric measurements.^[^ ^33^ ^]^ Therefore, the development of a dual device for sensing glucose and insulin delivery has been demonstrated to be feasible.^[^ ^43d^ ^]^ Firmer fixation of the device, development of smarter control algorithms, and further improvements in system integration may lead to more accurate glucose detection.

Further, there is a 15–25 min time lag between between the closed‐loop insulin delivering system and the monitoring‐only system (Figure S53, Supporting Information). A 10–20 min time lag was also observed between the sensing current response and the CGM, as indicated by the red lines and black‐green lines in Figure 6f‐i–iii and Figure S54 (Supporting Information). The time delay between the CGM and real blood glucose was estimated to be 5–20 min.^[^ ^45^ ^]^ Therefore, the time delay between the sensor and real blood glucose is estimated to be 15–40 min, which is consistent as the reported value.^[^ ^46^ ^]^ Furthermore, the insulin absorption pharmacokinetics and pharmacodynamics profiles have been studied in three pigs with a 10‐min continuous insulin release using the closed‐loop system (Figure S55, Supporting Information). The time to maximum concentration (tmax) of insulin in blood was 90–120 min, and the onset time of insulin was 40–60 min, which were both longer than those by the direct subcutaneous insulin injection.^[^ ^47^ ^]^ This is probably because the penetration depth of the patch into the skin was shorter than that of subcutaneous insulin injection. Additionally, the thickness of the skin where the patch was applied, the density of capillaries, and the depth of the puncture holes also affected the absorption of insulin. Moreover, when the patch was applied to the pig, external continuous or instantaneous pressure had minimal effect on the biosensor (Figures S56 and S57, Supporting Information).

Figure 6g shows the difference in blood glucose levels for each pig at almost every different time point under different conditions. Under the closed‐loop patch, the blood glucose levels of each pig at every time point were significantly lower than those under no treatment, yet similar to those with the commercial closed‐loop system. The average blood glucose levels of the pigs using the patch were 0.3 mm higher for the first pig, 1.3 mm higher for the second pig, and 0.6 mm lower for the third pig than those using the commercial closed‐loop system. These results suggest that the closed‐loop patch is comparable to the commercial system in achieving similar glucose levels.

Figure 6h depicts the blood glucose distribution of three pigs under different conditions over three days. When these pigs received no treatment, the TITR (3.9–7.8 mm) over three days was 0.19%, the TIR (3.9–10.0 mm) was 1.62%, and the TBR (<3.9 mm) was 0%. Under the commercial closed‐loop system, the TITR for the three pigs over three days was 60.96%, the TIR was 74.95%, and the TBR was 17.19%. With the closed‐loop patch, the TITR for the three pigs over 3 days was 53.60%, the TIR was 67.98%, and the TBR was 19.73%. It is evident that when the patch is applied to three pigs, the blood glucose fluctuation is altered within the usual range and comparable with the commercial system. These results suggest that the closed‐loop patch can maintain blood glucose levels within the normal range in pigs with varying diabetes severities most of the time, and its capability and performance for managing blood glucose are comparable to that of a commercial CGM.

The advantages of this study over commercially available diabetes closed‐loop systems are its ultra‐small size and low cost (Table S2, Supporting Information). In contrast to previously reported closed‐loop systems, this study miniaturized both the sensing and pump overall systems, resulting in a wearable device that can also accurately maintain blood glucose levels in large animals within a safe range over several days. This study achieved high accuracy and sustained performance by utilizing a novel sensing and pump approach, both of which are electrically controlled, in addition to dramatically reducing the cost to less than $10 (Table S3, Supporting Information).

## 3 Discussion

Recent advances in digital health and wearable electronics are transforming conventional hospital disease management to home healthcare management. Here, we demonstrated for the first time an integrated wearable closed‐loop artificial pancreas patch for continuous monitoring and regulating of blood glucose in diabetic rats and pigs, verifying that the patch can be used for several consecutive days in pigs. In addition, the total volume of the entire system is ≈2 cm^3^ and costs ≈$10.

We demonstrated the miniaturization of the entire integrated and wearable closed‐loop mini‐patch by designing partially transiently dissolvable microneedles incorporated with microtubes and system‐level integration with a glucose sensor, a miniaturized insulin micropump, and a wireless PCB. In terms of the device dimensions, we developed the smallest such device on account of the combined miniaturization of the sensor, pump, and PCB. Further, the system can be easily pierced into the skin using a partially transiently dissolvable microneedle‐microtube structure, and the tip of the PVP/PVA microneedle then dissolves, leaving a tubular structure to enter the skin, which is safer for skin tissues and avoids possible breakage of the microneedle and damage to the tissues. With the advancements and optimization in manufacturing approaches,^[^ ^48^ ^]^ such as enhanced 3D printing and injection molding, large‐scale manufacturing is expected to be feasible.

The wearable patch can achieve not only highly stable glucose sensing but also low‐power, highly stable insulin delivery by designing and fabricating new sensing and pumping electrodes, which are crucial for minimizing the power consumption and long‐term continuous operation of the system‐resulting from the multi‐layer construction of the working electrode with diverse biocompatible polymer membranes. The biosensor was favorable owing to its outstanding sensing stability—with the storage in glucose solution at room temperature for more than four weeks. By constructing the Ag/Ag_2_O glass‐fiber electrode for the electroosmotic micropump, the micropump could be operated at low potentials (<1 V) to deliver insulin through the microtube without gas generation, and the power consumption of the pumping system was significantly reduced ≈100‐fold to only 0.422 mW.

We have successfully demonstrated for the first time a continuous operation on both small and large animals, specifically pigs, for continuous monitoring and regulation of blood glucose, a critical step toward the implementation of wearable closed‐loop devices in human diabetes patients. The new closed‐loop artificial pancreas patch was successfully applied to diabetic rats and pigs to control their blood glucose levels. The skin of pigs (2–7 mm) is much thicker than human skin (0.5–2 mm), and is difficult to puncture. Thus, diabetic rats were first studied to demonstrate that our system can puncture the skin of diabetic rats (0.2–1 mm), which is very similar to that of humans, and that the closed‐loop system can efficiently control blood glucose in diabetic rats for several hours. On the other hand, the body weight and physiological metabolism of pigs are very similar to those of humans, and pigs were studied to demonstrate that the closed‐loop artificial pancreas patch can control blood glucose levels in large animals for over 3 days continuously. Compared with the commercial closed‐loop system, this patch can maintain the blood glucose levels within the safe range for a similar duration. The TITR (3.9–7.8 mm) and TIR (3.9–10 mm) of the three pigs over 3 days can reach 53.60% and 67.98%, respectively, whereas these values are 60.96% and 74.95% under the commercial system.

These results strongly suggest that the integrated wearable closed‐loop artificial pancreas patch, with a miniaturized size, low‐power consumption, and highly stable performance, holds great potential to personalize, precisely monitor, and regulate human blood glucose. Although the closed‐loop patch has strong application prospects and practical value, it has not been clinically verified in actual diabetic patients. For clinical settings and human applications in the future, the device should be integrated with better and smarter control algorithms, such as a proportional‐integral‐derivative (PID) controller and the model predictive control (MPC) approach,^[^ ^49^ ^]^ to prevent blood glucose fluctuations from exceeding the safe range and avoid possible insulin leakage from the skin due to insulin accumulation in the dermis. Additionally, the PCB could be redesigned by professional engineers to make it smarter, more intelligent, low‐power, miniaturized, and user‐friendly. Improvements in blood glucose sensing accuracy should be pursued by changing the structure of the biosensor, minimizing detection noise caused by pig movement and wireless Bluetooth transmission, reducing interference caused by insulin release and insufficient absorption, and ensuring firmer fixation of the device.

Furthermore, considering the differences in body weight, metabolism, skin thickness, and mechanical properties between the human body and diabetic rats and pigs, there may be additional challenges and difficulties to overcome in practical application. For example, the absorption of insulin differs between animals and humans, as does the amount needed to maintain diabetes. Additionally, the severity of diabetes at the same glucose level varies between animals and humans, and the difficulty of penetrating the skin differs between pigs and humans. The system needs further optimization and human clinical trials before entering the commercial market. Considering all these optimized processes and the time needed for FDA approval,^[^ ^1^ ^]^ it is estimated to be 3–5 years for the device to be practically used in the future. We expect that this work would offer important contributions to digital health and wearable devices for diabetes patients, and have the potential to revolutionize conventional diabetes management toward at‐home healthcare.

## 4 Experimental Section

### Fabrication of the Partially Transiently Dissolvable Microneedle‐Microtube Structure

The microtubes were fabricated using a 3D printer (S140, BMF Precision Technology Ltd., China, or HALOT‐SKY 2022, Creativity Technology Ltd., China). Microtubes of different sizes were designed and fabricated (Figure 2b; Figure S3, Supporting Information); the microtube height ranged from 0.6 to 2.3 mm, and the outer diameter ranged from 0.6 to 1.5 mm. The center distance between two adjacent microtubes in a 2 × 2 array was 4 mm. The substrate measured 1 cm × 1 cm and was 0.2 mm thick.

Polyvinylpyrrolidone (PVP) and polyvinyl alcohol (PVA) were completely dissolved in deionized water and left at 90 °C for 2 h to make a mixed solution (PVP: 165 mg mL^−1^, PVA: 15 mg mL^−1^). After cooling to room temperature, the solution was cast onto a PDMS mold. After drying at room temperature for two days, the dissolvable microneedles were peeled off from the PDMS mold. The microneedles were arranged in 2 × 2 arrays with a distance of 4 mm between neighboring microneedles. Each microneedle was cylindrical and conical. Dissolvable microneedles of different sizes were designed and fabricated (Figure 2c; Figure S3, Supporting Information); the microneedle heights ranged from 1.0 to 3.0 mm, and the diameters ranged from 0.3 to 0.9 mm. The base of the dissolvable microneedle was 1 cm × 1 cm in size and 2 mm thick.

### Construction of the Microtube‐Based Biosensor

The glucose sensor electrodes, including a working electrode in Au‐PB and a reference/counter electrode in Ag/AgCl, were fabricated on the sidewalls of the microtubes. Ti/Au (20 nm/200 nm) was sputter‐coated onto all the microtubes to obtain two electrodes, one of which was the working electrode. A layer of silver (200 nm) was then deposited on the other electrode, which was subsequently submerged in 50 mm ferric chloride (FeCl_3_) solution for 10 s to form the Ag/AgCl counter/reference electrode. In addition, to remove impurities and activate the Au electrode, the microtubes were then submerged in 0.1 m H_2_SO_4_ solution, and CV was performed from 0.2 to 1.2 V for 20 cycles at a scanning rate of 1 V s^−1^. Then, hot‐melt glue was used to plug the microtubes. Further, to deposit a PB layer on the Au electrode surface, the microtubes were immersed in a freshly prepared solution containing 2.5 mm FeCl_3_, 100 mm KCl, 2.5 mm K_3_Fe(CN)_6,_ and 100 mM HCl, and CV was performed from ‐0.15 to 0.3 V for 8 cycles at a scan rate of 20 mV s^−1^. To stabilize the PB layer, the microtubes were then immersed in 0.1 M KCl/HCl solution, and CV was performed from ‐0.2 to 0.5 V for 4 cycles at a scan rate of 50 mV s^−1^.

To immobilize GOD, the PANI layer was electropolymerized in 0.4 M aniline/0.1 M HCl solution at a continuous current of 0.1 mA for 10 min at the working electrode. The microtubules were then inverted in a 3d‐printed container, 20 µl of GOD solution (50 U µl^−1^) was then applied as the enzyme layer and dried at room temperature for ≈1 h. The microtubules were then placed in another container containing 20 µl of glutaraldehyde solution (1%), which was then placed in the freezer (4 °C) overnight to aid the cross‐linking process. The working electrode was also immersed with 10 µl of a 3% weight‐based polyurethane^[^ ^22b^ ^]^ solution dissolved in dimethylformamide. Finally, the outermost layer of the working electrode was immersed with 10 µl of PVA/PEG in deionized water (PVA:PEG ratio, 8%:2% in wt.%).

### Electrochemical Characterization of the Microtube‐Based Biosensor

The biosensors were immersed in 200 µl of PBS solution containing 5 mm H_2_O_2_ to characterize the biosensors with different PANI layer thicknesses. The electrochemical impedance spectra were swept between 1 × 10^−2^ and 1 × 105 Hz, and the CV test was scanned between ‐1 and 1 V at a rate of 100 mV s^−1^.

The working electrode was used in amperometric experiments at room temperature with a constant voltage of ‐0.1 V (vs Ag/AgCl electrode) to assess the biosensor's sensing capabilities. The biosensor's capability to detect glucose was evaluated in PBS (1x PBS, pH 7.4) and simulated interstitial fluid. The biosensor was first placed in 200 µl of PBS for PBS detection, and 8 µl incremental drops of solutions with different glucose concentrations were added when the biosensor was stabilized.

Simulated skin interstitial fluid was generated based on the sodium salt of alginic acid. The composition of simulated interstitial fluid includes: 1.5% w/v sodium alginate matrix (providing structural biomimicry), 0.1 M KCl electrolyte solution (maintaining physiological ionic strength), 0.2 M CaCl_2_cross‐linking agent (enabling hydrogel formation). Granules of sodium alginate were combined with 0.1 M KCl to produce a 1.5% w/v viscous solution, which was then agitated at 45 °C for 4 h in an oven. The desired glucose concentration range was obtained by combining the mixture with various glucose powders. Next, the alginate solution was mixed with a 0.2 M calcium chloride (CaCl2) solution. The combined solution was then sealed in a parafilm and left at 4 °C overnight to produce the hydrogel. The microtubes were placed in hydrogels with various glucose concentrations to record the amperometric baseline responses for 50 s.

A selective study investigated how various electroactive compounds, including 2 mm lactate, 0.1 mm uric acid, 0.1 mm ascorbic acid, and 0.1 mm dopamine, affected glucose sensing (5 mm). Additionally, various insulin solutions (Insulin Aspart Injection, Novo Nordisk (China) Pharmaceutical Co., Ltd.) concentrations (100 U ml^−1^) on glucose sensing (5 mm) were examined to assess the inference of insulin. The amperometric test involved measuring the current–time curve after adding several analytes.

The sensing capacity to detect 5 mm glucose in a PBS buffer at pH 6.0, 6.5, 7.0, 7.5, and 8.0 was assessed in order to examine the stability of the biosensor under varied pH conditions. By detecting 5 mm glucose in a PBS buffer on a hot plate at various temperatures, the biosensor's sensing capacity was assessed to determine its stability at various temperatures. The current response to 5 mm glucose was monitored over four weeks to assess the storage stability of the sensor. When not in use, the sensor was stored in PBS containing 5 mm glucose at room temperature.

### Fabrication and Characterization of the Electroosmotic Micropump

Ti (20 nm), Au (200 nm), and Ag (200 nm) layers were sequentially sputtered on a glass fiber membrane (SB06, Shanghai KinBio Co., Ltd.). The glass fiber membrane was immersed into 1 m NaOH solution and oxidized for 60 s at 5 mA to form the Ag/Ag_2_O layer. Afterward, the glass fiber membrane was cut into 1.5 cm length and width to serve as the anode and cathode of the electroosmotic micropump. As the core element of the micropump, the PC membrane (2.5 cm in diameter, Global Life Sciences Solutions Operations UK Ltd.) was initially submerged for 3 h at room temperature in a 2 mg ml^−1^ dopamine hydrochloride solution (dissolved in 1x PBS at pH 8.5). The modified membrane was rinsed thoroughly with deionized water. The PDA‐modified membrane was then kept in 20 mg ml^−1^ NH2‐PEG‐NH2 (MW = 20 000) solution for 24 h at 37 °C (1x PBS, pH 8.5). The PDA/PEG‐modified membrane was subsequently incubated in 10 mg ml^−1^ BSA solution (dissolved in PBS at pH 7.4) for 24 h at room temperature.

Every 5 min, the insulin released from the micropump was collected to measure the flow rate, and its weight was calculated using a balance. Then, using the density of the insulin solution as a reference, the weight was converted to a volume to determine the flow rate. Three measurements of each flow rate were taken at a given potential. The flow rates for releasing insulin were measured every day to evaluate the stability of the electroosmotic micropump over 7 days, and the insulin solution was stored in the reservoir during that period. To measure the basal rate accuracy of the micropump, it was operated at 1 V for 72 h. The flow rate was measured for 90 min every 12 h, and during each 90‐min interval, the weight of the released insulin was recorded every 5 min. After prolonged use (4 days), the pores of the PC membrane remained intact (Figure S33, Supporting Information), indicating that insulin does not clog the nanoporous membrane.

### Design and Fabrication of a Wireless Miniaturized PCB

The OP AMP1 was used to determine the potential on the sensor's reference electrode, and the ADC was used to capture it. To output this potential to the in‐phase input terminal of the OP AMP2 through the DAC, the reference electrode voltage was added to the working voltage required by the biosensor (manual setting). The voltage applied to the sensor's working electrode was the reference electrode potential plus the predetermined working potential. By converting the current passing through the biosensor's working electrode to voltage, the OP AMP2 simultaneously achieved I‐V conversion (current to voltage). The ADC collected the transformed voltage. The BLE chip would then use the ampere formula to convert the voltage data gathered by the ADC into current values before sending them to the smartphone. The BLE chip also simultaneously calculated whether the current value exceeded the critical value (manual setting). The DAC would send the driving voltage (manual setting) to the micropump if the critical value was surpassed.

A lithium‐ion battery with a capacity of 1500 mAh at 3.7 V could power the PCB for 72 h continuously. The micropump and biosensor may get a constant voltage of 0.1 to 0.3 V and 0 to 10 V, respectively, from the PCB. Amperometric current measurements had an accuracy of 1 nA and a range of 0 to 10 µA.

### In Vivo Experiments of the Patch in Diabetic Rats

The Research Ethics Committee of Peking University First Hospital approved the in vivo experiments on diabetic rats (approval number: 2 021 112). Male 150–200‐g SD rats were provided by Beijing Charles River Biotechnology Co., Ltd. (License No.: SCXK(jing)2021‐0006). Rats were injected with streptozotocin (STZ, Sigma Aldrich Co., Ltd.), a drug specifically designed to injure pancreatic islet cells and cause diabetes. Prior to STZ injection, all the rats were fasted for 6 to 8 h and given only water. After dissolving the STZ powder in sodium citrate buffer (50 mm, pH 4.5) at a concentration of 32.5 mg mL^−1^, the rats were intraperitoneally injected with the STZ solution for 10 min at 65 mg of STZ per kilogram of rats. On the tenth day, after the rats had fasted for 6 to 8 h, blood was collected from the tail vein, and a commercial glucometer was used to measure the blood glucose levels. Rats were determined to be diabetic if their blood glucose level exceeded 8.3 mm.

Before the in vivo study of diabetic rats using the closed‐loop bioelectronic artificial pancreas patch, all rats were fasted for 6 to 8 h. The rats were then anesthetized with isoflurane using a small‐animal anesthesia machine (Beijing Zhongshi Dichuang Technology Development Co., Ltd.).

The artificial pancreas patch was mounted on the shaved and cleaned skin on the rat's back. The rat experiments were performed with patches based on the microtube arrays (height: 2.3 mm; outer diameter: 1.5 mm; inner diameter: 1.0 mm) and the dissolvable microneedle arrays (diameter: 0.9 mm; cylinder height: 2.5 mm; cone height: 0.5 mm). The device must be calibrated and stabilized for ≈30 min before the artificial pancreas patch was operated. During this period, insulin was not released. The closed‐loop blood glucose management algorithm was based on the simple on‐off algorithm. After starting the device, the sensor read the blood glucose value every 650 s for 50 s, and the final current point of the 50 s was used to establish the calibration curve and calculate the blood glucose. If the critical value (8.3 mm) was reached, the micropump was activated via the PCB to release insulin (100 U ml^−1^) at a continuous potential (1 V) for 10 min. The biosensor then measured the blood glucose level again. Blood glucose levels were monitored and controlled using the sensor and pump until they were within the normal range. Thereafter, only the blood glucose test was continued, and the insulin release was stopped. Throughout the sensing process, blood samples collected from the tail vein of each rat were tested using a commercial glucometer. The glucometer from Sibionics was only used in the green line shown in Figure 5h and other blood glucose results were measured by the glucometer from Sinocare.

### In Vivo Experimental Condition of Diabetic Pigs

The Research Ethics Committee of Peking University First Hospital also approved an in vivo trial on diabetic pigs (approval number: 2 022 097). All pigs were female, six months old, and weighed between 20 and 25 kg. They were purchased from Beijing Beiqijia Meile Farm under license number SCXK^[^ ^50^ ^]^2018‐0003. STZ was likewise used to induce diabetes in the pigs, and all pigs were fasted overnight and given only water before STZ injection. STZ powder at a concentration of 100 mg ml^−1^ was dissolved in sodium citrate buffer (50 mm, pH 4.5), and the pigs were then intravenously injected with the STZ solution for 10 min at 150 mg of STZ per kilogram of pig. On day 7, the pigs were fasted from the previous night, and their blood glucose levels were measured using a CGM device.^[^ ^42^ ^]^ If the blood glucose level exceeded 7.0 mm, the pig was successfully modeled as having STZ‐induced diabetes.

Before the in vivo testing of diabetic pigs, the area of the pig's back skin where the closed‐loop bioelectronic artificial pancreas patch was deployed was shaved and sterilized. During the experiment, each pig was fed 500 g of forage (≈300 g carbohydrates, Keao Xieli Feed Co., Ltd.) twice a day at ≈10 a.m. and 4 p.m. The endogenous insulin concentration of pigs was also measured after fasting overnight using the Pig Insulin ELISA kit, following the manufacturer's protocol (Wuhan Cusabio Biotechnology Co., LTD.).

The blood glucose levels of three pigs were measured under different conditions over 72 h, including no treatment, with the commercial closed‐loop system, and with the closed‐loop patch. The order of these conditions was randomized, and after each condition was measured, each pig would rest for a few days until its blood glucose returned to its original level.

### In Vivo Experiments with the Patch in Diabetic Pigs

The pig experiments were performed with patches based on the microtube arrays (height: 2.3 mm; outer diameter: 1.5 mm; inner diameter: 1.0 mm) and the dissolvable microneedle arrays (diameter: 0.9 mm; cylinder height: 2.5 mm; cone height: 0.5 mm). To avoid frequent insulin refilling, a larger insulin reservoir was used in vivo experiments in pigs, which was 2.25 cm in diameter, 7.5 mm in thickness, and could hold ≈2.98 ml insulin. The patch was then installed and secured to the skin on the pig's back. It took an hour before the artificial pancreas patch could stabilize the sensor and perform a closed‐loop operation. During the waiting period, the patch would not release insulin.

The hybrid closed‐loop control algorithm and simple on‐off control algorithm were designed for the patch. The hybrid closed‐loop control algorithm was shown in Figures S43 and S48 (Supporting Information). The glucose sensing process of the biosensor required 50 s, and the final current point from this 50 s‐interval was used to establish the calibration curve and calculate the blood glucose level. The sampling interval of the sensing current was ≈1.4 s. After 10 s, the PCB decided the pumping potential according to the glucose detection results, and powered the micropump to release insulin (100 U ml^−1^) at a constant potential for 180 s. Then, a 60 s period was waited for the insulin adsorption in the skin. The pumping time multiplied by the voltage every 120 min was limited to 15–21 V⋅min. A commercial CGM device was also attached to the pig's skin to monitor the blood glucose levels throughout the process, which reported the blood glucose change every 5 min. The biosensor was calibrated approximately every 12 h to adjust the calibration curve based on all currents measured by the biosensor and the blood glucose measured by the commercial CGM before each calibration.

The simple on‐off control algorithm was shown in Figure S30 (Supporting Information). The biosensor measured the blood glucose level once every 250 s, the sensing process needed 50 s, and the final current point of the 50 s was used to establish the calibration curve and calculate the blood glucose. When the blood glucose value exceeded a critical value (7.0 mm), the PCB powered the micropump to release insulin (100 U ml^−1^) at a constant potential (1.0 V) for 250 s. Sensing and pumping were performed alternately until the blood glucose level dropped below the critical value. A blood‐glucose test was then conducted while the release of insulin was stopped. The biosensor was calibrated approximately every 12 h based on the blood glucose measurements by the commercial CGM to adjust the threshold value for releasing insulin.

To measure the insulin Aspart adsorption rate of three pigs, the closed‐loop patch was applied in three pigs after fasting overnight to continuously release insulin for 10 min. After that, blood samples were collected from pigs at 0, 10, 20, 30, 40, 60, 90, 120, 180, 240, and 300 min. Blood glucose levels were also measured at the corresponding time by using the commercial glucometer (Sinocare). The pig's plasma insulin Aspart concentration was measured using the Insulin Aspart ELISA kit according to the manufacturer's protocol (Quanzhou Blueprint Biotechnology Co., LTD.).

To measure the influence of the external pressure on the biosensor, different qualities of weights (100 g, 200 g, 500 g, and 1 kg) were applied vertically on the biosensor, and the sensing current was measured on the pig.

### In Vivo Experiments with the Commercial Systems in Diabetic Pigs

To compare the performance of the developed closed‐loop system, a commercial closed‐loop system was applied to diabetic pigs. The commercial system combines a Dana Diabecare R insulin pump (Sooil Development Co., Ltd., Korea), a CGM,^[^ ^42^ ^]^ and a AndroidAPS control algorithm. The insulin pump filled with 3 ml insulin (100 U ml^−1^) and connected with the disposable sterilized infusion set, and a commercial CGM were attached to the pig's back skin and fixed with a cohesive bandage. The distance between the infusion set and the commercial CGM was ≈10 cm. It took an hour for the CGM to be stabilized, and during the waiting period, no insulin was released from the insulin pump.

The Android APS control algorithm utilized within the commercial closed‐loop system is an open‐source control mechanism thoughtfully designed to forecast glucose values within a specific target range. This advanced algorithm relies on blood glucose prediction curves, formulated from factors such as injected insulin, carbohydrate consumption, unannounced meals, and zero‐temporary basal insulin. Three individual insulin dosing parameters were needed by this algorithm, including the basal insulin rate, the ISF and the ICR. The basal insulin rate was optimized based on the weight and severity of diabetes in pigs, with insulin injection rates studied in the range of 0.6–1 U kg^−1^ weight. ISF was determined by injecting 4 or 5 U of insulin after an overnight fast and observing the change in blood glucose over 90 min, then calculating the change in glucose per unit of insulin. The determination of ICR was performed by injecting 8 or 10 units of insulin into a pig, along with feeding 500 g of feed (≈300 g of carbohydrates), observing the change in blood glucose within 2 h to be within 3.3 mm, and then dividing the amount of carbohydrates by the amount of insulin. With the AndroidAPS control algorithm and the optimized parameters of basal insulin rate, ISF, and ICR entered into the system, the commercial closed‐loop system could intelligently manage glucose levels. An additional insulin injection was administered ≈10 min before feeding, and its amount was determined by AndroidAPS. This calculation was based on the current blood glucose level, the previous insulin dose delivered by the pump, the parameters of basal insulin rate, ISF, and ICR, as well as the carbohydrate intake entered.

## Conflict of Interest

The authors declare no conflict of interest.

## Supporting information



Supporting Information

## Data Availability

The data that support the findings of this study are available from the corresponding author upon reasonable request.
