# Supplementary material for: Closed‐Loop Bioelectronic Artificial Pancreas Patch for Continuous Monitoring and Regulation of Blood Glucose in Diabetic Rats and Pigs
Source: Adv Sci (Weinh). 2025 Sep 16;12(44):e03536. doi: 10.1002/advs.202503536 (PMC12667471; doi:10.1002/advs.202503536)
Supplement: Supplementary file 1 — Supporting Information [file ADVS-12-e03536-s001.docx]

Supporting Information

Closed-Loop Bioelectronic Artificial Pancreas Patch for Continuous Monitoring and Regulation of Blood Glucose in Diabetic Rats and Pigs

Yiqun Liu^+^, Changxi Zhang^+^, Lingyi Xu, Yuan Ma, Yuanyuan Ma, Ying Chen, Difei Lu, Le Ye, Li Yang, Yue Cui*

Y. Liu, C. Zhang, Y. Cui

School of Materials Science and Engineering, Peking University, Beijing 100871, P.R. China

E-mail: [ycui@pku.edu.cn](mailto:ycui@pku.edu.cn)

L. Xu, Y. Chen, Y. Ma, L. Yang

Renal Division, Peking University First Hospital; Peking University Institute of Nephrology; Key Laboratory of Renal Disease, Ministry of Health of China; Key Laboratory of Chronic Kidney Disease Prevention and Treatment (Peking University), Ministry of Education, Beijing 100034, P. R. China

Y.Y. Ma

Laboratory Animal Center, Peking University First Hospital, Beijing 100034, P.R. China

1. Lu

Department of Endocrinology, Peking University First Hospital, Beijing 100034, P.R. China

1. Ye

School of Integrated Circuits, Peking University, Beijing 100871, P.R. China

^+^These authors contribute equally

**Contents**

**Manufacturing cost analysis of the entire system**

**Supplementary Figures**

Supplementary Figure 1. Back photograph of the printed circuit board (PCB).

Supplementary Figure 2. Photograph of a closed-loop system, including a sensor, a micropump, a PCB, and a battery.

Supplementary Figure 3. Photographs of the hollow 3D-printed microneedle.

Supplementary Figure 4. SEM images and EDS mapping analysis of the electrode.

Supplementary Figure 5. Photographs of the electrodeposition tank and the electrode.

Supplementary Figure 6. SEM images of different microtubes and dissolvable microneedles.

Supplementary Figure 7. Dissolution process of the microneedle in PBS.

Supplementary Figure 8. Dissolution process of the microneedle in stimulated interstitial fluid.

Supplementary Figure 9. Characterization of the mechanical performance of the microneedles and microtubes.

Supplementary Figure 10. Bode plots of the sensors in PBS containing 5 mM H_2_O_2_ with various PANI deposition currents.

Supplementary Figure 11. Potential-versus-time curves for electro-depositing PANI layer at different currents.

Supplementary Figure 12. CV curves of biosensors with various PANI deposition times in PBS containing 5 mM H_2_O_2_ (deposition current: 0.1 mA).

Supplementary Figure 13. CV curves for depositing and stabilizing PB on the working electrode.

Supplementary Figure 14. SEM images of different layers on the working electrode.

Supplementary Figure 15. EDS point analysis of the electrode.

Supplementary Figure 16. CV curves of the biosensor in PBS containing 5 mM glucose at different scan rates.

Supplementary Figure 17. Storage stability of the biosensors in PBS containing 5 mM glucose at room temperature with only enzyme and glutaraldehyde (GA) layers, and without one of the PANI, TPU, PVA/PEG layers (n=3).

Supplementary Figure 18. Consistency of the biosensor for 50 continuous measurements of 5 mM glucose in PBS.

Supplementary Figure 19. Stability of the Ag/AgCl reference electrode of microtube in 0.1 M and 1 M NaCl solution.

Supplementary Figure 20. Long-term stability of the Ag/AgCl reference electrode of microtube in the simulated interstitial fluid.

Supplementary Figure 21. Electrode potentials of the Ag/Ag2O pump and Pt pump.

Supplementary Figure 22. SEM image and EDS mapping analysis of the original Ag glass fiber electrode.

Supplementary Figure 23. Photographs of the Ag/Ag_2_O glass fiber electrodes with different oxidation times.

Supplementary Figure 24. Relationship between the oxidation times of Ag/Ag_2_O glass fiber electrodes and the currents of the micropump at different potentials.

Supplementary Figure 25. Current-versus-time curves of the micropump operated under 1 V for 12 hours.

Supplementary Figure 26. Current-versus-time curve of the closed-loop system during the 5-minute cycling process.

Supplementary Figure 27. Insulin flow from a micropump connected to a hollow 3D printed microneedle array at 1 V voltage.

Supplementary Figure 28. Power/flow rate of the micropump for releasing insulin at different potentials.

Supplementary Figure 29. Voltage-versus-time curve of the lithium battery in the closed-loop system (battery capacity: 1500 mAh).

Supplementary Figure 30. Characterization of the electroosmotic micropump with an aluminum mesh as the anode and a stainless-steel mesh as the cathode.

Supplementary Figure 31. Basal rate accuracy of the micropump under 1 V over 72 h.

Supplementary Figure 32. Relative flow rate of the electroosmotic pump changes over time when operating at a constant voltage of 1V for an extended period.

Supplementary Figure 33. SEM image of the PC membrane after 4days of use.

Supplementary Figure 34. Optical images of hematoxylin-eosin (HE) staining of Bama pig and SD rat.

Supplementary Figure 35. Pressure marks left on the skin of the back of a rat after wearing the system.

Supplementary Figure 36. Optical images of HE-stained pierced rat back-skin sections after use of the patch.

Supplementary Figure 37. Operation model of the closed-loop system for managing diabetic rats’ blood glucose.

Supplementary Figure 38. Blood glucose levels in SD rats over time after manual injection of insulin stored in the reservoir for 7 days and 0 days.

Supplementary Figure 39. Rat skin irritation test for assessing *in-vivo* biocompatibility of the sensor.

Supplementary Figure 40. Optical mages of hematoxylin-eosin (HE) staining of skin after wearing microneedles (day1, 3, 5, 7).

Supplementary Figure 41. Optical images of hematoxylin-eosin (HE) staining of important organs (heart, liver, spleen, lung, and kidney) before and after wearing soluble microneedles.

Supplementary Figure 42. Performance of the commercial system in three pigs with different basal insulin rates.

Supplementary Figure 43. Hybrid closed-loop control algorithm for the system.

Supplementary Figure 44. On-off control algorithm for the system.

Supplementary Figure 45. Mobile application developed for system control and data display.

Supplementary Figure 46. Voltage of the insulin micropump at different times.

Supplementary Figure 47. Voltage of the insulin micropump at different times for the third pig.

Supplementary Figure 48. Distinct day-night hybrid closed-loop control algorithm for the system.

Supplementary Figure 49. Performance of the closed-loop system with the hybrid control algorithm applied to the third pig.

Supplementary Figure 50. Performance of the closed-loop system with the distinct day-night hybrid control algorithm applied to the second pig.

Supplementary Figure 51. Performance of the closed-loop system with the on-off control algorithm applied to the first and second pig.

Supplementary Figure 52. Clarke error grid analysis.

Supplementary Figure 53. Time lag between the pig’s blood glucose change measured by the closed-loop system and the current measured by only the biosensor.

Supplementary Figure 54. Time lag between the pig’s blood glucose change measured by the CGM and the current measured by the biosensor.

Supplementary Figure 55. Insulin adsorption pharmacokinetics and pharmacodynamics profiles in three pigs following a 10-min insulin release with the closed-loop system.

Supplementary Figure 56. Current measured by the biosensor on the pig with different continuous vertical pressures.

Supplementary Figure 57. Current measured by the biosensor on the pig with different instantaneous vertical pressures.

**Supplementary Tables**

Supplementary Table 1. Insulin sensitivity factor (ISF) and insulin-to-carbohydrate ratio (ICR) of pigs in three days.

Supplementary Table 2. Comparison of this work and other commercial closed-loop diabetes systems.

Supplementary Table 3. Comparison of this work and other closed-loop diabetes systems in academic publications.

**Manufacturing cost analysis of the entire system**

According to the annual reports published by Medtronic, Dexcom, and Tandem Diabetes Care,^[1]^ the manufacturing cost mainly includes the materials and labor costs for fabricating, assembling, and testing the device.

The materials cost of each component in our system is estimated to be less than $1 USD for the sensor structure, less than $1 USD for the sensing electrodes, less than $1 USD for the pump electrodes, less than $1 USD for the polycarbonate film, and less than $5 USD for the PCB. The total materials cost of the entire system is approximately about or less than $10 USD.

Labor costs mainly consist of salaries for personnel in each fabrication process, including personnel for fabricating the dissolvable microneedle, the 3D printing process of the microtube, the sputtering process of the sensing and pump electrodes, the electrochemical deposition process on the sensing electrode, the enzyme immobilization process on the sensing electrode, the modification process of the polycarbonate film, the fabrication process of the PCB, the final assembly, and testing of the entire system. For mass production, assuming an annual output of 1 million devices, personnel would mainly operate machines to automate production, with an estimated labor cost of about $100,0000 USD. Therefore, the average labor cost for a single device is estimated to be about $1 USD.

In summary, the total manufacturing cost of the system is estimated to be about $10 USD, and the entire system can be disposable. Additionally, if the PCB is reusable instead of disposable and only the biosensors and micropump are disposable, the total manufacturing cost of the system could be decreased to about $5 USD.


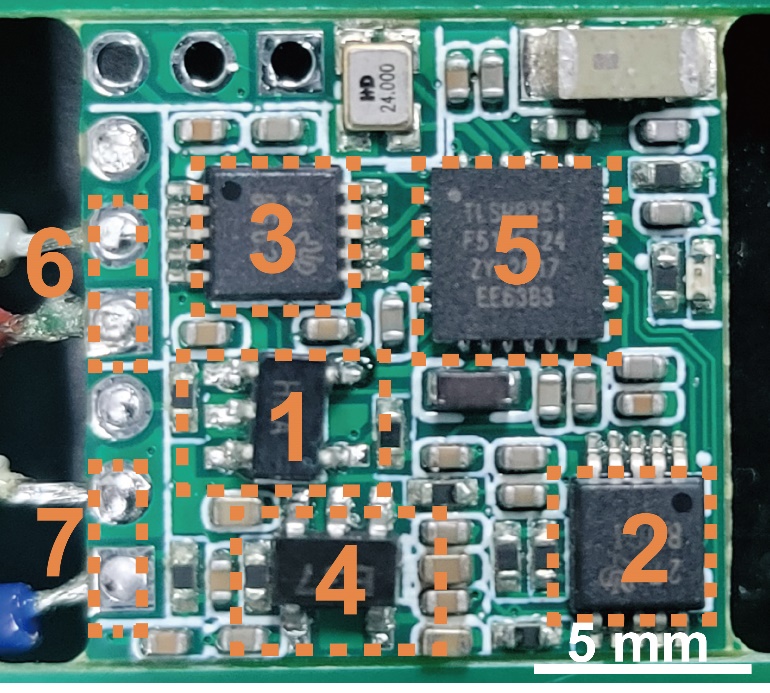


**Supplementary Figure 1. Back photograph of the printed circuit board (PCB).** (1: OP AMP1 (operational amplifier 1), 2: ADC (analog to digital converter), 3: DAC (digital to analog converter), 4: OP AMP2 (operational amplifier 2), 5: BLE chip (Bluetooth chip), 6: Sensor port, 7: Pump port).


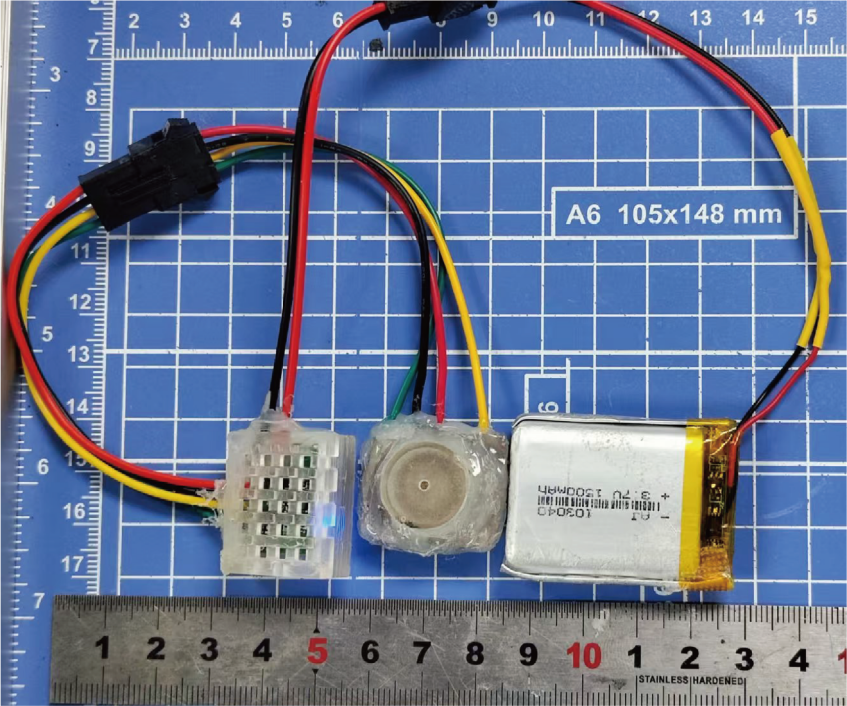


**Supplementary Figure 2. Photograph of a closed-loop system, including a sensor, a micropump, a PCB, and a battery.**


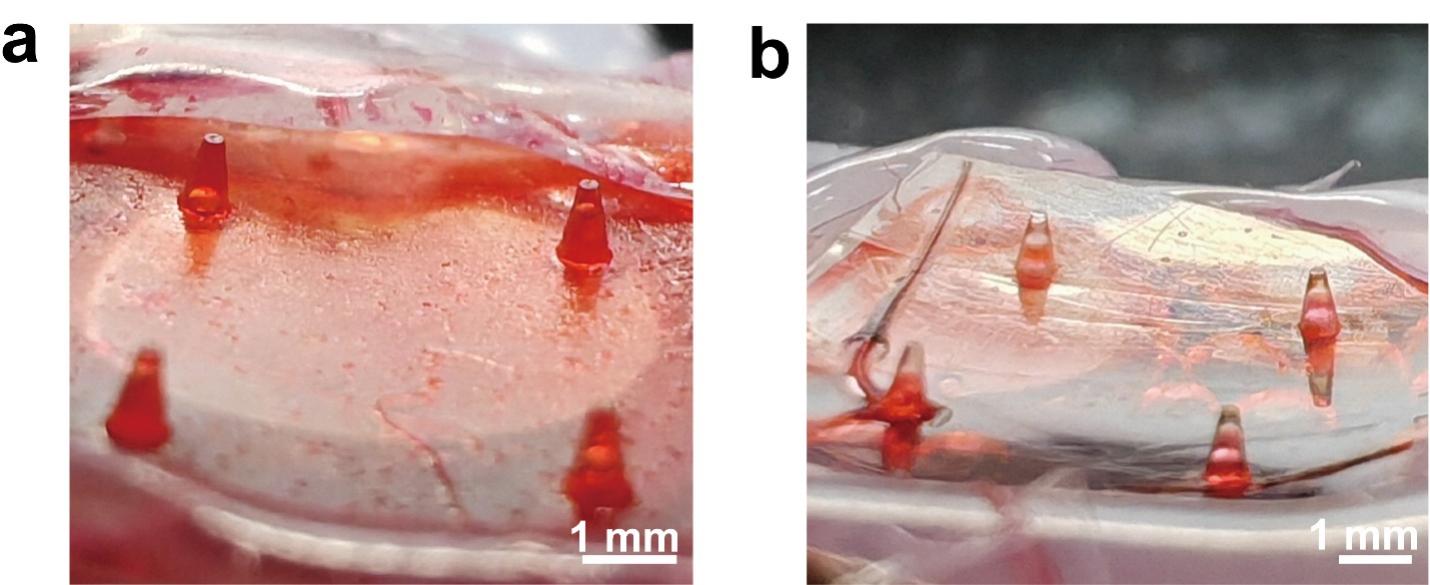


Supplementary Figure 3. Photographs of the hollow 3D-printed microneedle. a, Microneedle filled with red ink liquid. b, The tip of the hollow microneedle blocked by bubbles. The hollow microneedle was 1.0 mm in height, 0.6 mm in base outer diameter, 0.4 mm in base inner diameter, 0.2 mm in tip outer diameter, and 0.1 mm in tip inner diameter. The distance between two microneedles was 4 mm.


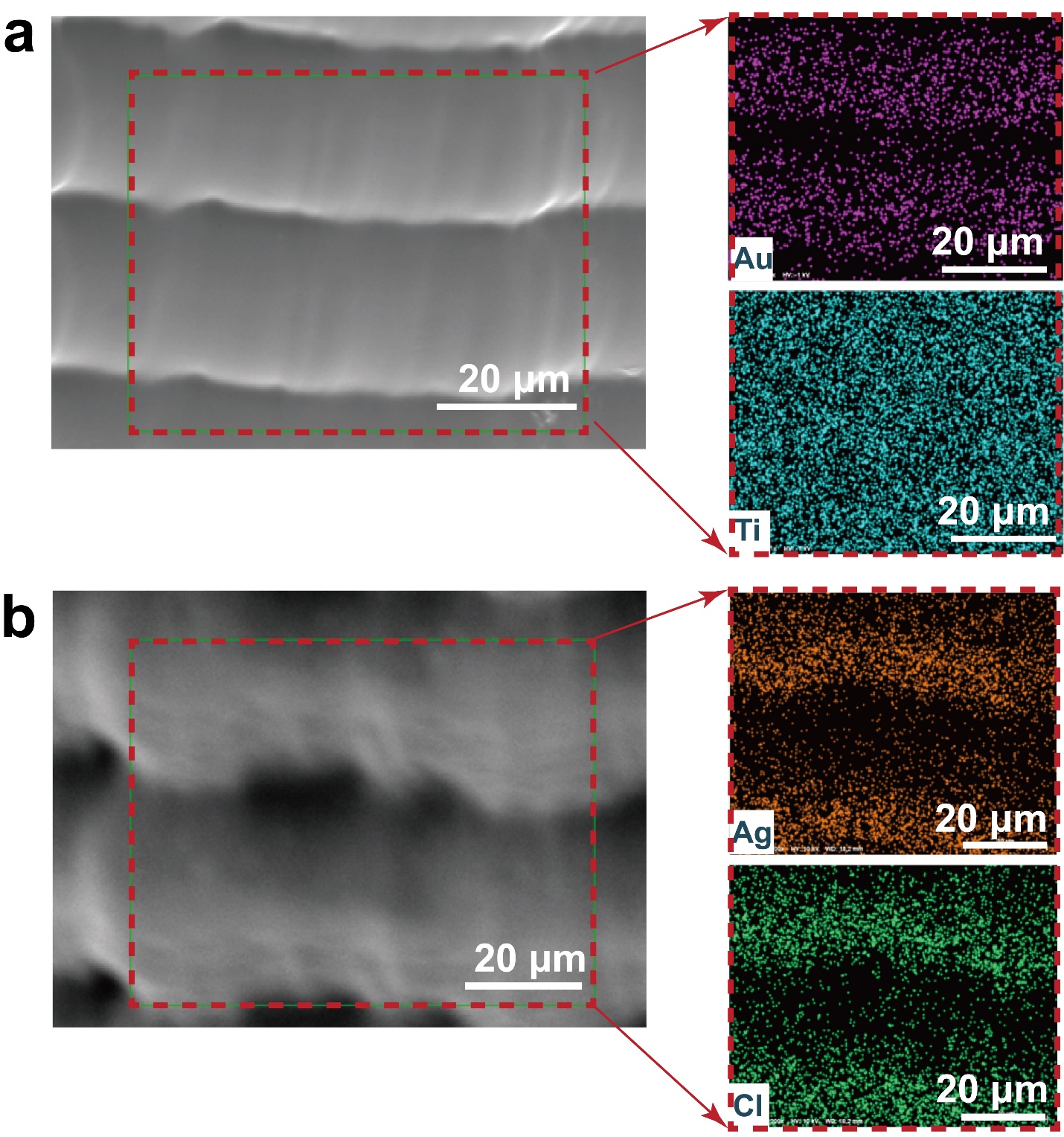


Supplementary Figure 4. SEM images and EDS mapping analysis of the electrode. a, The Au electrode. b, The Ag/AgCl electrode.


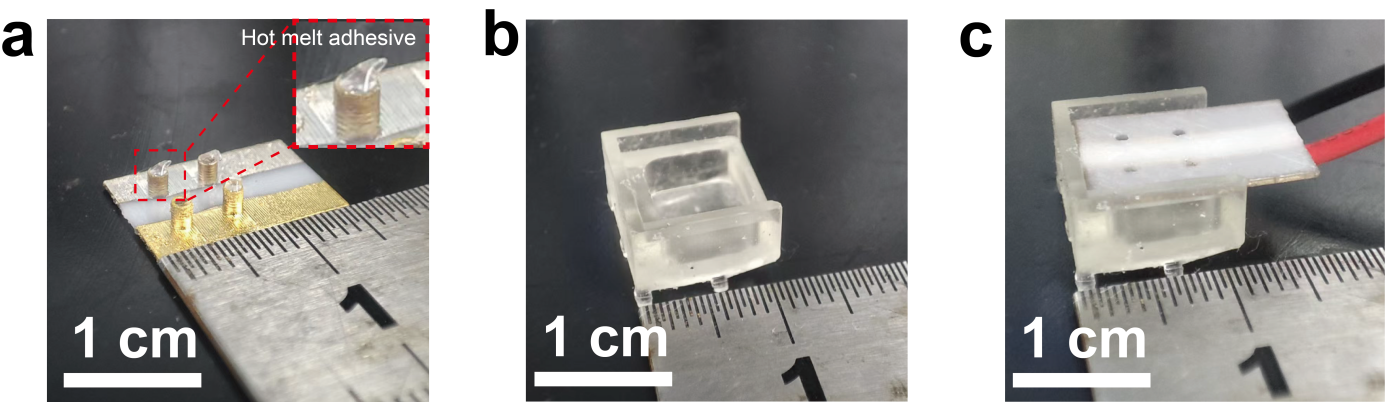


Supplementary Figure 5. Photographs of the electrodeposition tank and the electrode. a, The microtube sealed. b, The electrodeposition tank. c, The inverted microtubules on the electrodeposition tank.


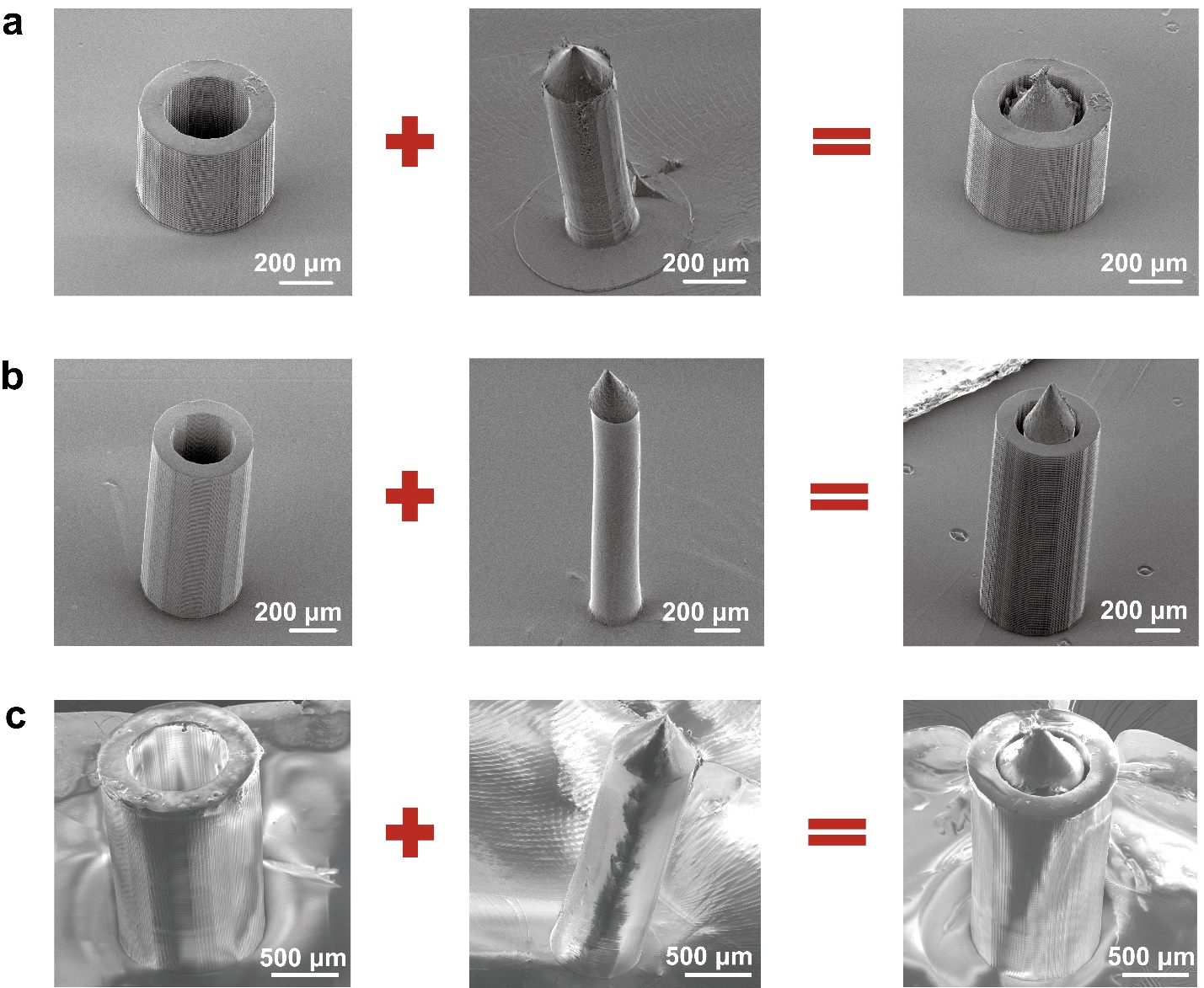


Supplementary Figure 6. SEM images of different microtubes and dissolvable microneedles. a, The dimensions of a microtube were 0.6 mm in height, 0.6 mm in outer diameter, and 0.4 mm in inner diameter. The dimensions of a dissolvable microneedle were 0.3 mm in diameter, 0.8 mm in cylinder height, and 0.2 mm in top cone height. b, The dimensions of a microtube were 1.5 mm in height, 0.6 mm in outer diameter, and 0.4 mm in inner diameter. The dimensions of a dissolvable microneedle were 0.35 mm in diameter, 1.7 mm in cylinder height, and 0.2 mm in top cone height. c, The dimensions of a microtube were 23 mm in height, 15 mm in outer diameter, and 10 mm in inner diameter. The dimensions of a dissolvable microneedle were 0.9 mm in diameter, 2.5 mm in cylinder height, and 0.5 mm in top cone height.


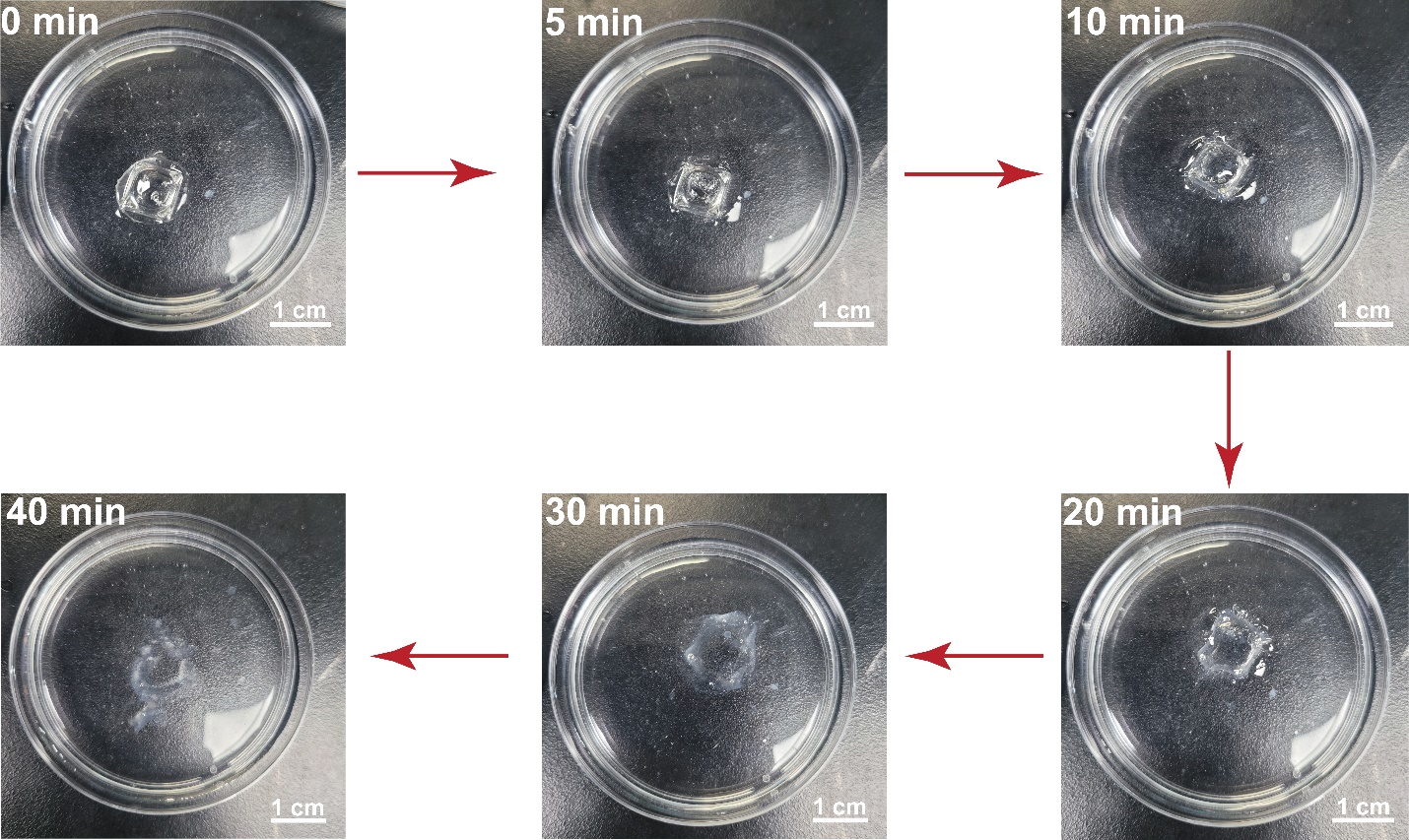


Supplementary Figure 7. Dissolution process of the microneedle in PBS.


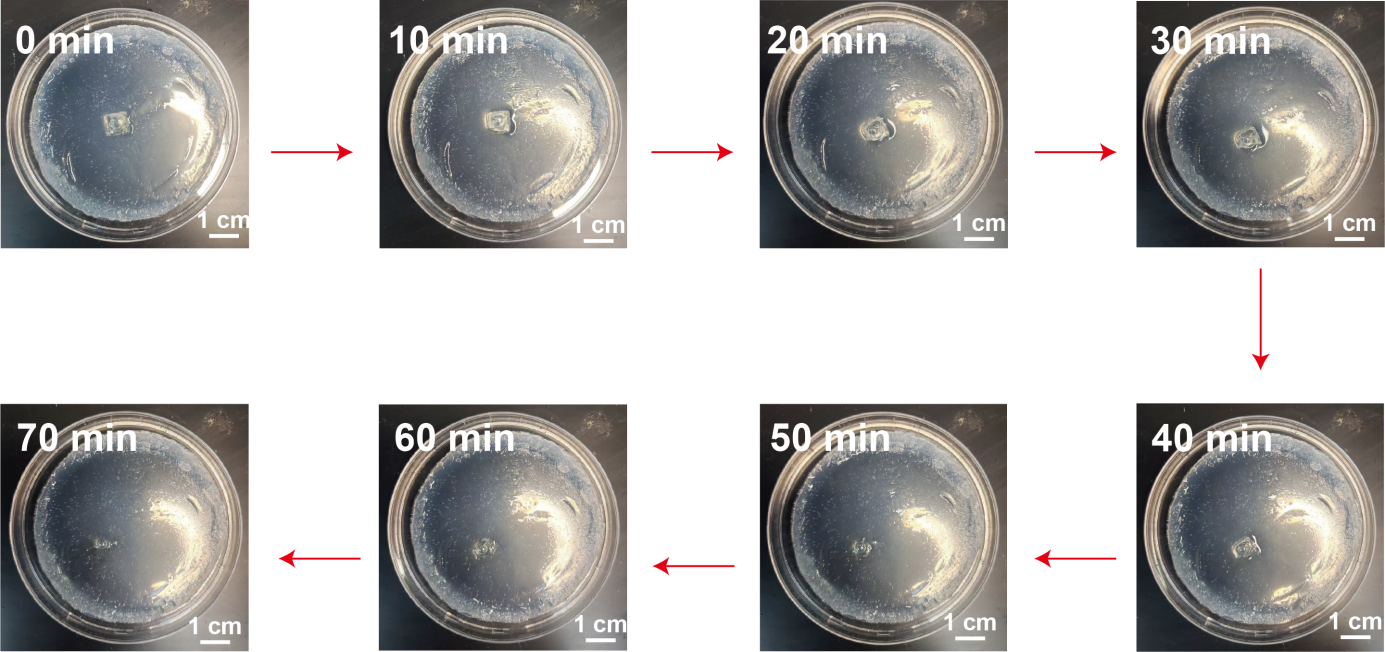


Supplementary Figure 8. Dissolution process of the microneedle in stimulated interstitial fluid.


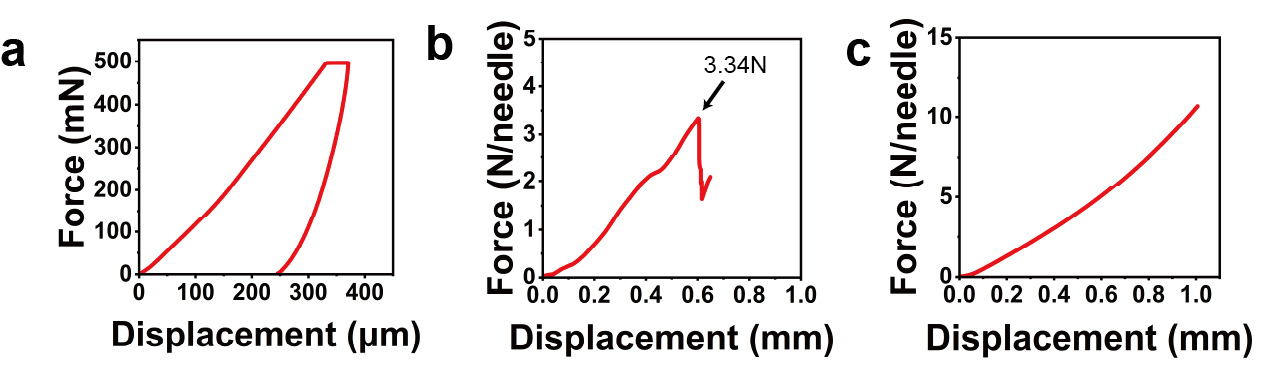


Supplementary Figure 9. Characterization of the mechanical performance of the microneedles and microtubes. a, The load–displacement curve on a microneedle by an in situ nanomechanical test system. b, Compression test on the microneedle array by a universal material testing machine. c, Compression test on the microtube array by a universal material testing machine.

The maximum compressive stress of the dissolvable microneedles is 3.34N/needle. The 3D-printed resin used for the microneedles has high strength, and its tubular structure provides excellent compressive resistance, showing no fracture under 1mm of compressive deformation.


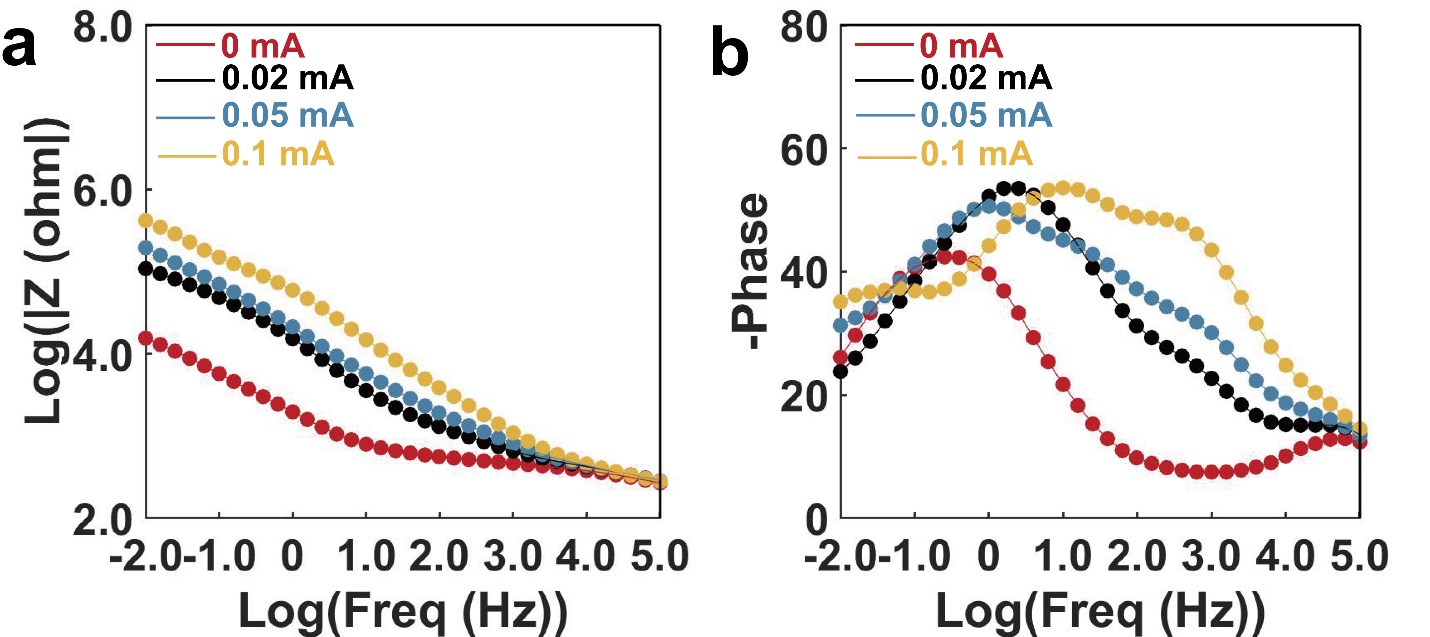


**Supplementary Figure 10. Bode plots of the sensors in PBS containing 5 mM H_2_O_2_ with various PANI deposition currents.** **a,** Bode plot of impedance versus log(frequency). **b,** Bode plot of phase versus log(frequency).


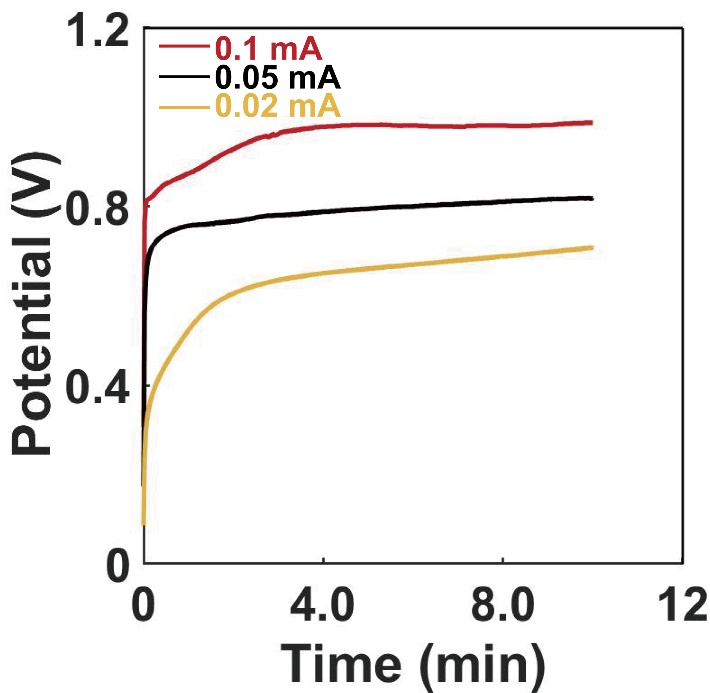


**Supplementary Figure 11. Potential-versus-time curves for electro-depositing PANI layer at different currents.**


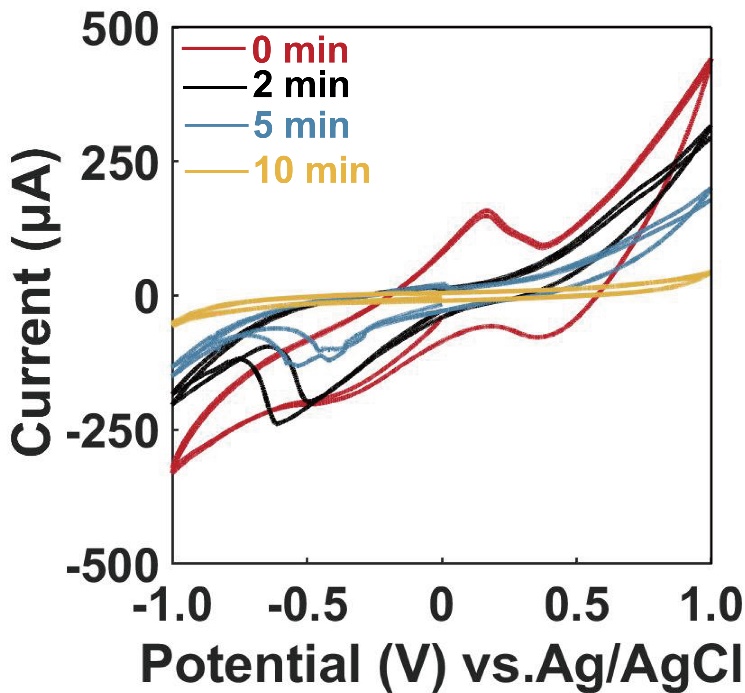


**Supplementary Figure 12. CV curves of biosensors with various PANI deposition times in PBS containing 5 mM H_2_O_2_ (deposition current: 0.1 mA).**


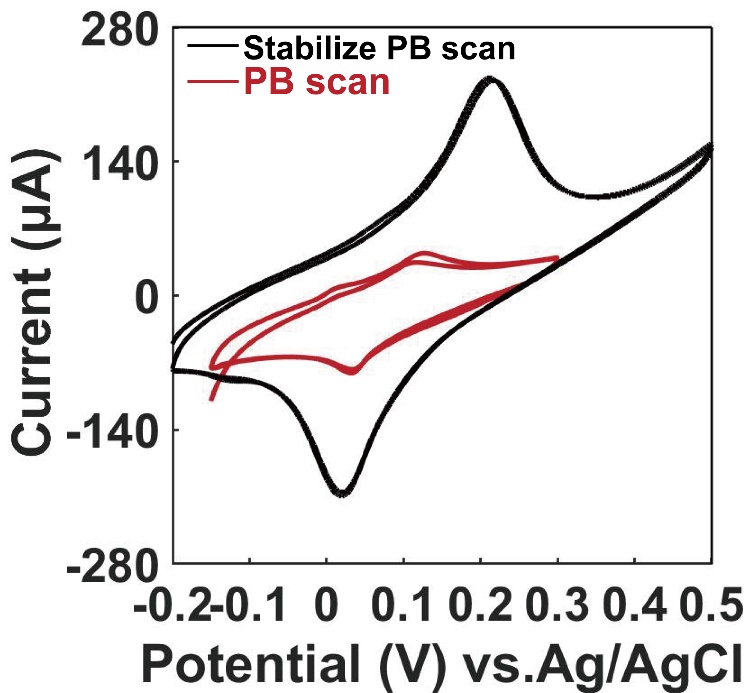


Supplementary Figure 13. CV curves for depositing and stabilizing PB on the working electrode.


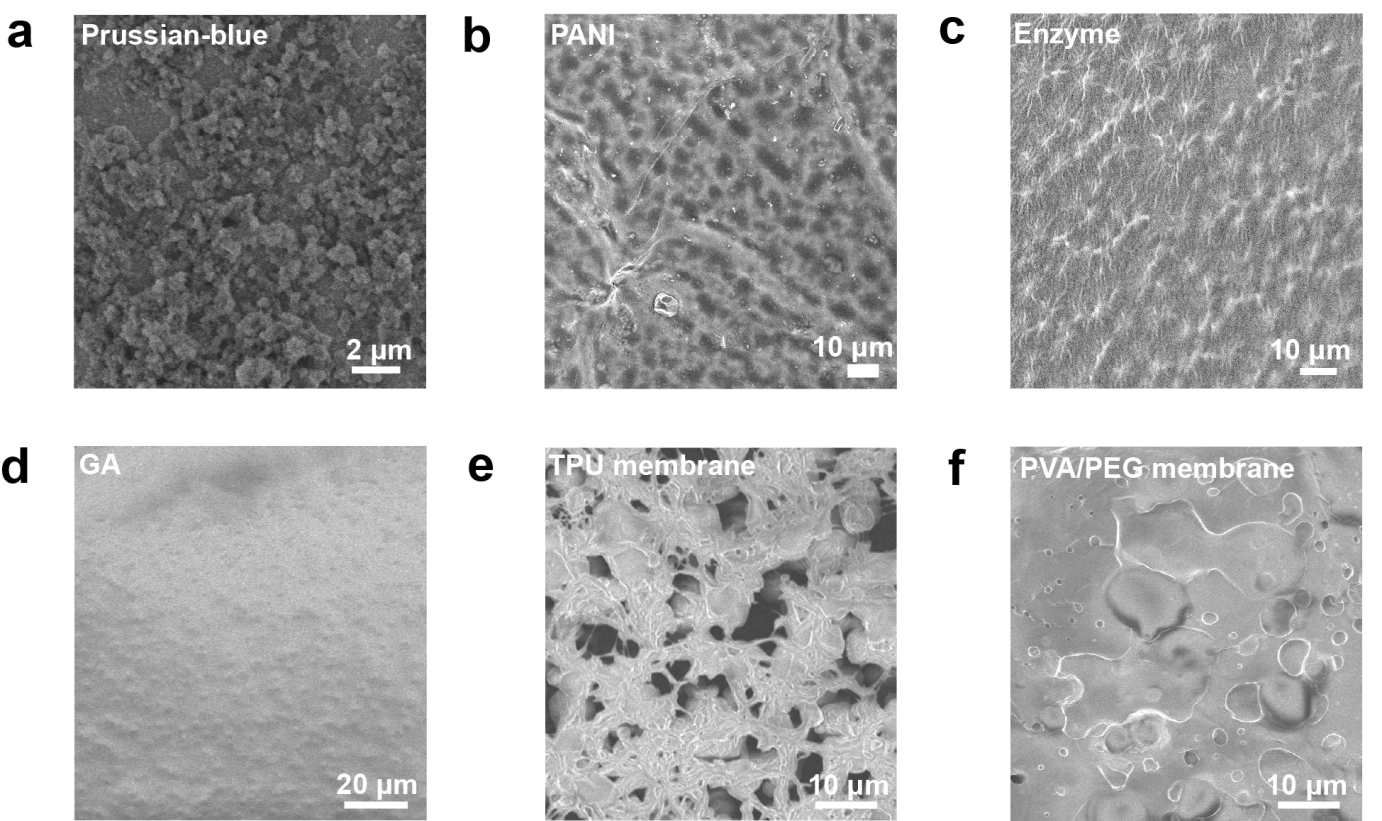


**Supplementary Figure 14. SEM images of different layers on the working electrode.** **a,** Prussian blue layer. **b,** PANI layer. **c,** Enzyme layer. **d,** glutaraldehyde (GA) layer. **e,** TPU membrane. **f,** PVA/PEG membrane.


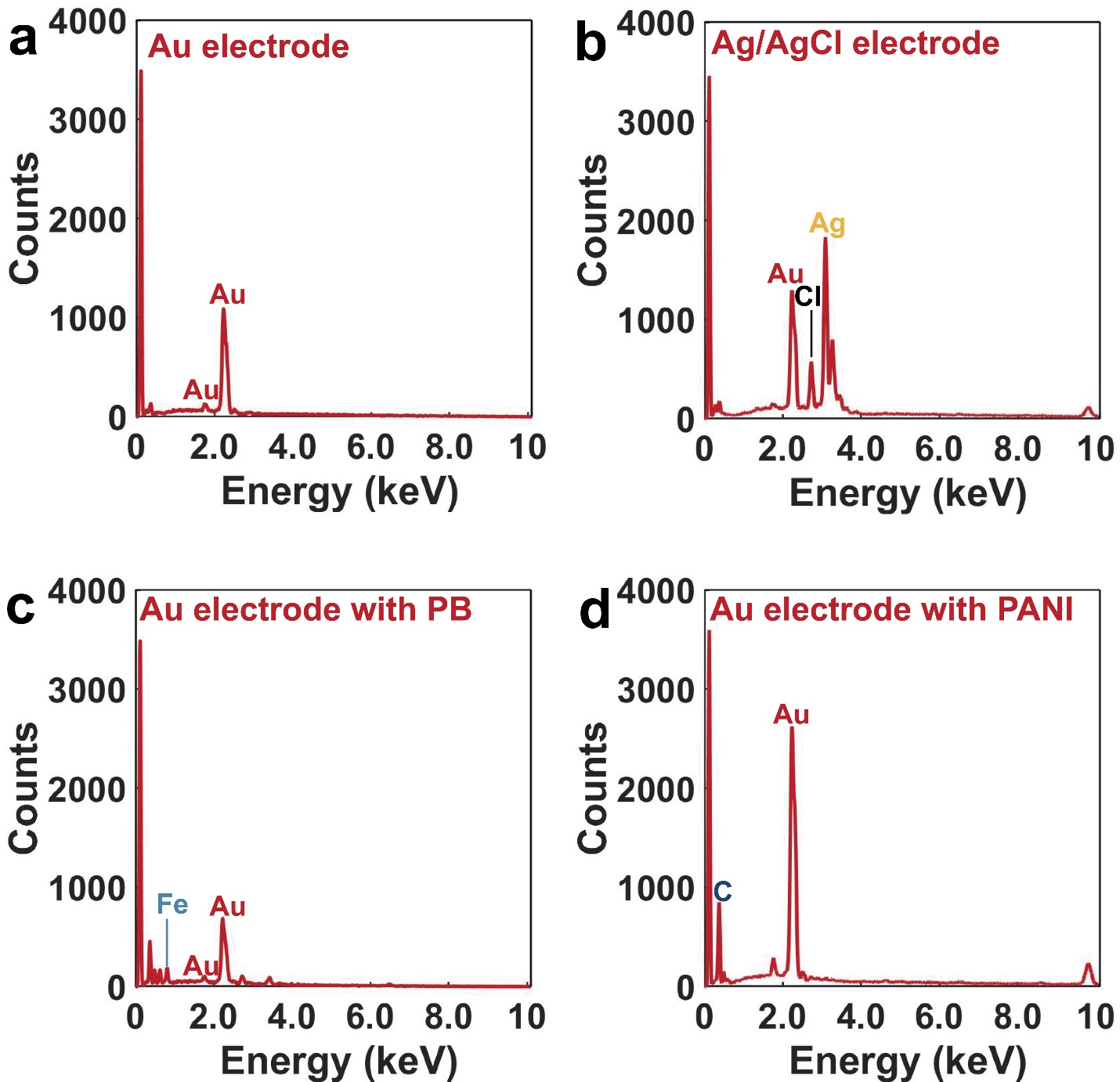


Supplementary Figure 15. EDS point analysis of the electrode. a, The original Au electrode. b, Ag/AgCl electrode. c, Au electrode with Prussian blue (PB) deposition. d, Au electrode with polyaniline (PANI) deposition.


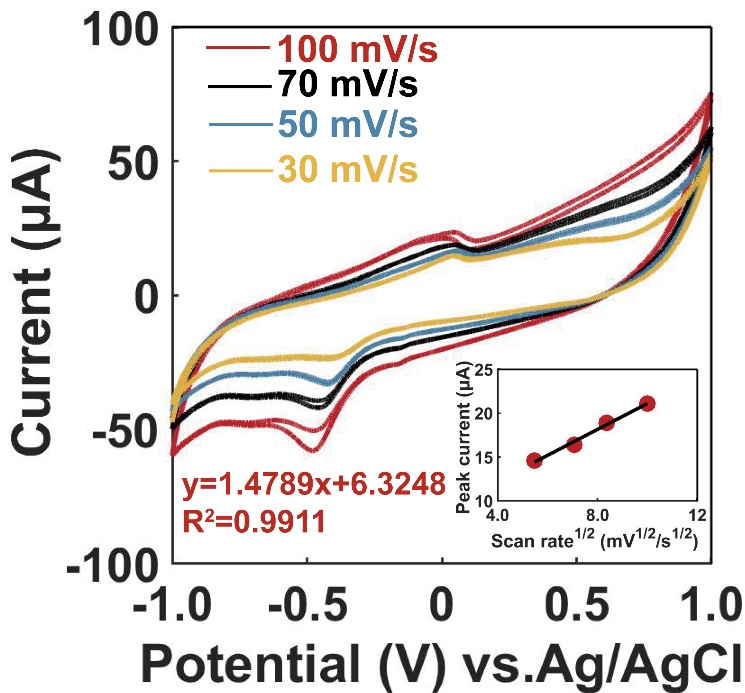


Supplementary Figure 16. CV curves of the biosensor in PBS containing 5 mM glucose at different scan rates.


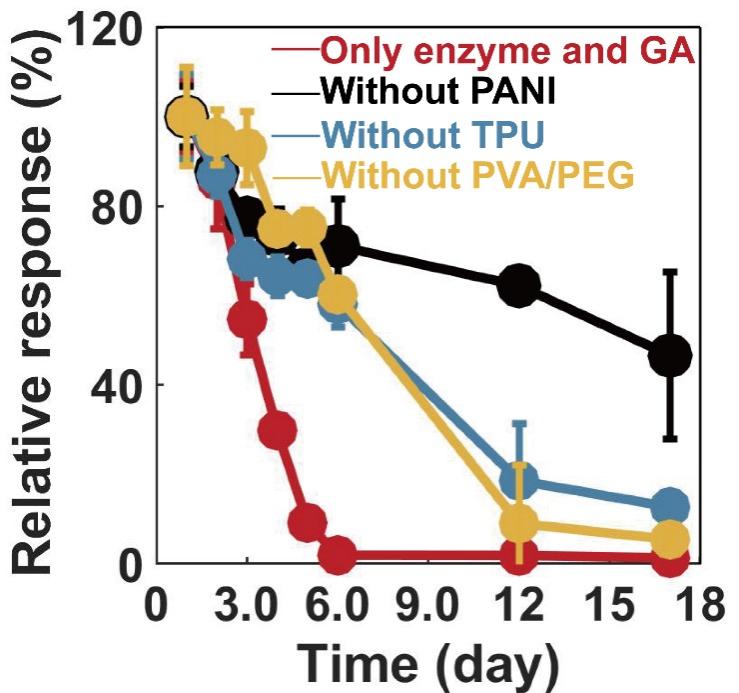


**Supplementary Figure 17. Storage stability of the biosensors in PBS containing 5 mM glucose at room temperature with only enzyme and glutaraldehyde (GA) layers, and without one of the PANI, TPU, PVA/PEG layers (n=3).**

When only enzyme and GA were present, the relative response dropped to 0 by the sixth day. This is because, without the encapsulation of outer polymers, the enzyme is highly susceptible to inactivation due to changes in environmental humidity and temperature. PANI functions by forming a cross-linked network structure on the electrode surface, and its nanostructure provides sufficient active surfaces for subsequent enzyme immobilization, thereby enhancing enzyme stability. Therefore, without the PANI layer, the relative response decreased to 42% after 18 days. The TPU membrane acts as a barrier to prevent enzyme leakage and regulate the diffusion of glucose to the working electrode surface. Hence, without the TPU layer, the relative response decreased to 19% after 18 days. Polyethylene glycol (PEG) is a biocompatible matrix material, and studies have shown that when PVA and PEG are combined at specific mass and component ratios, the resulting hydrogel exhibits excellent mechanical stability. As the outermost membrane, it protects the enzyme from hydrophobic external interfaces and prevents soluble enzyme inactivation. Consequently, without the PVA/PEG layer, the relative response decreased to 7% after 18 days.


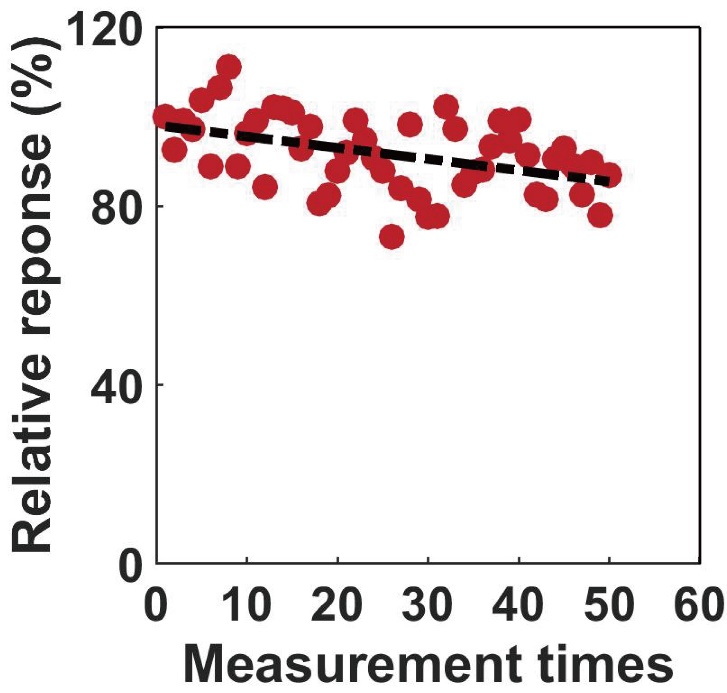


Supplementary Figure 18. Consistency of the biosensor for 50 continuous measurements of 5 mM glucose in PBS.


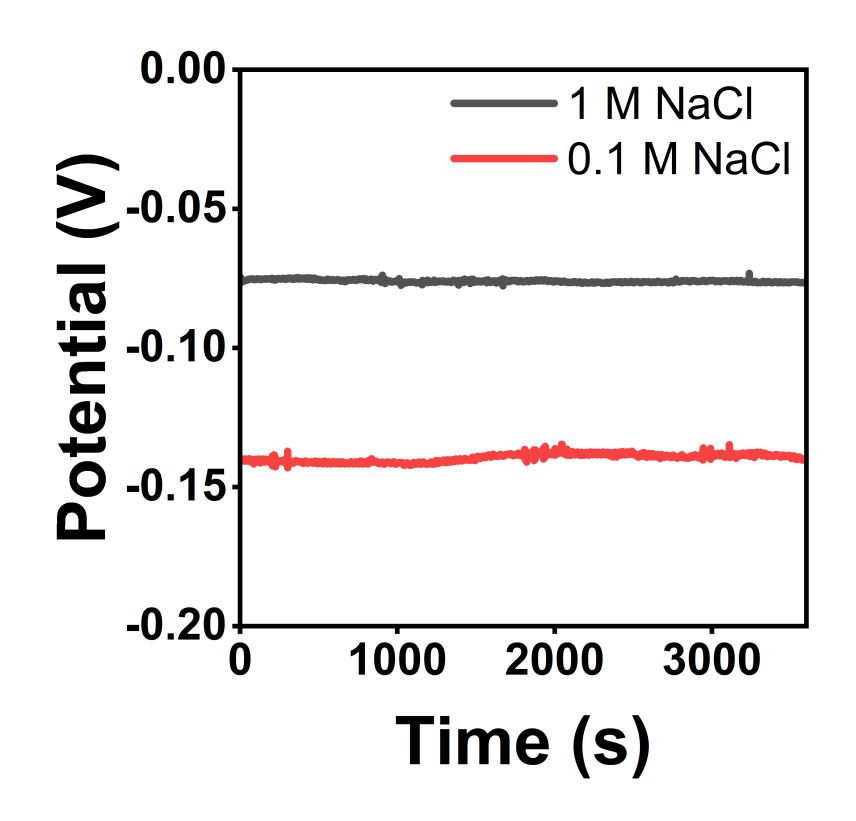


**Supplementary Figure 19. Stability of the Ag/AgCl reference electrode of microtube in 0.1 M and 1 M NaCl solution.**


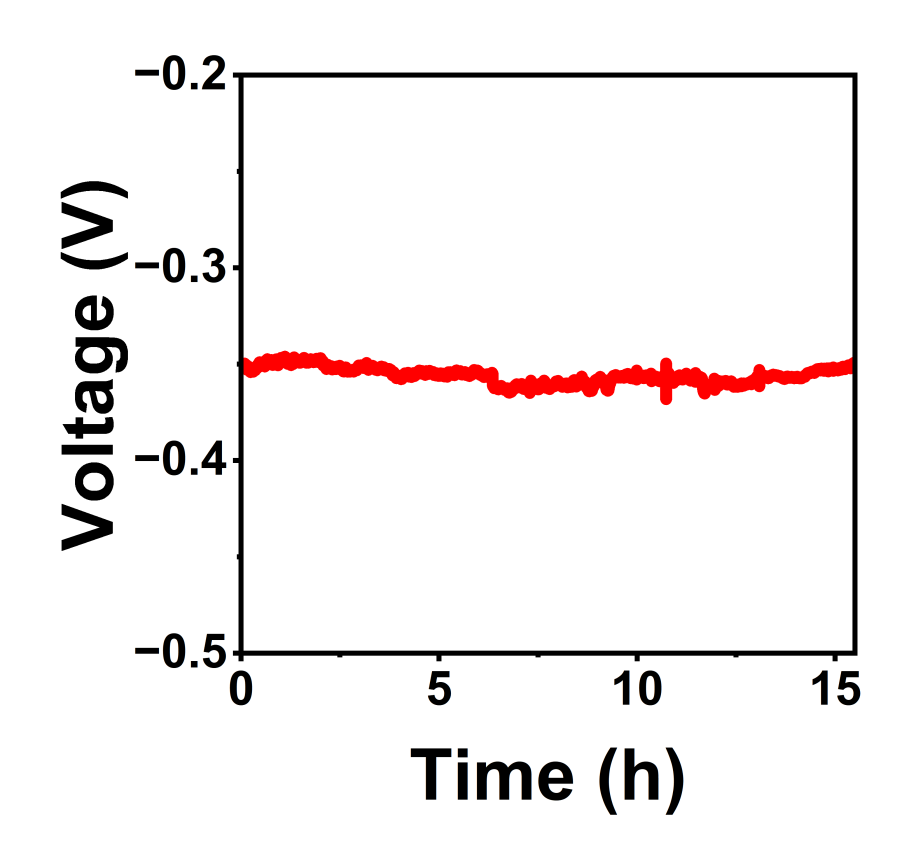


Supplementary Figure 20. Long-term stability of the Ag/AgCl reference electrode of microtube in the simulated interstitial fluid. The composition of simulated interstitial fluid includes: 1.5% w/v sodium alginate matrix, 0.1 M KCl electrolyte solution, 0.2 M CaCl₂ cross-linking agent.

**
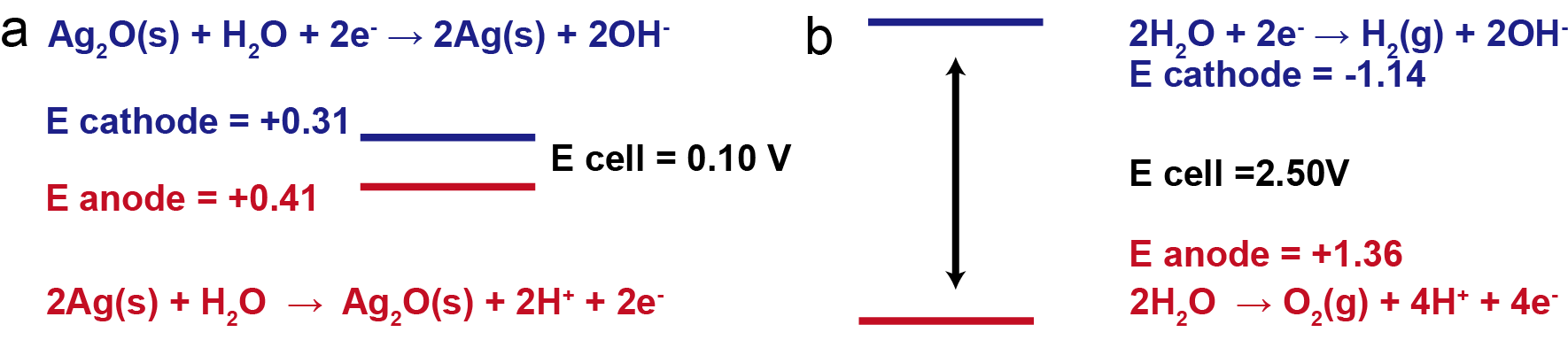
**

**Supplementary Figure 21. Electrode potentials of the Ag/Ag2O pump and Pt pump. a,** Electrode potentials of the Ag/Ag2O pump. **b,** Electrode potentials of the Pt pump


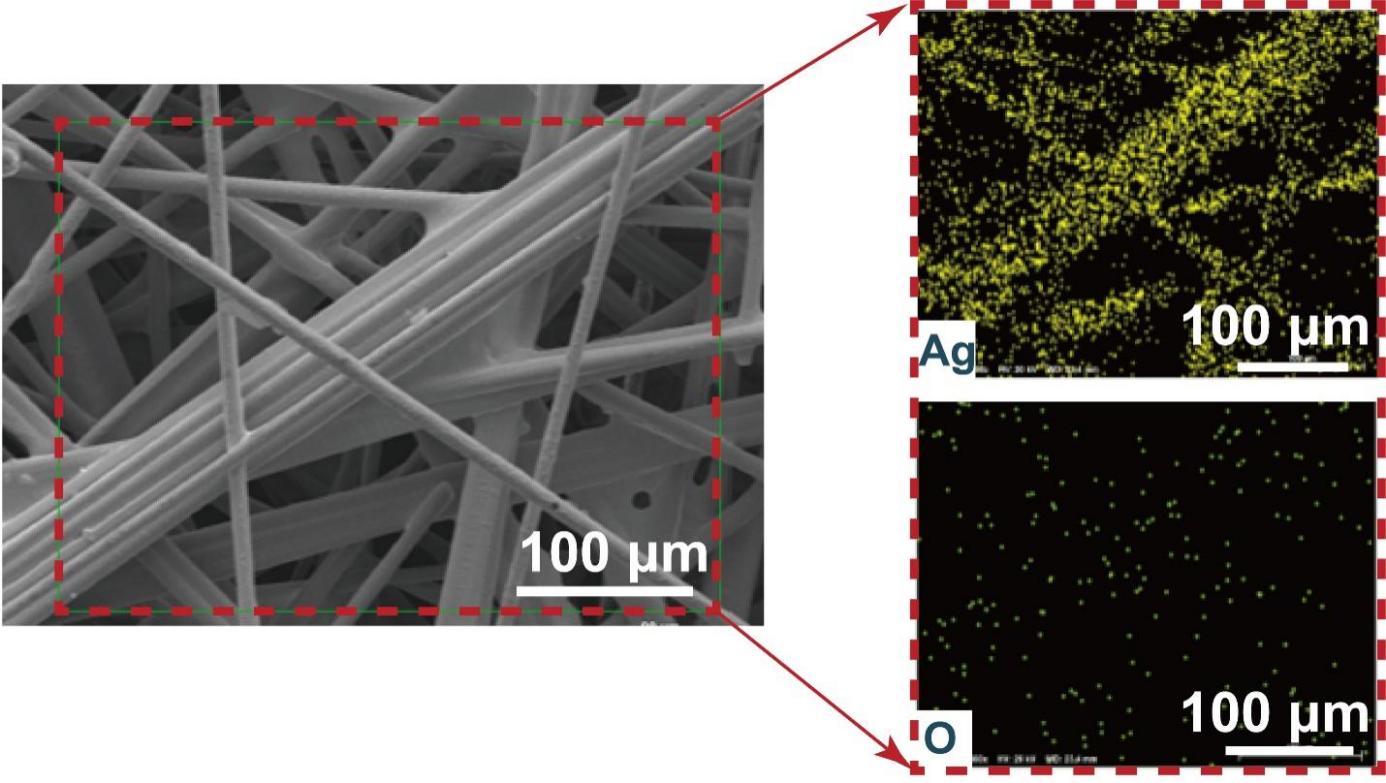


**Supplementary Figure 22. SEM image and EDS mapping analysis of the original Ag glass fiber electrode.**


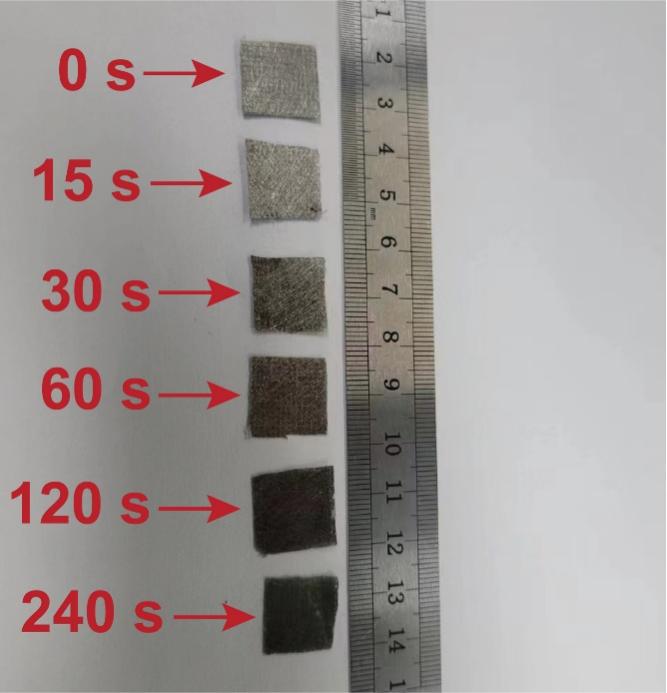


**Supplementary Figure 23. Potographs of the Ag/Ag_2_O glass fiber electrodes with different oxidation times.**


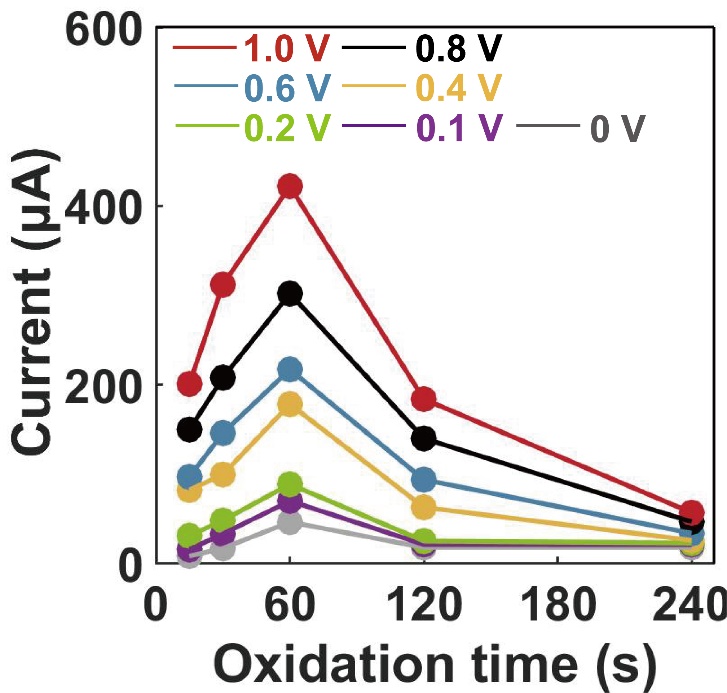


**Supplementary Figure 24. Relationship between the oxidation times of Ag/Ag_2_O glass fiber electrodes and the currents of the micropump at different potentials.**


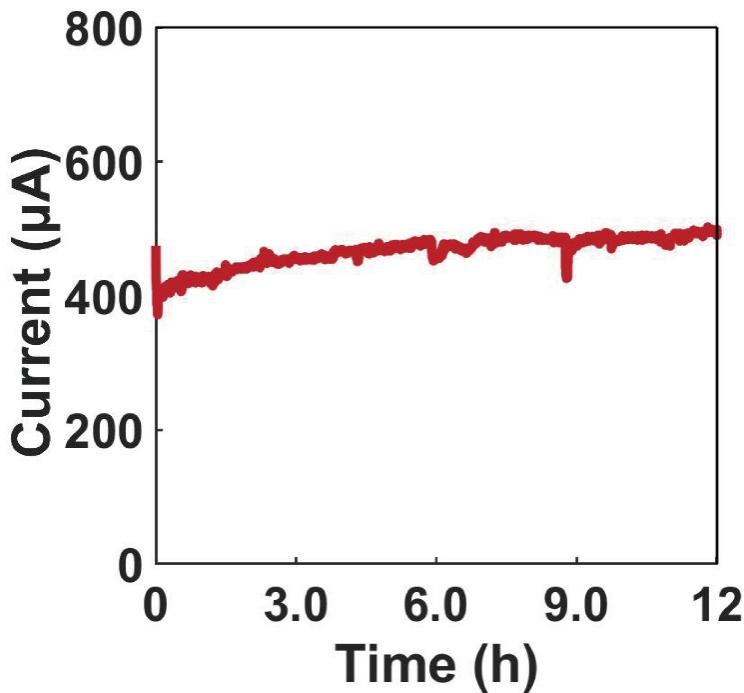


Supplementary Figure 25. Current-versus-time curves of the micropump operated under 1 V for 12 hours.


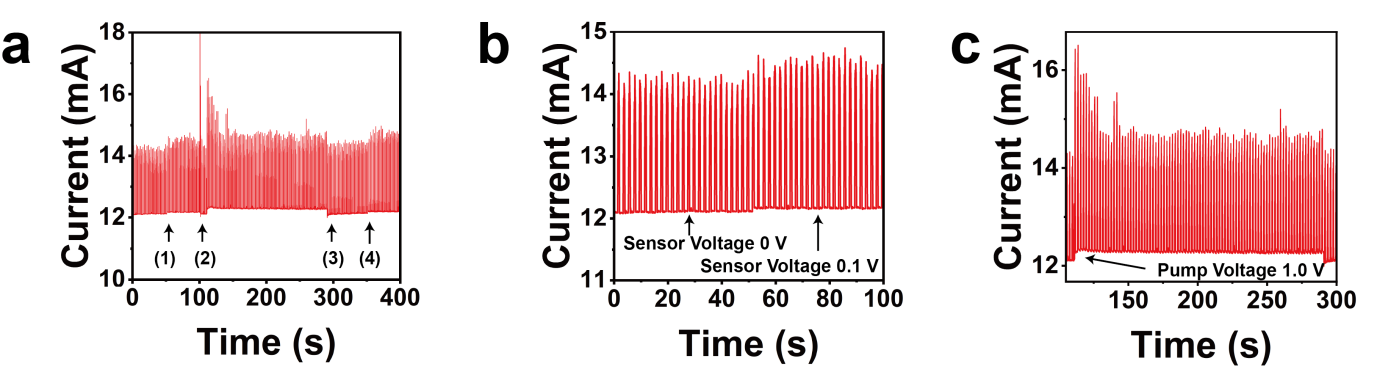


**Supplementary Figure 26. Current-versus-time curve of the power system in the closed-loop system during the 5-minute cycling process.** a, Current-versus-time curve of the closed-loop system when the control algorithm is activated. (1) sensor voltage 0.1 V for 50 s and 0 V for 10 s, (2) pump voltage 1.0 V for 180 s, (3) pump voltage 0 V for 60 s. (4) sensor voltage 0.1 V for 50 s and 0 V for 10 s. b, Current-versus-time curve of the closed-loop system during the sensor working (sensor voltage 0.1 V). c, Current-versus-time curve of the closed-loop system during the pump working (pump voltage 1 V).

Supplementary Figure 26 presents the current-time curves for power consumption analysis of the closed-loop system using an electrochemical workstation, simulating the standard potential of 3.7 V for lithium batteries. Supplementary Figure 26a displays the current variation over time during the 5-minute cycling process of the closed-loop system. As the circuit board reports current values to the smartphone every second, current peaks appear at one-second intervals. Supplementary Figure 26b illustrates the current-time variation when the board switches from standby (Sensor V= 0 V) to sensor operation mode (Sensor V= -0.1 V). According to the power consumption calculation formula (P=V×I), the standby mode shows an average current of 0.01263 A (46.73 mW power consumption), while sensor operation at 0.1V demonstrates 0.01272 A current (47.06 mW consumption). The power consumption of the sensing module is 0.33 mW. In standby mode, the average current is 0.01263 A. Excluding the peaks from Bluetooth communication, the average current is 0.01211 A, so the power consumption of the Bluetooth communication module is 1.92 mW. Supplementary Figure 26c depicts the current-time relationship during electroosmotic pump operation, showing 0.01289 A average current and 47.69 mW average power consumption. In the entire system, the micropump consumes 0.96 mW, accounting for 2.01% of the total power consumption. Calculated over 5-minute cycles, the system exhibits 0.01281 A average current and 47.39mW average consumption per cycle.


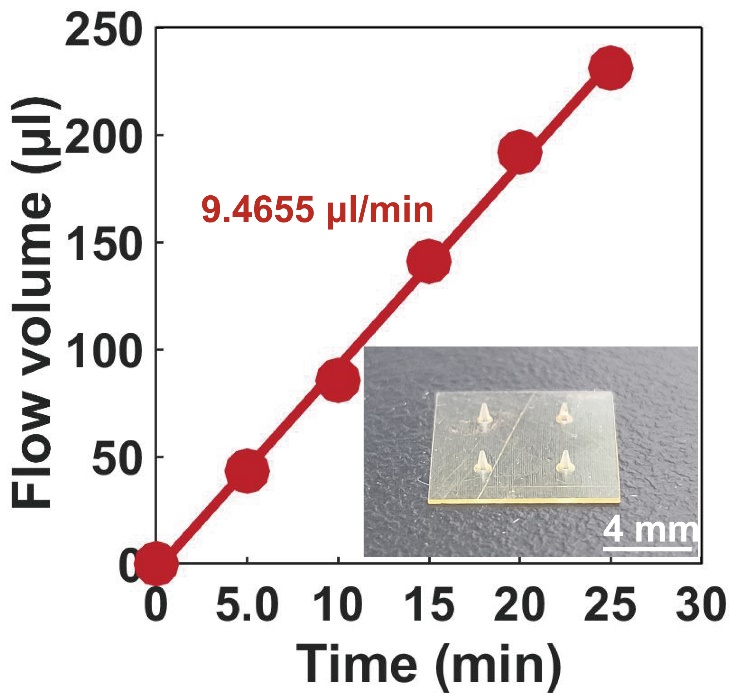


**Supplementary Figure 27. Insulin flow from a micropump connected to a hollow 3D printed microneedle array at 1 V voltage.** The dimensions of a hollow microneedle were 1.0 mm in height, 0.6 mm in base outer diameter, 0.4 mm in base inner diameter, 0.2 mm in tip outer diameter, and 0.1 mm in tip inner diameter. The distance between two microneedles was 4 mm.


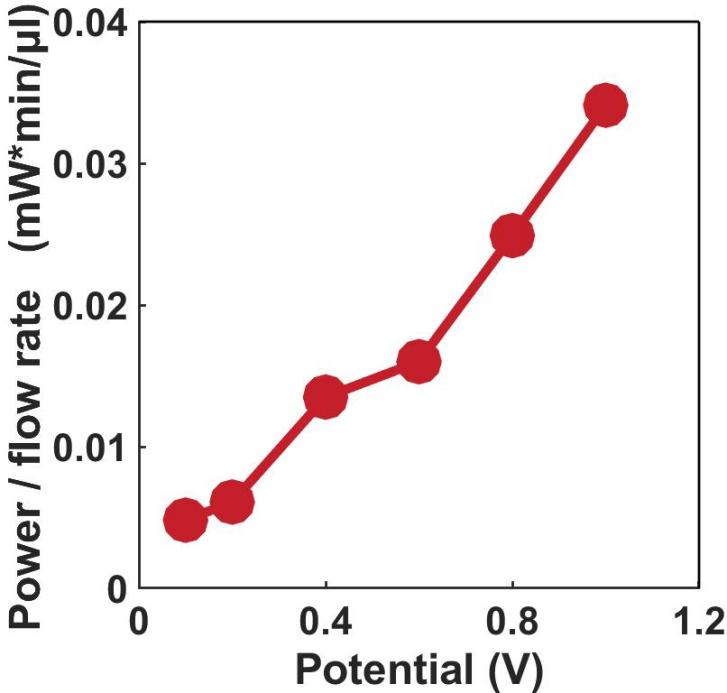


**Supplementary Figure 28. Power/flow rate of the micropump for releasing insulin at different potentials.**


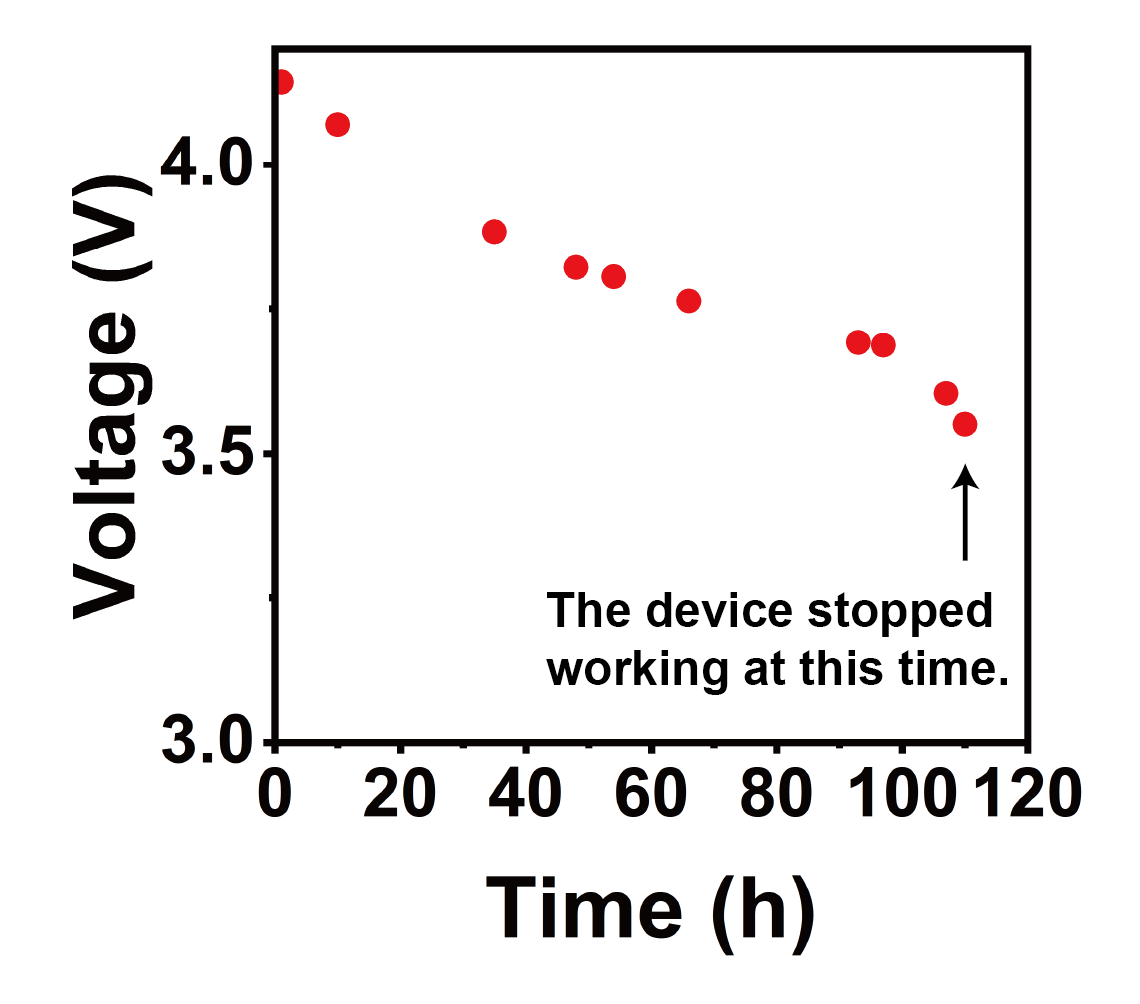


**Supplementary Figure 29. Voltage-versus-time curve of the lithium battery in the closed-loop system (battery capacity: 1500 mAh).**


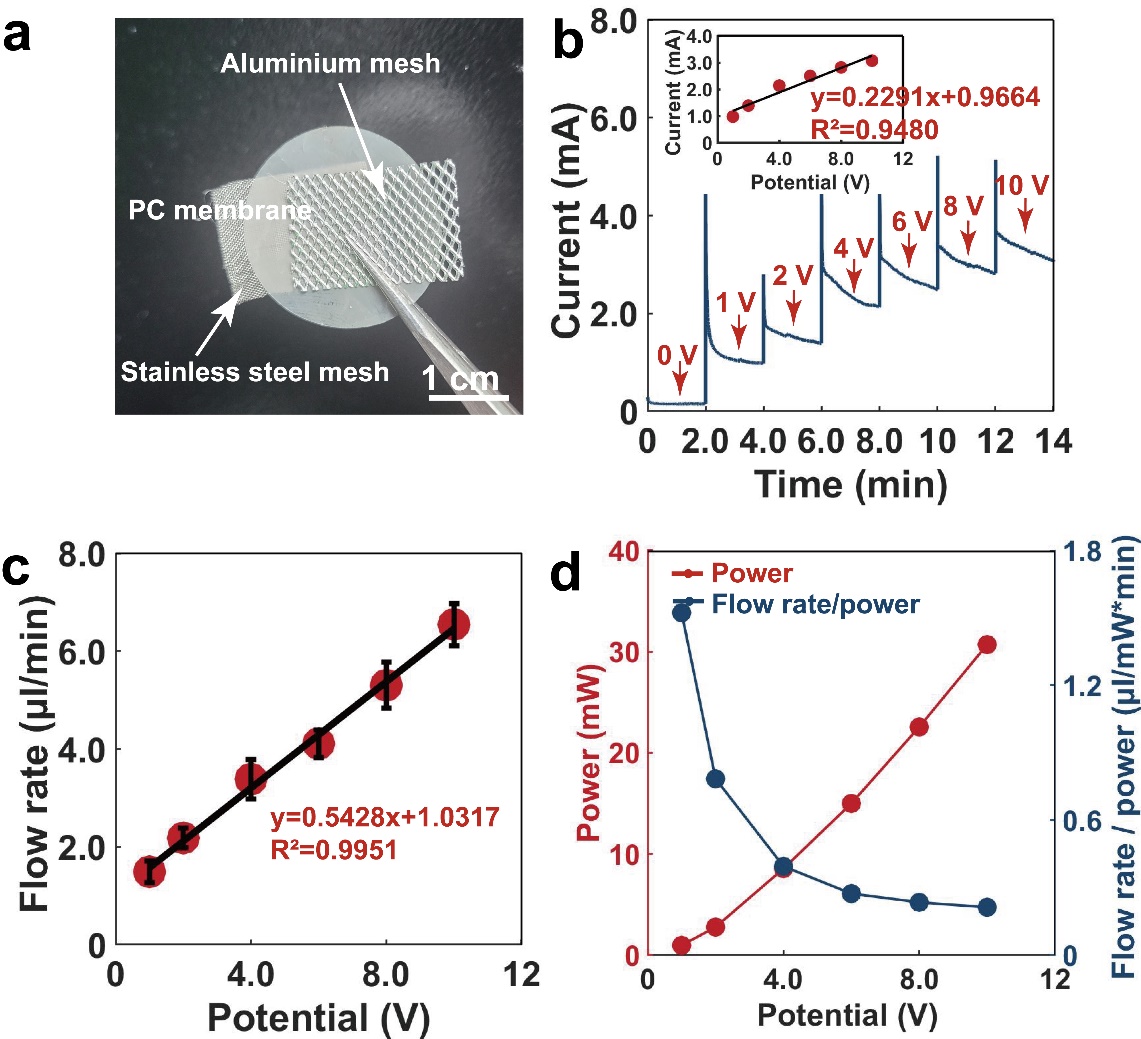


**Supplementary Figure 30. Characterization of the electroosmotic micropump with an aluminum mesh as the anode and a stainless-steel mesh as the cathode.** **a,** Camera image of the electroosmotic micropump. **b,** Potential and current needed by the micropump. **c,** Flow rates of insulin released by the micropump at different potentials (n=3). **d,** Power and flow rate/power of the micropump at different potentials.


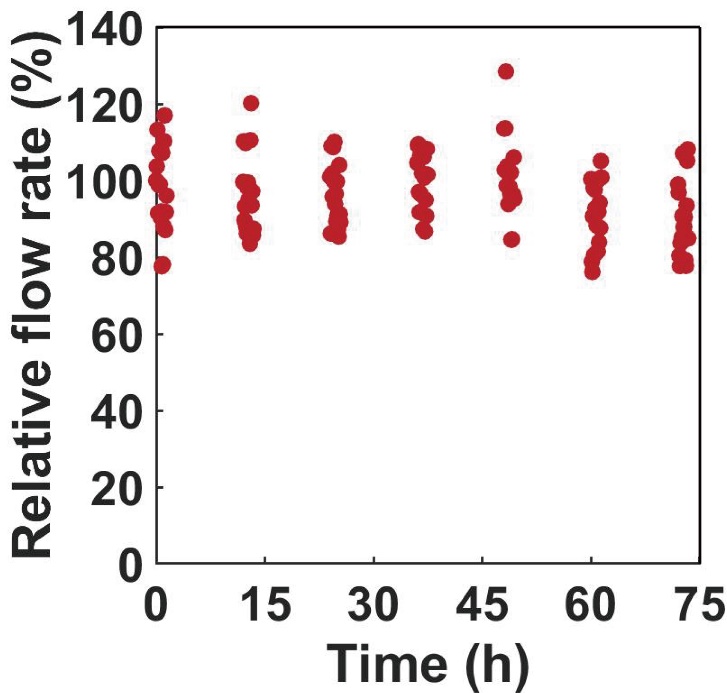


**Supplementary Figure 31. Basal rate accuracy of the micropump under 1 V over 72 h.**


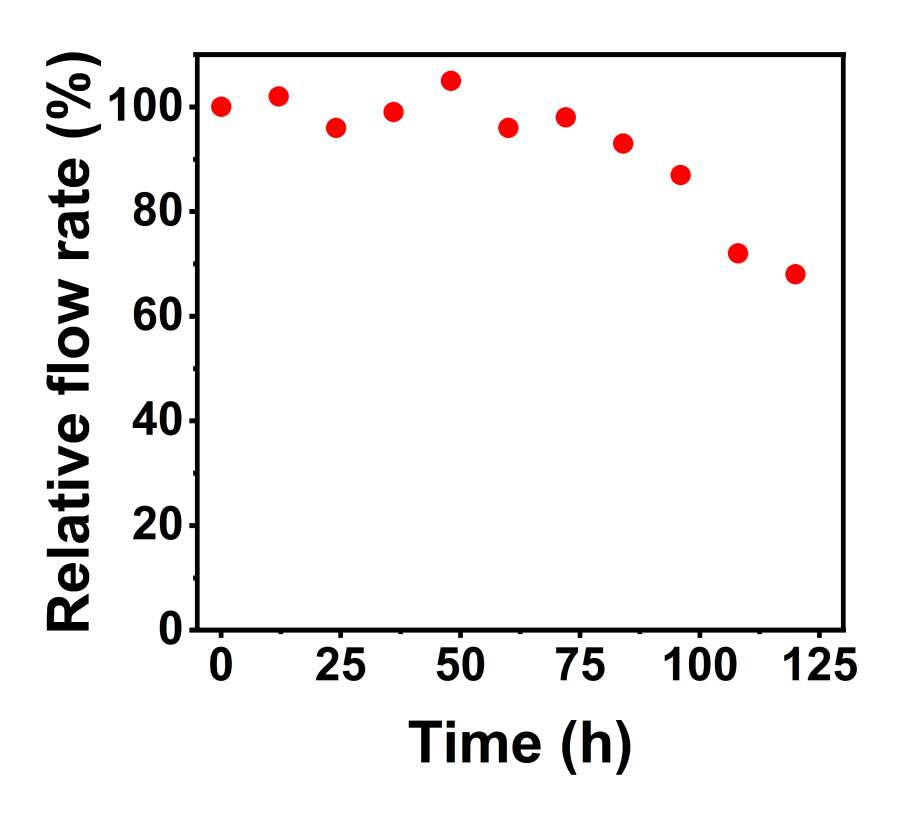


**Supplementary Figure 32. Relative flow rate of the electroosmotic pump changes over time when operating at a constant voltage of 1V for an extended period.**


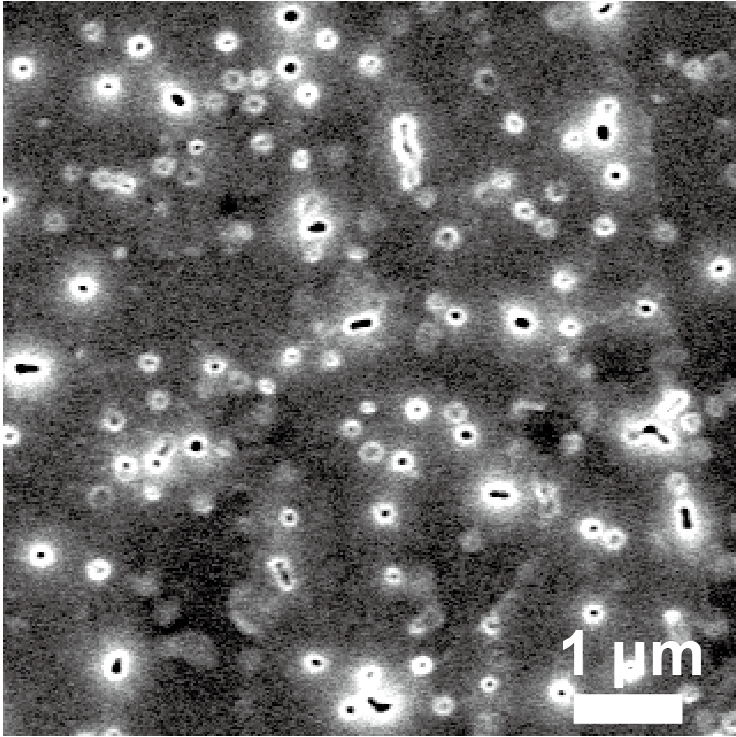


**Supplementary Figure 33. SEM image of the PC membrane after 4days of use.**


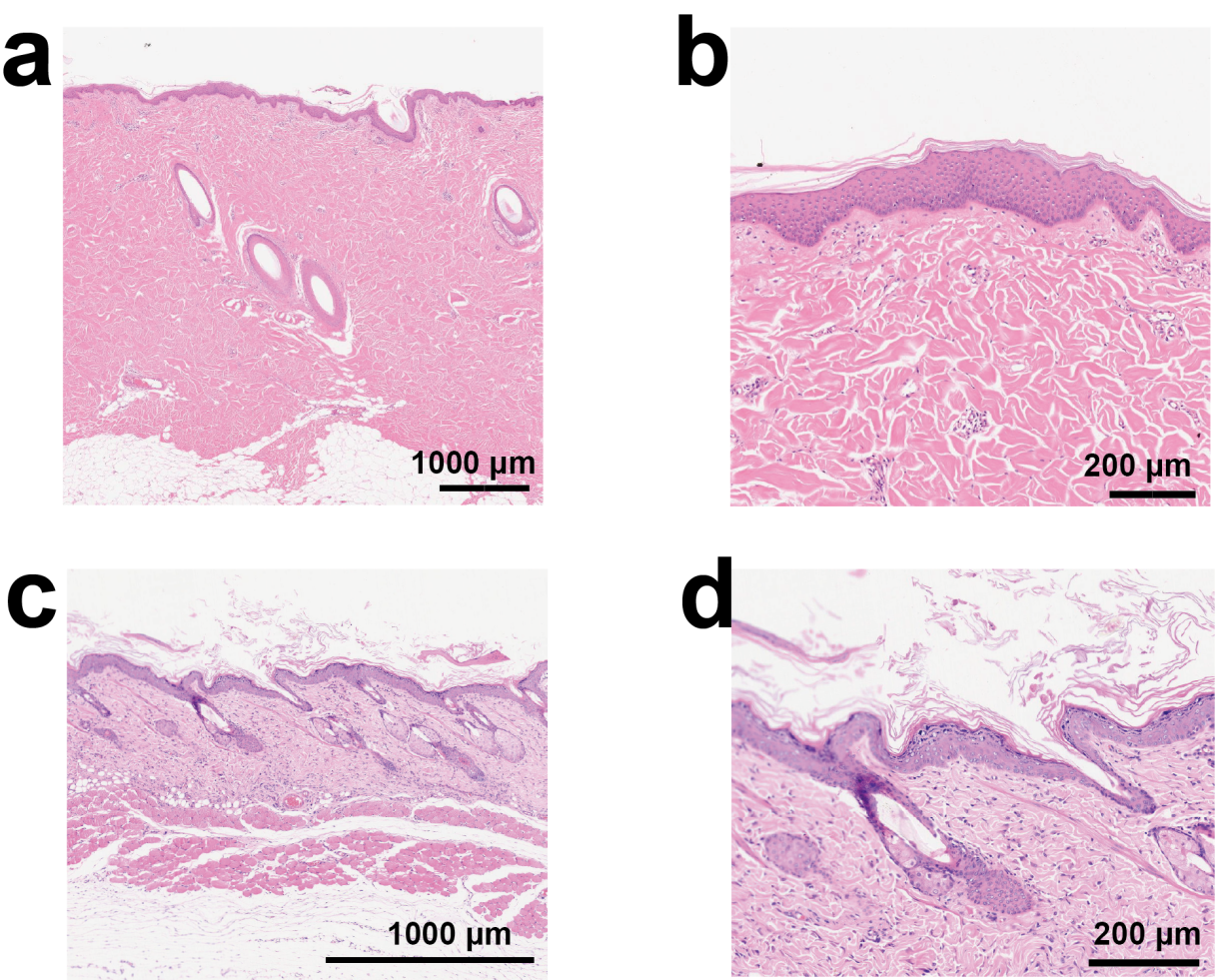


**Supplementary Figure 34. Optical images of hematoxylin-eosin (HE) staining of Bama pig and SD rat.** a, Bama pig dorsal skin dermis b, Bama pig dorsal skin epidermis.c, SD rat dorsal skin dermis d, SD rat dorsal skin epidermis.

The dorsal epidermal thickness of Bama pigs was 90.1 ±18.9 μm, with a dermal thickness of 4278 ±224 μm, while the dorsal epidermal thickness of SD rats was 49.37 ±2.82 μm, with a dermal thickness of 647 ±106 μm.


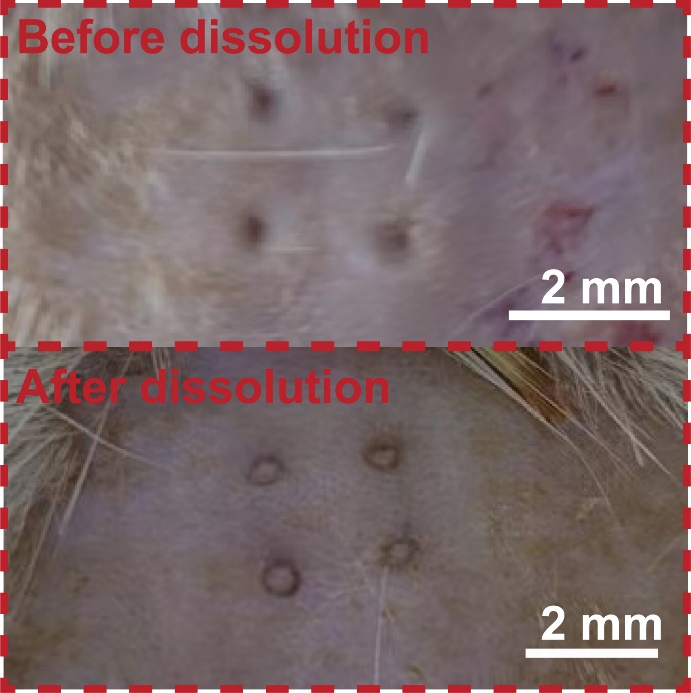


**Supplementary Figure 35. Pressure marks left on the skin of the back of a rat after wearing the system.** The dimensions of a microtube were 2.3 mm in height, 1.5 mm in outer diameter, and 1.0 mm in inner diameter. The dimensions of a dissolvable microneedle were 0.9 mm in diameter, 2.5 mm in cylinder height, and 0.5 mm in top cone height.


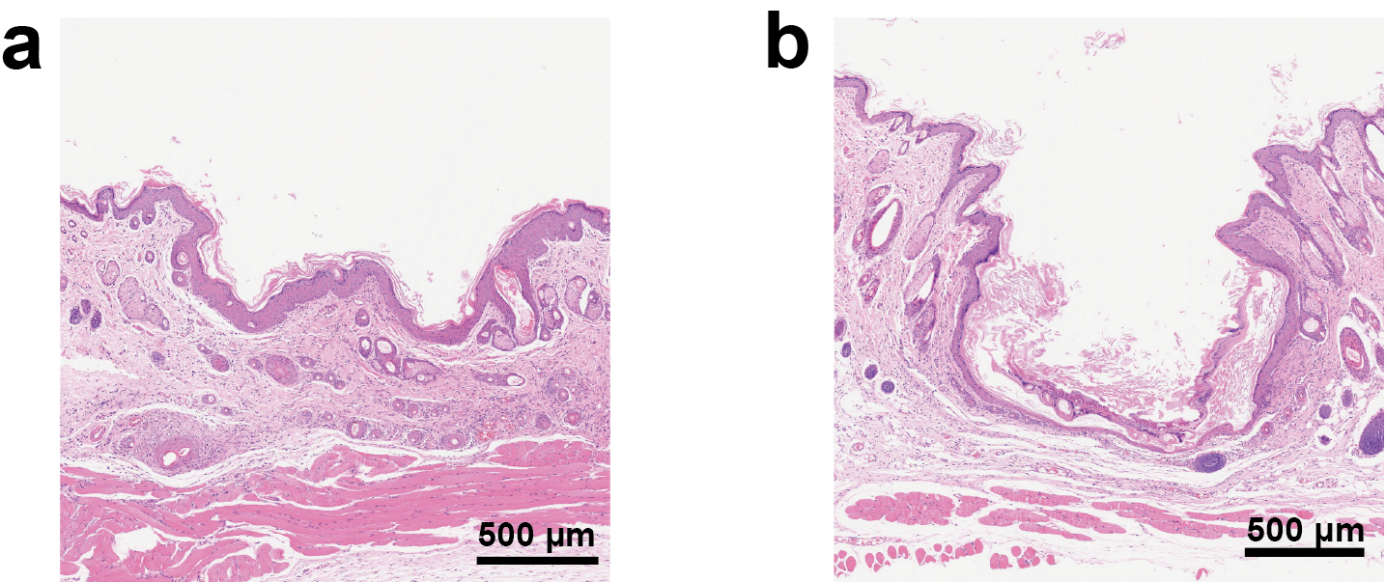


**Supplementary Figure 36. Optical images of HE-stained pierced rat back-skin sections after use of the patch.** a, HE-stained images of rat skin with only microtubules worn b, HE-stained images of rat skin with microtubules and soluble microneedles worn

The dimensions of a microtube were 2.3 mm in height, 1.5 mm in outer diameter, and 1.0 mm in inner diameter. The dimensions of a dissolvable microneedle were 0.9 mm in diameter, 2.5 mm in cylinder height, and 0.5 mm in top cone height.


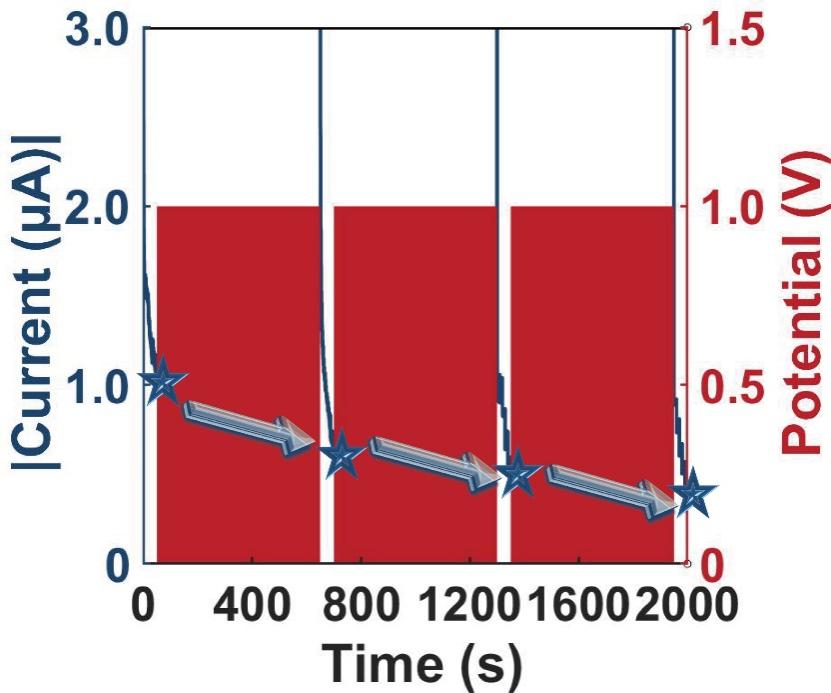


**Supplementary Figure 37. Operation model of the closed-loop system for managing diabetic rats’ blood glucose.** The blue lines represent the glucose sensing process (50 s) and the red bars represent the insulin delivery process (600 s).


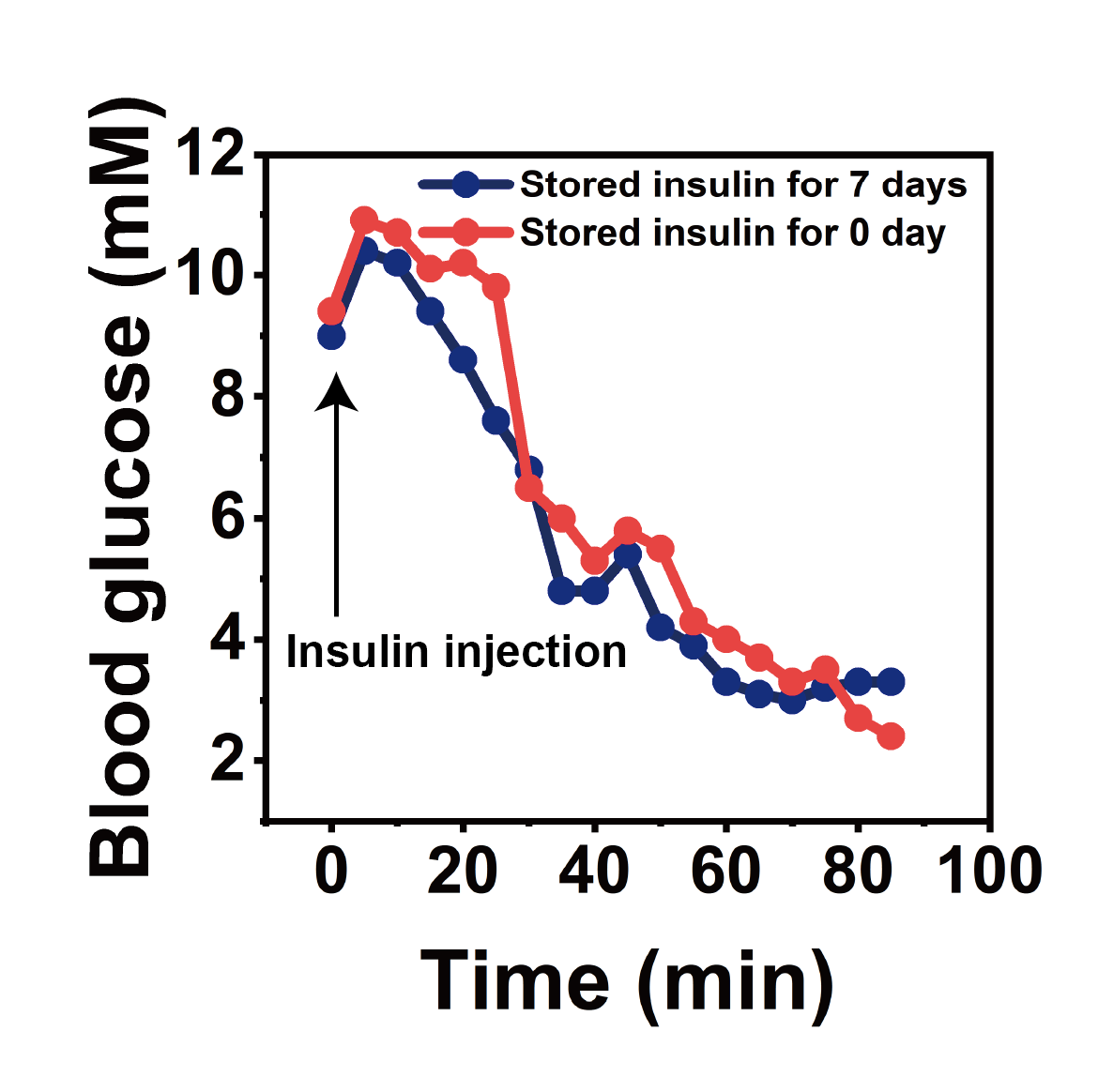


**Supplementary Figure 38. Blood glucose levels in SD rats over time after manual injection of insulin stored in the reservoir for 7 days and 0 days.**


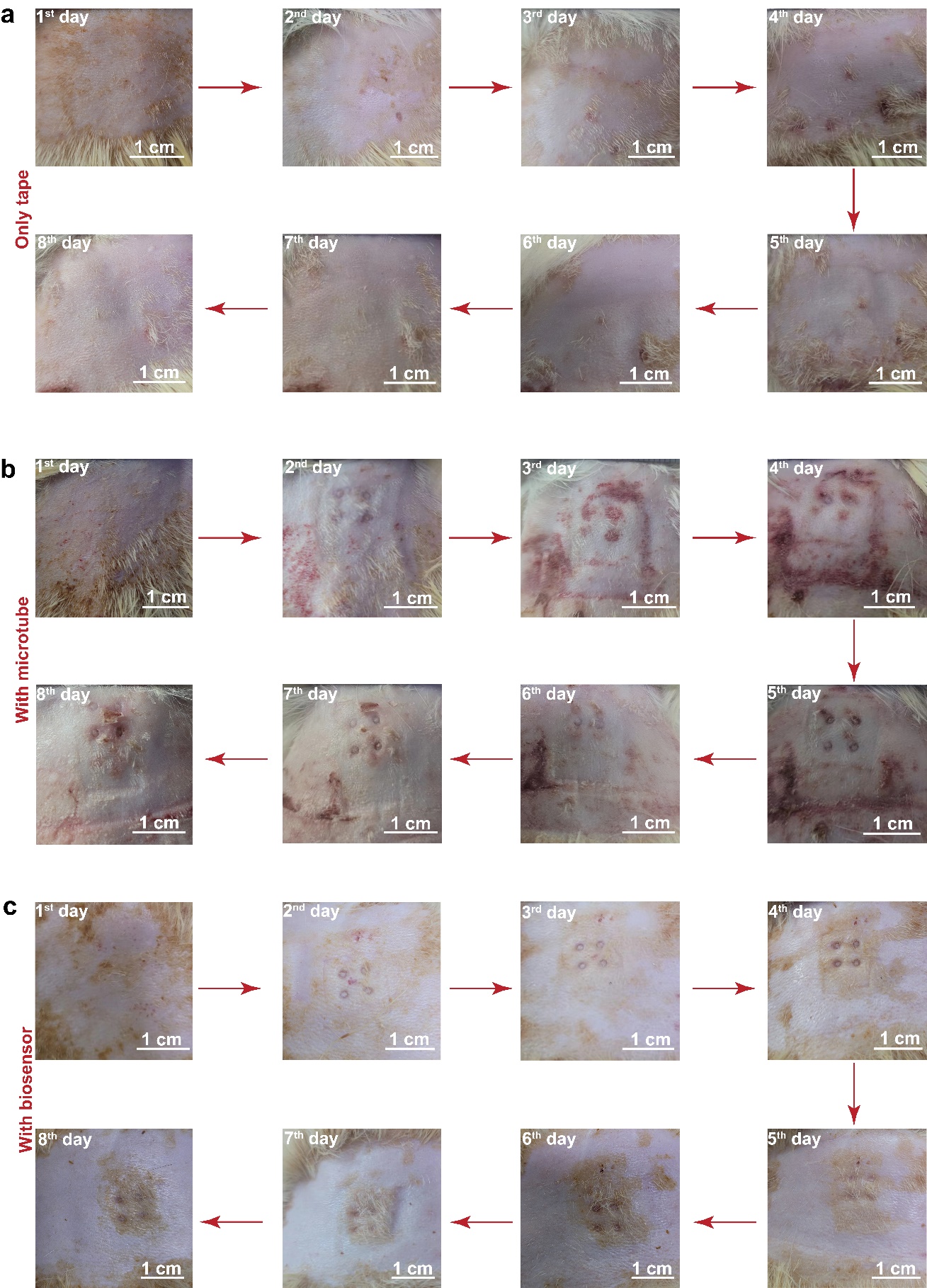


**Supplementary Figure 39. Rat skin irritation test for assessing *in-vivo* biocompatibility of the sensor.** **a,** Skin with only medical tape. **b,** Skin with bare microtubes. **c,** Skin with the biosensor.


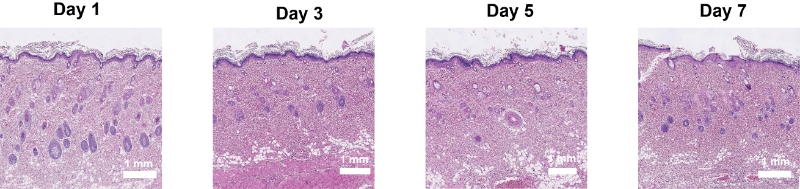


**Supplementary Figure 40. Optical images of hematoxylin-eosin (HE) staining of skin after wearing microneedles (day1, 3, 5 and 7).**


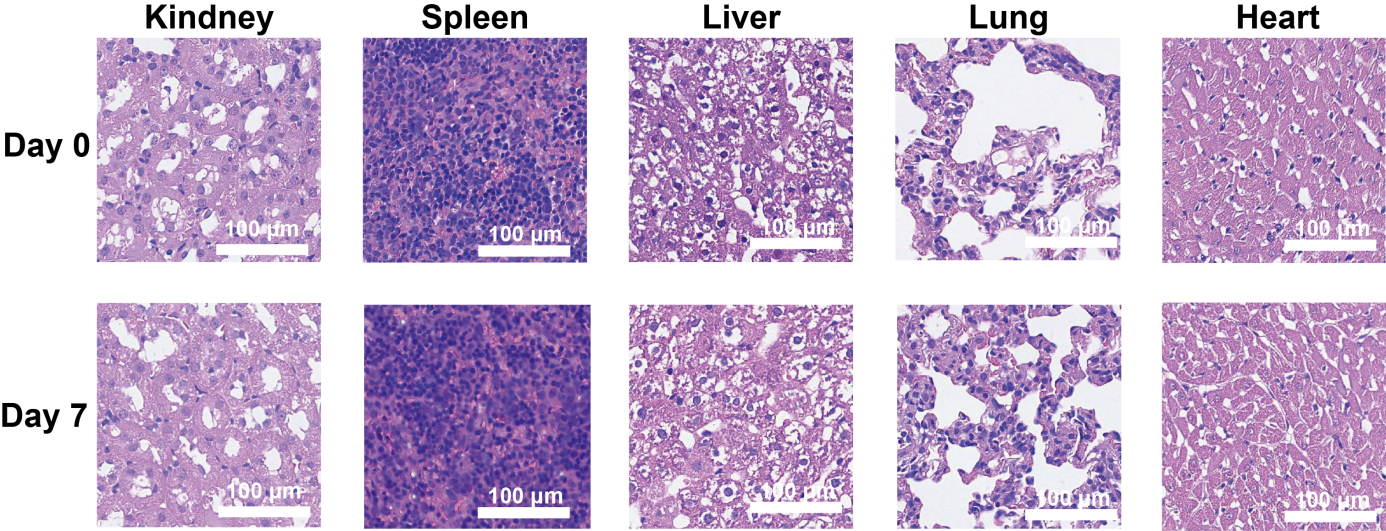


**Supplementary Figure 41. Optical images of hematoxylin-eosin (HE) staining of important organs (heart, liver, spleen, lung, and kidney) before and after wearing soluble microneedles.**


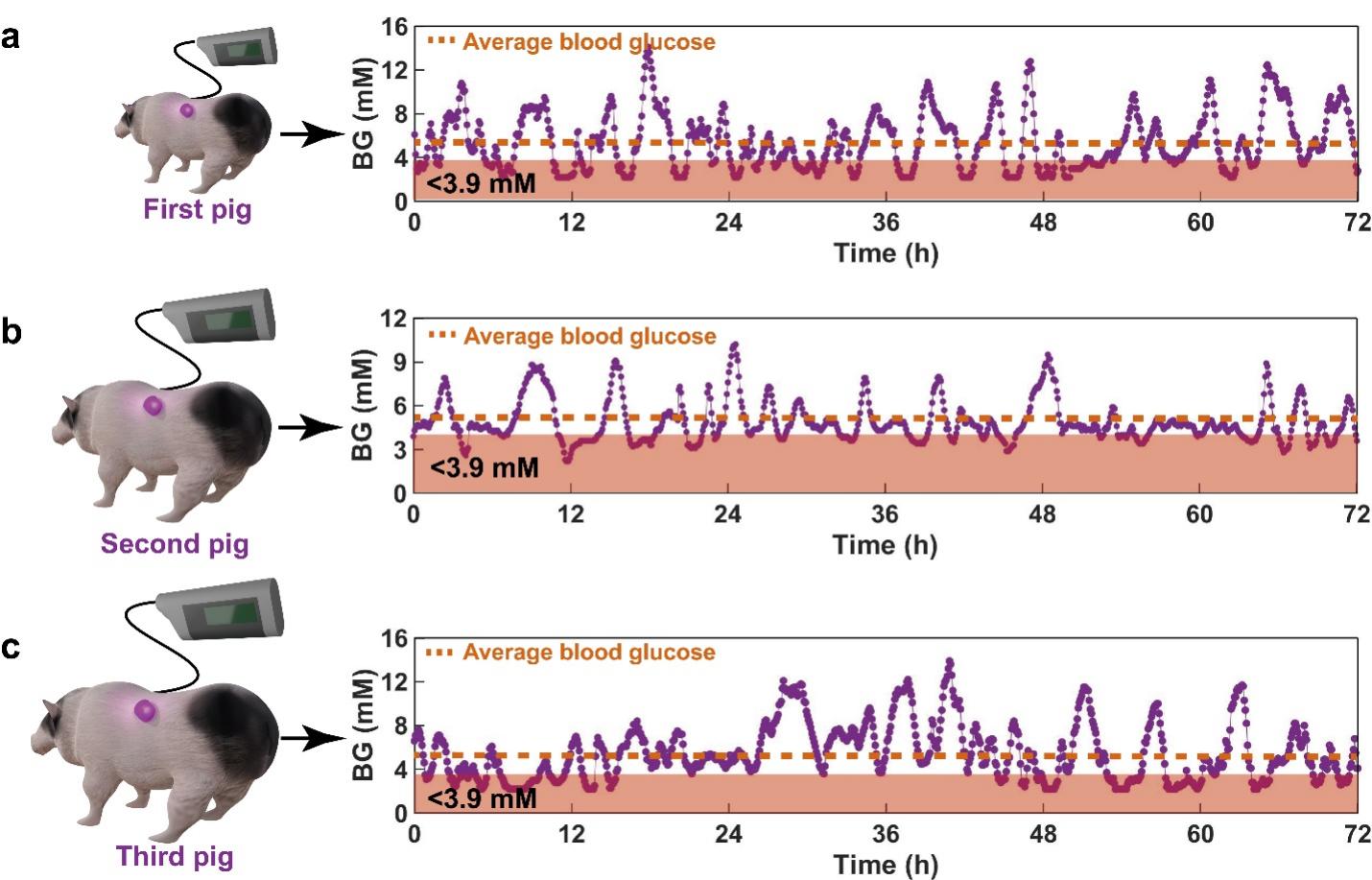


Supplementary Figure 42. Performance of the commercial system in three pigs with different basal insulin rates. a, The first pig with the basal insulin rate of 1 U/h. b, The second pig with the basal insulin rate of 1.2 U/h. c, The third pig with the basal insulin rate of 1.5 U/h. (The purple line represents the blood glucose measured by the commercial CGM, and the orange line represents the average blood glucose).

When the basal insulin rate was 1 U/h in the first pig, the time in tight range (TITR, 3.9-7.8 mM) was 46.71%, the time in range (TIR, 3.9-10.0 mM) was 59.77%, and the time below range (TBR, <3.9 mM) was 34.22%. When the basal insulin rate was 1.2 U/h in the second pig, the TITR was 72.72%, the TIR was 78.73%, and the TBR was 20.92%. When the basal insulin rate was 1.5 U/h in the third pig, the TITR was 53.18%, the TIR was 61.62%, and the TBR was 29.94%.


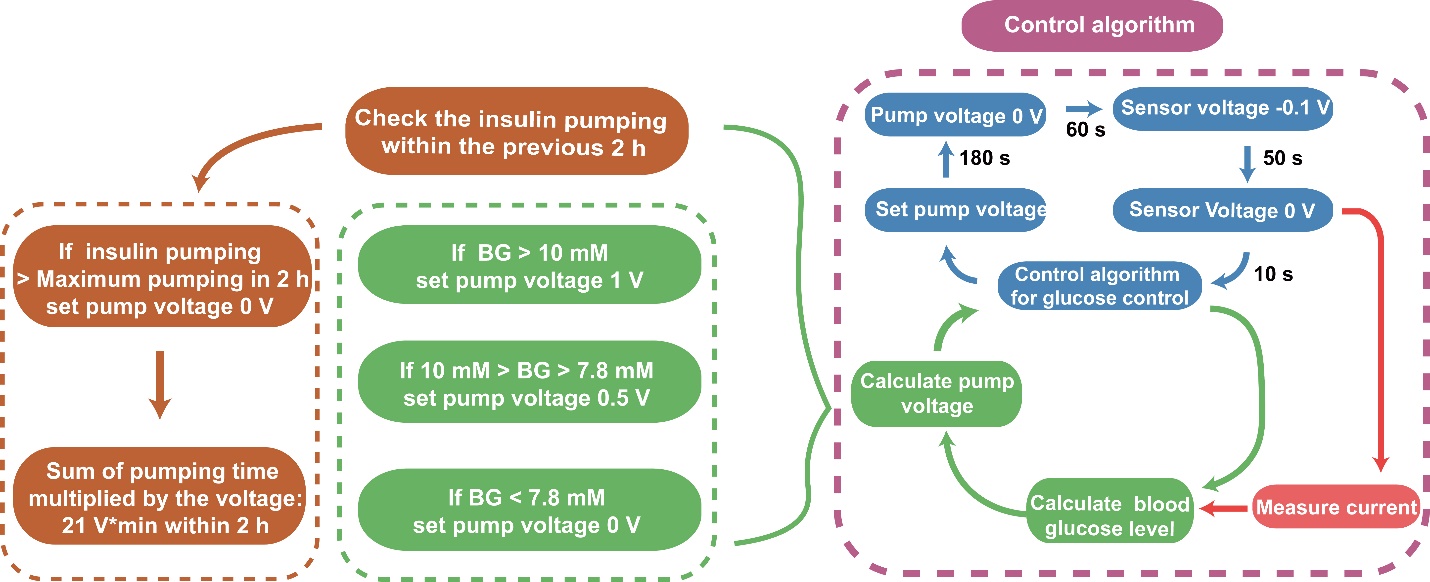


**Supplementary Figure 43. Hybrid closed-loop control algorithm for the system.**

The hybrid closed-loop control algorithm was designed. The glucose sensing process of the biosensor required 50 s to calculate the blood glucose level. After 10 s, the PCB determined the pumping potential based on the glucose detection results and activated the micropump to deliver insulin (100 U/ml) at a constant potential for 180 s. Subsequently, a 60 s period was allowed for insulin absorption in the skin. Within two hours, the sum of the pumping time multiplied by the pump voltage is set not to exceed 21 V·min. Moreover, approximately 40 min before food intake, the device could be manually set to continuously inject insulin for 10 min at 1 V to prevent excessive blood glucose elevation after a meal.


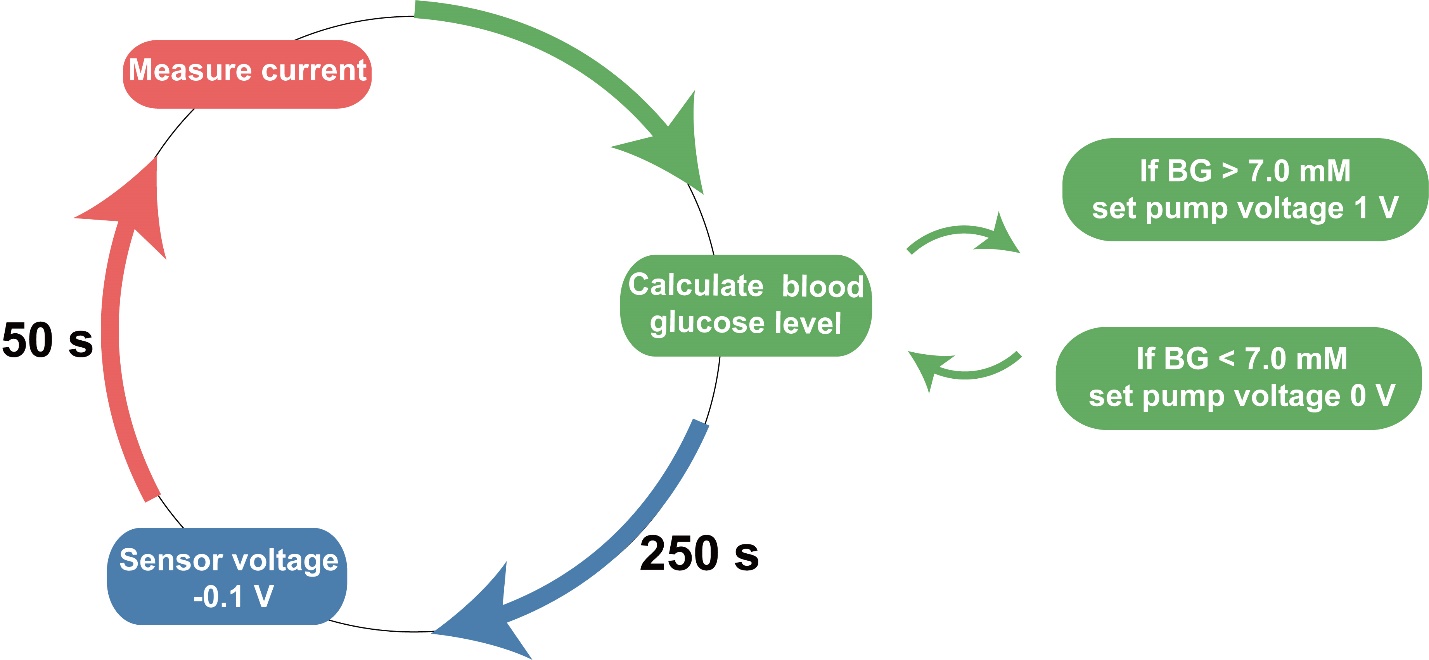


**Supplementary Figure 44. On-off control algorithm for the system.**

The on-off control algorithm was designed. Alternate sensing (50 s) and pumping (250 s) processes were used to control the operation of the closed-loop system. The glucose sensing process of the biosensor required 50 s to calculate the blood glucose level. If the blood glucose was higher than 7.0 mM, the PCB activated the micropump to deliver insulin (100 U/ml) at a constant potential for 250 s. The glucose sensing and insulin pumping processes would be repeated alternatively until the blood glucose was in the normal range.


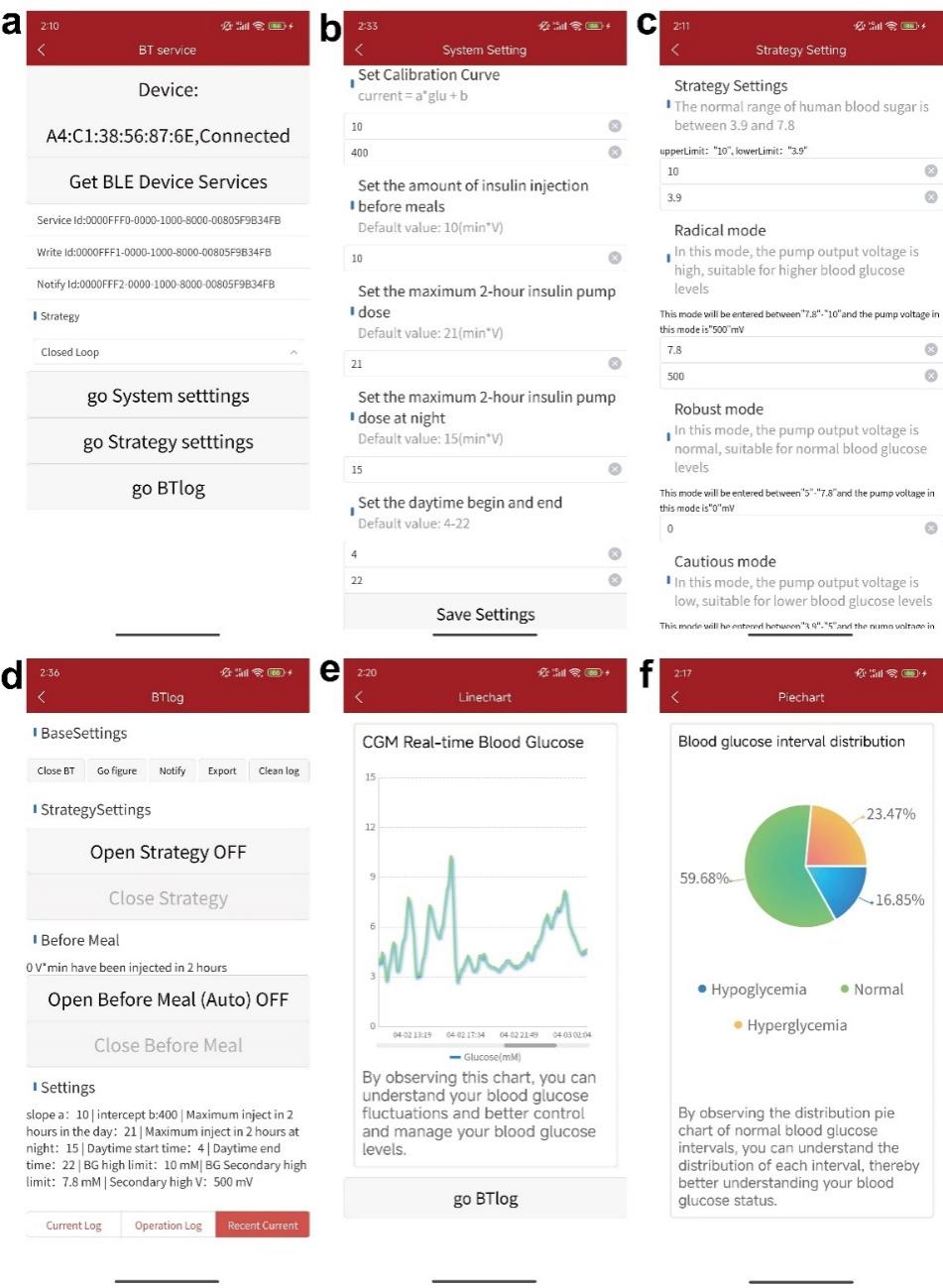


**Supplementary Figure 45. Mobile application developed for system control and data display. a,** Home page. **b,** System setting page. **c,** Strategy setting page. **d,** On/off system page. **e,** Real-time blood glucose change display. **f,** Real-time blood glucose distribution display.

A mobile application was designed to provide a user-friendly interface for the system control and data display. The application could establish a stable Bluetooth connection to the closed-loop patch, and users could control the patch on the application through setting up the calibration curve of the current from the biosensor and blood glucose, adjusting the insulin pumping potential and time. The application could also display the blood glucose data distribution measured by the biosensor in real time, and plot a graph of the blood glucose change versus time.


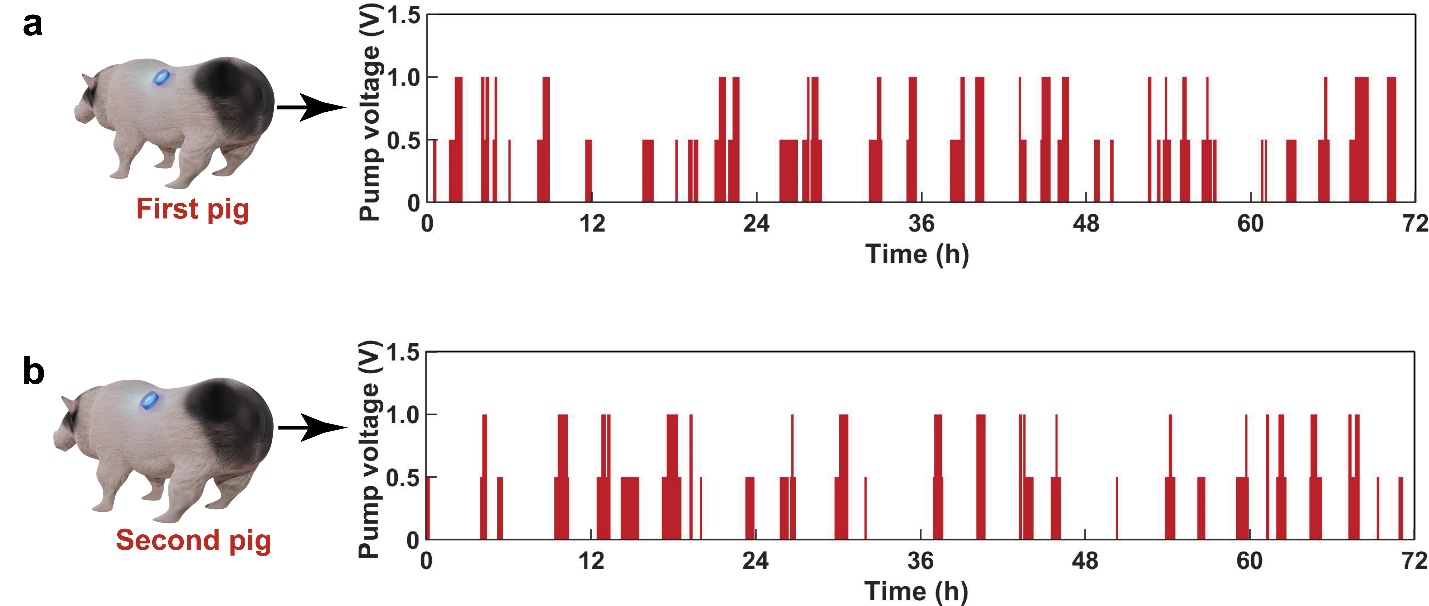


Supplementary Figure 46. Voltage of the insulin micropump at different times. a, The first pig. b, The second pig.

**Supplementary Figure 46** shows the the voltage of the insulin micropump at different times when the closed-loop patch with the control algorithm shown in **Supplementary Figure 44** was applied to the first and second pig.


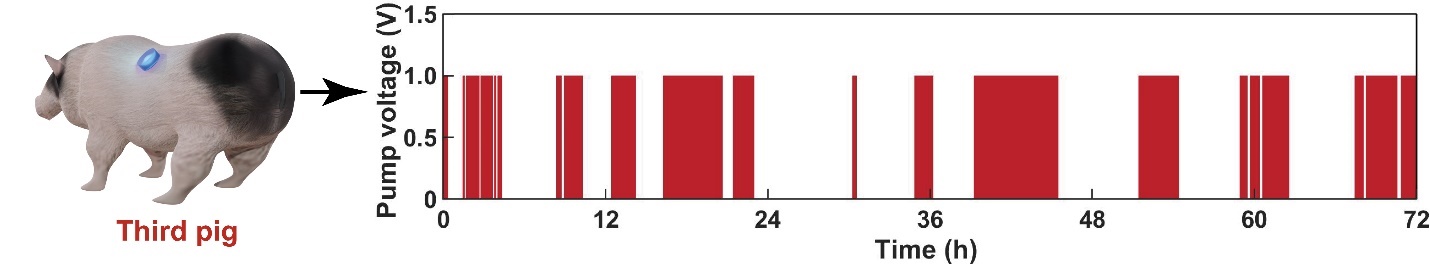


Supplementary Figure 47. Voltage of the insulin micropump at different times for the third pig.

**Supplementary Figure 47** shows the voltage of the insulin micropump at different times when the closed-loop patch with the control algorithm shown in **Supplementary Figure 44** was applied to the third pig.


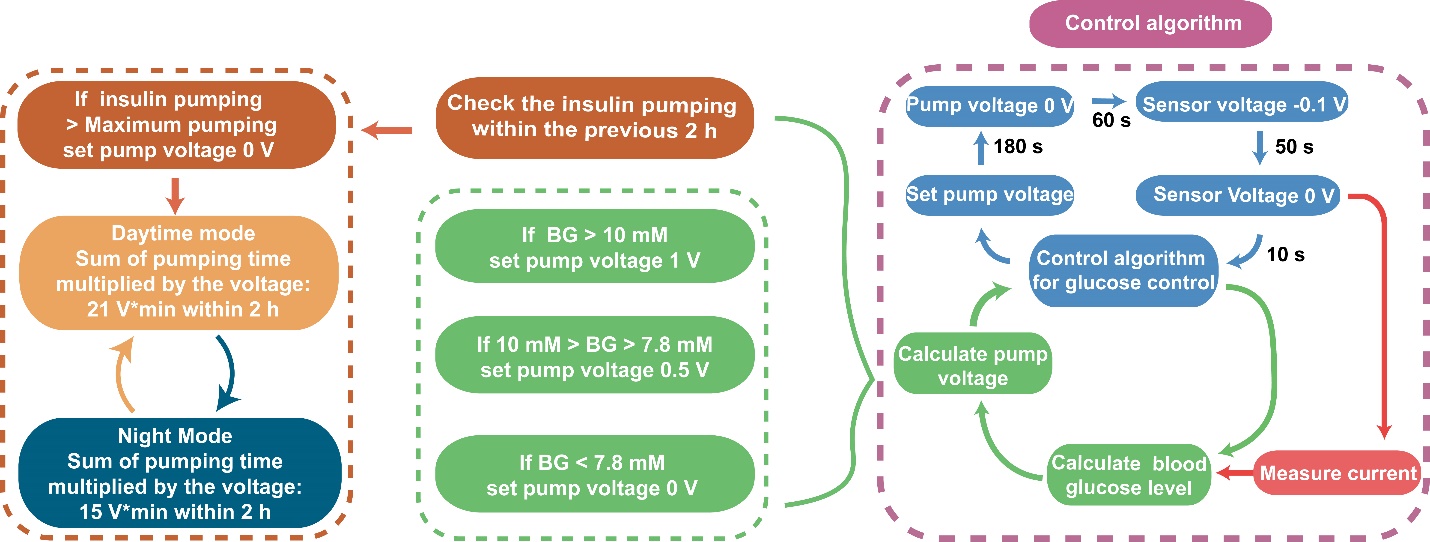


**Supplementary Figure 48. Distinct day-night hybrid closed-loop control algorithm for the system.**

The distinct day-night hybrid closed-loop control algorithm was designed to reduce the occurrence of nocturnal hypoglycemia. Compared to the control algorithm shown in **Supplementary Figure 29**, the sum of the pumping time multiplied by the pump voltage within two hours is set not to exceed 15 V·min from 10 p.m. to 4 a.m.


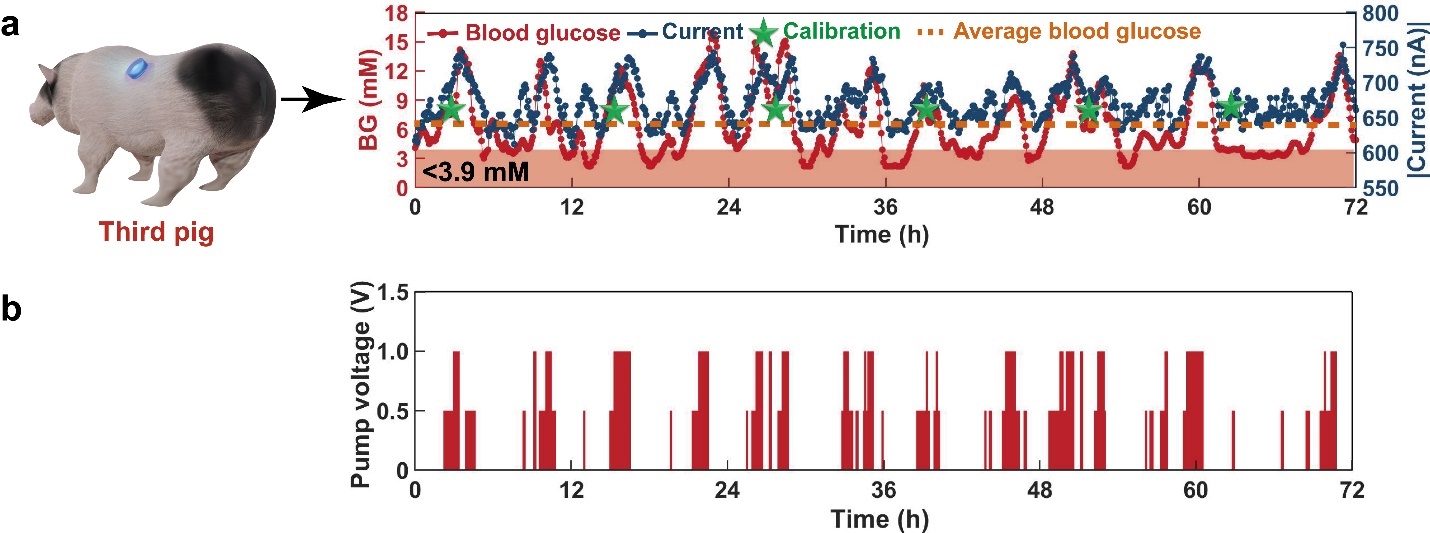


**Supplementary Figure 49**. **Performance of the closed-loop system with the hybrid control algorithm applied to the third pig.** **a,** Blood glucose and measured current change over 3 days. (The red line represents the blood glucose measured by the commercial CGM, the black-green line represents the current measured by the biosensor, the green pentagram mark represents the calibration time, and the orange line represents the average blood glucose). **b,** The corresponding voltage of the insulin micropump at different times.

The hybrid closed-loop control algorithm could also be applied to the third pig. Considering the weight and diabetes severity of the third pig, the sum of the pumping time multiplied by the pump voltage within two hours is set not to exceed 24 V·min. Under the closed-loop management system with this algorithm, the TITR for the third pig was 48.32%, the TIR was 60.58%, and the TBR was 22.77%. The average blood glucose level was 6.4 mM.


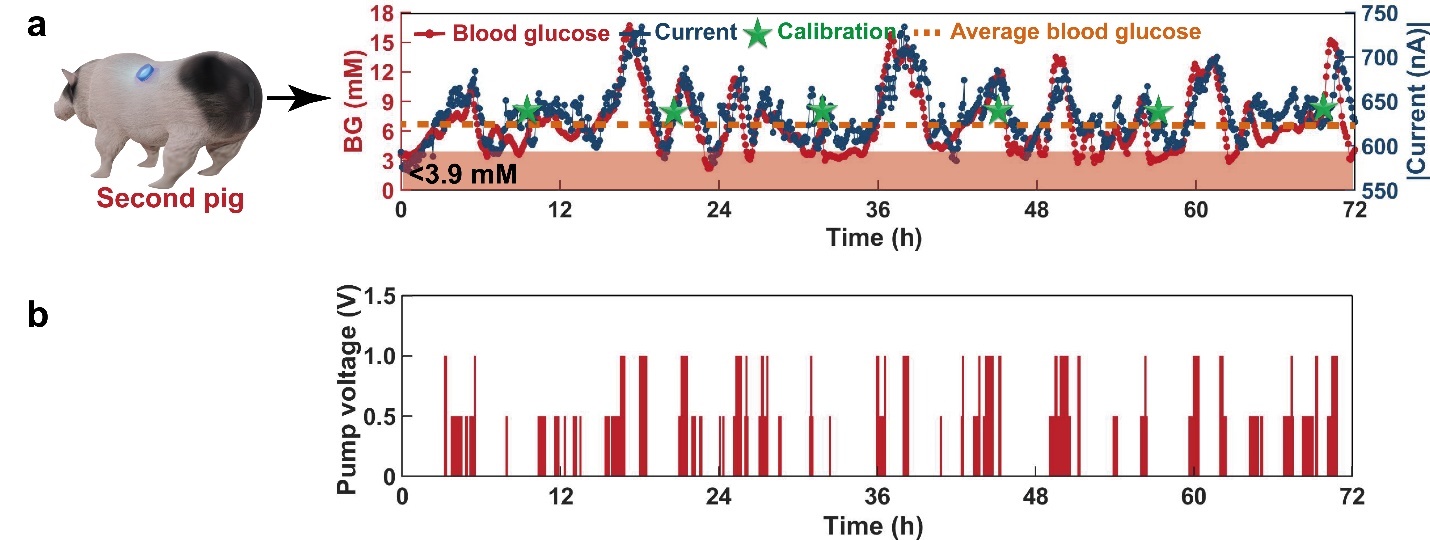


Supplementary Figure 50. Performance of the closed-loop system with the distinct day-night hybrid control algorithm applied to the second pig. a, Blood glucose and measured current change over 3 days. (The red line represents the blood glucose measured by the commercial CGM, the black-green line represents the current measured by the biosensor, the green pentagram mark represents the calibration time, and the orange line represents the average blood glucose). b, The corresponding insulin micropump voltage at different time.

The distinct day-night hybrid control algorithm closed-loop control algorithm could also be applied to the second pig. The occurrence of nocturnal hypoglycemia was reduced. The TITR over 3 days was 57.11%, the TIR was 67.51%, and the TBR was 16.30%. The average blood glucose level was 4.9 mM.


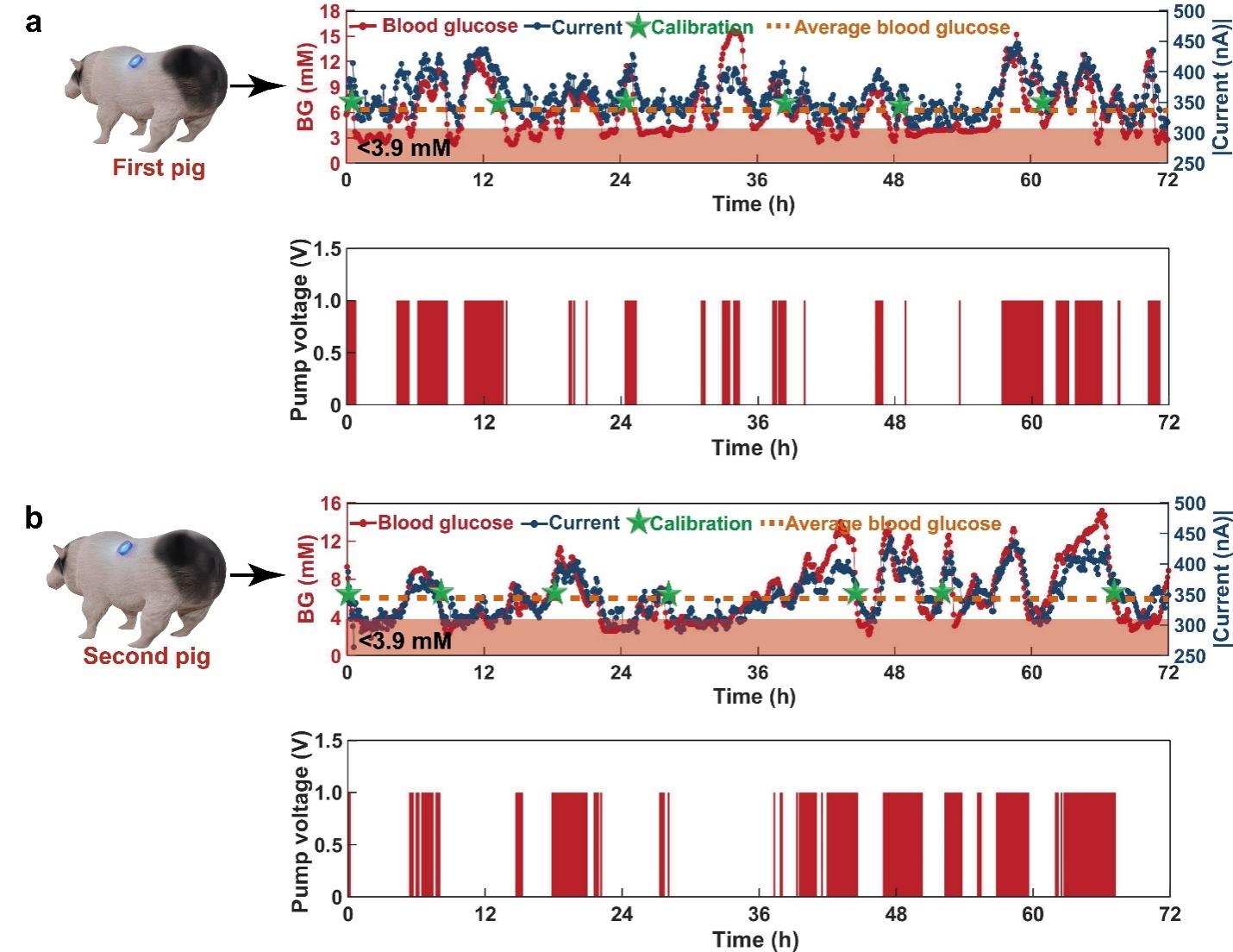


**Supplementary Figure 51.** **Performance of the closed-loop system with the on-off control algorithm applied to the first and second pig.** **a,** Blood glucose and measured current changes of the first pig over 3 days, and the corresponding insulin micropump voltage at different times. **b,** Blood glucose and measured current changes of the second pig over 3 days, and the corresponding insulin micropump voltage at different times. (The red line represents the blood glucose measured by the commercial CGM, the black-green line represents the current measured by the biosensor, the green pentagram mark represents the calibration time, and the orange line represents the average blood glucose).

The on-off control algorithm could also be applied to the first and second pig. Under the closed-loop management system with this algorithm, the TITR over 3 days was 48.21% for the first pig and 48.55% for the second pig. The TIR was 62.66% for the first pig and 61.04% for the second pig. The TBR was 25.78% for the first pig and 22.31% for the second pig. The average blood glucose level was 6.2 mM for the first pig and 6.4 mM for the second pig.


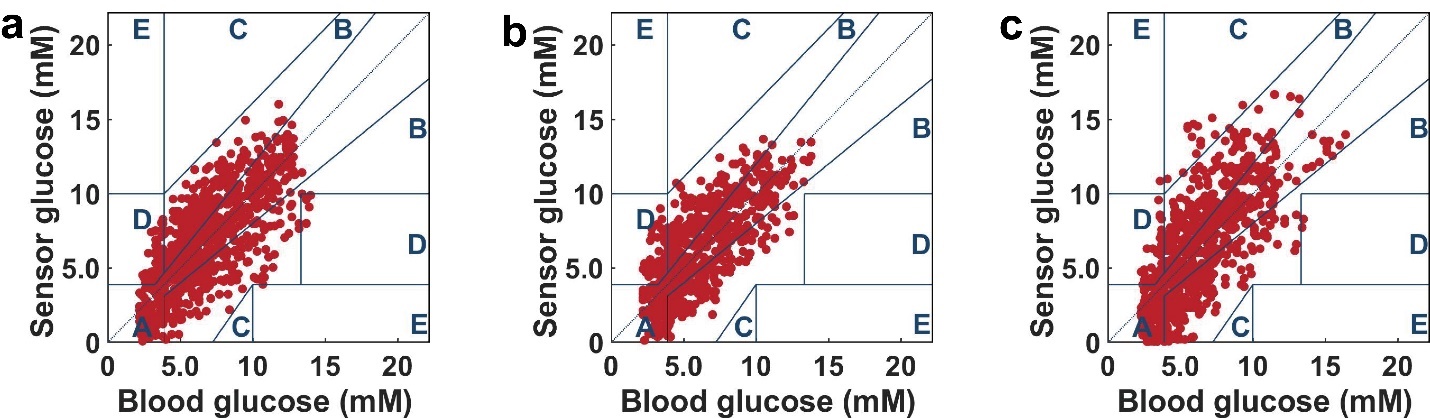


Supplementary Figure 52. Clarke error grid analysis. a, The first pig. b, The second pig. c, The third pig.


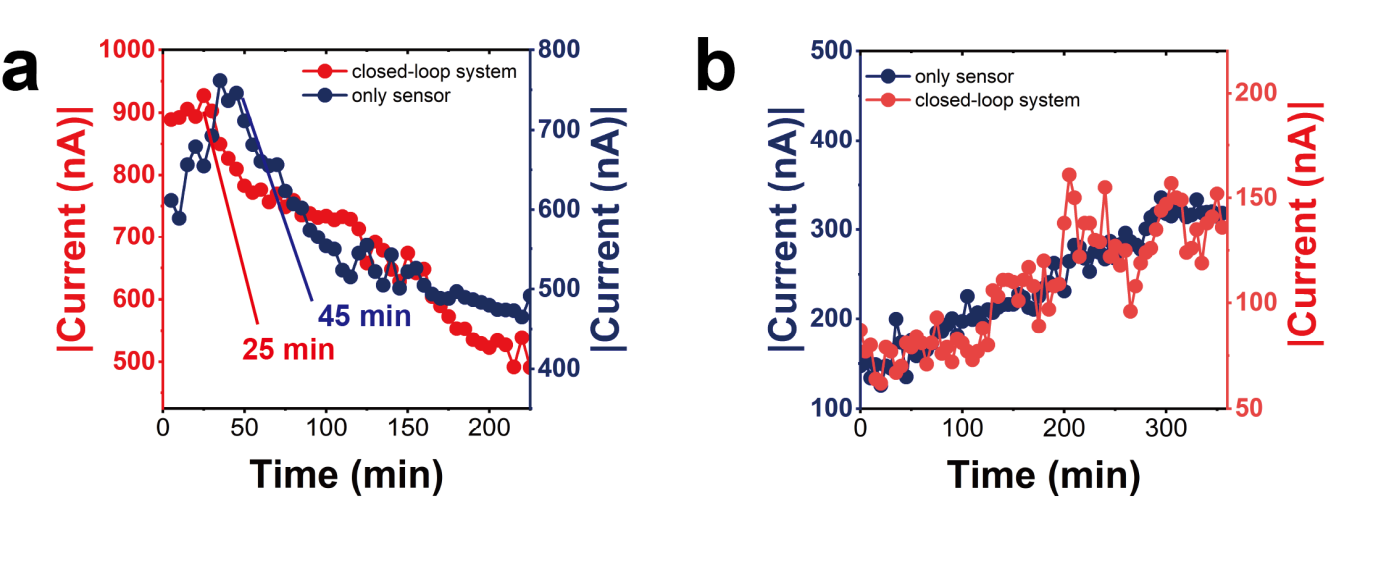


**Supplementary Figure 53. Time lag between the pig’s blood glucose change measured by the closed-loop system and the current measured by only the biosensor.**


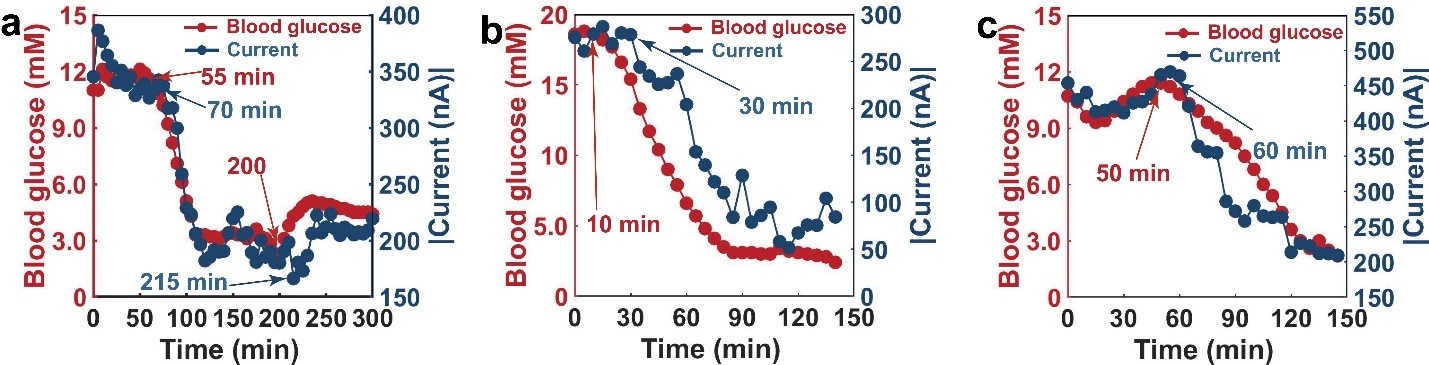


**Supplementary Figure 54. Time lag between the pig’s blood glucose change measured by the CGM and the current measured by the biosensor.** **a,** The first pig. **b,** The second pig. **c,** The third pig.


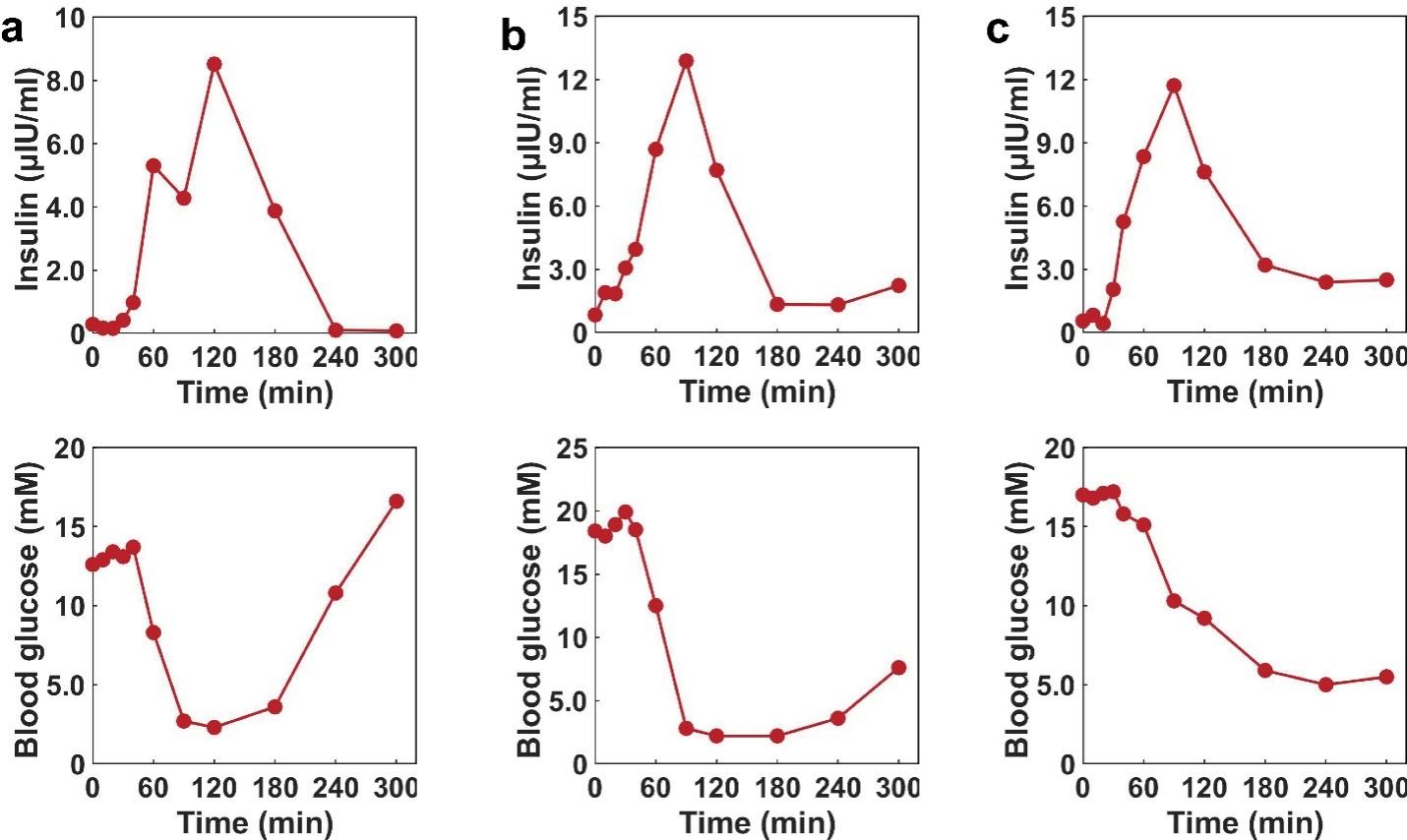


**Supplementary Figure 55.** **Insulin adsorption pharmacokinetics and pharmacodynamics profiles in three pigs following a 10-min insulin release with the closed-loop system.** **a,** The first pig. **b,** The second pig. **c,** The third pig.


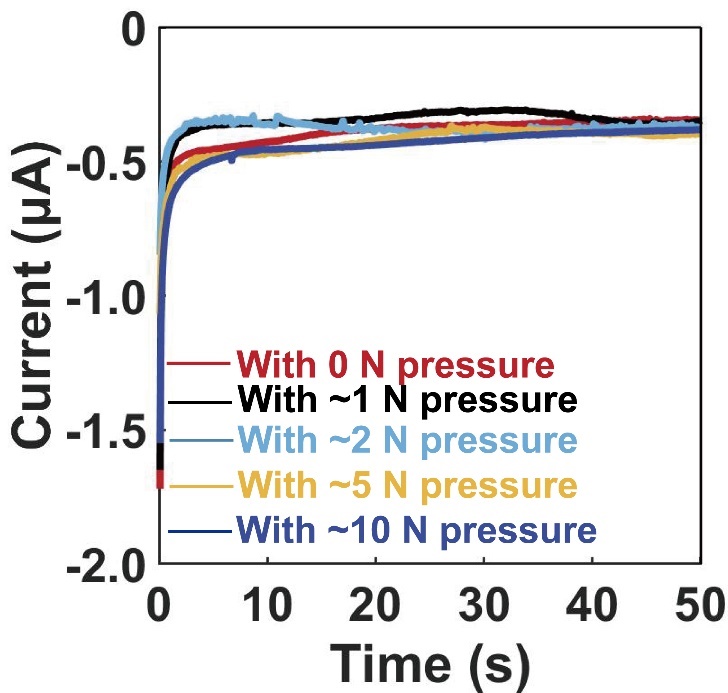


**Supplementary Figure 56. Current measured by the biosensor on the pig with different continuous vertical pressures.**

When the patch was applied to the pig, external continuous pressure had a minimal effect on the biosensor. The sensing current of the biosensor remained nearly constant under various continuous vertical pressures (ranging from about 1 N to 10 N).


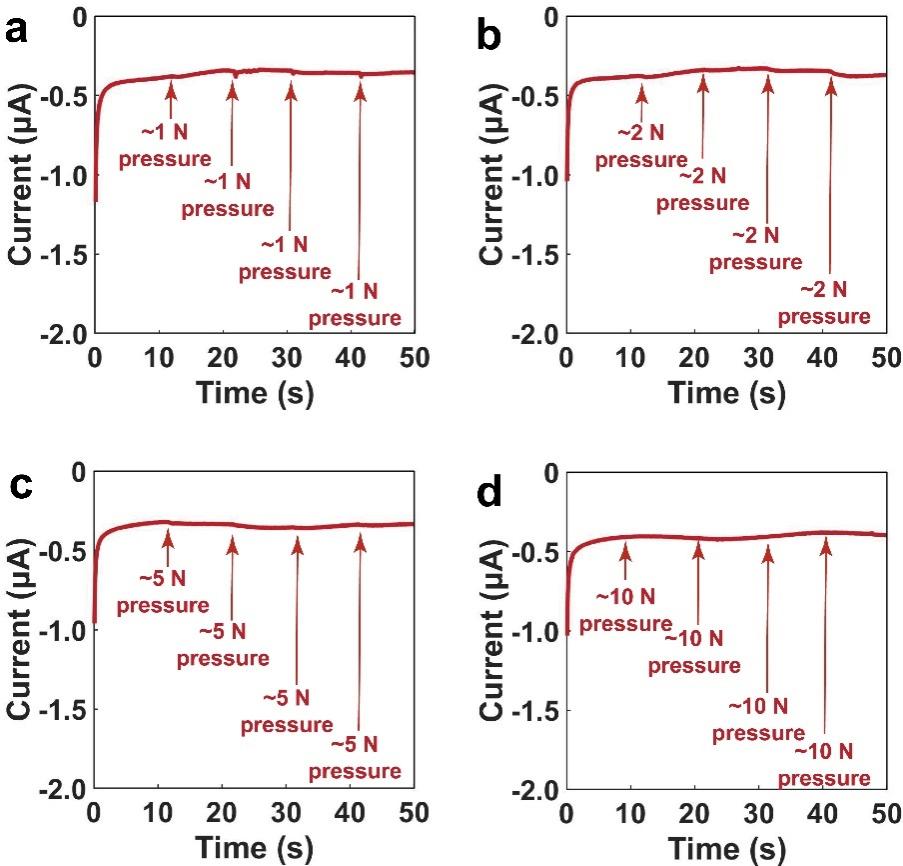


**Supplementary Figure 57. Current measured by the biosensor on the pig with different** **instantaneous vertical pressures.** **a,** ~1 N. **b,** ~2 N. **c,** ~5 N. **d,** ~10 N.

When the patch was applied to the pig, external instantaneous pressure also had a minimal effect on the biosensor. When different instantaneous pressures were applied, the current curve fluctuated but quickly returned to the original level after the pressure was removed.

**S****upplementary Table 1. Insulin sensitivity factor (ISF) and insulin-to-carbohydrate ratio (ICR) of pigs in three days.**

|  |  | **First day** | **Second day** | **Third day** | **Average value** |
| --- | --- | --- | --- | --- | --- |
| First pig (Moedling time: 2-5 month, weight: ~25 kg) | ISF  (mM/unit insulin) | 1.49 | 1.4 | 1.3 | 1.40 ± 0.08 |
|  | ICR  (g carb/unit insulin) | 30.00 | 30.00 | 30.00 | 30.00 |
| Second pig (Moedling time: 11-14 month, weight: ~35 kg) | ISF  (mM/unit insulin) | 2.24 | 2.12 | 2.13 | 2.16 ± 0.05 |
|  | ICR  (g carb/unit insulin) | 37.50 | 37.50 | 37.50 | 37.50 |
| Third pig (Moedling time: 16-19 month, weight: ~37 kg) | ISF  (mM/unit insulin) | 2.28 | 2.2 | 2.03 | 2.17 ± 0.10 |
|  | ICR  (g carb/unit insulin) | 37.50 | 37.50 | 37.50 | 37.50 |

The ISF was measured by manually injecting an amount of insulin to pigs after fasting overnight, and it was equal to the blood glucose change in 90 min divided by the amount of insulin. This ICR was measured by manually injecting an amount of insulin to pigs before a meal (~300 g carbohydrates), and pigs’ blood glucose change within two hours was measured. If the change range was within 3.3 mM, it was considered that the amount of insulin was appropriate. The ICR was equal to the weight of carbohydrates divided by the amount of insulin.

**Supplementary Table 2. Comparison of this work and other commercial closed-loop diabetes systems.**

| **Brand** | **System price** | **Dimension of the system (Length × Width × Height, cm^3^)** | **References** |
| --- | --- | --- | --- |
| Medtronic Minimed 670G | ~8000 USD | 9.68 × 5.36 × 2.49 (Pump) + 3.58 × 2.87 × 0.96 (Sensor) = 139.06 cm^3^ | [2] |
| Medtronic Minimed 770G | ~8400 USD | 9.60 × 5.36 × 2.44 (Pump) + 3.58 × 2.87 × 0.96 (Sensor) = 135.42 cm^3^ | [2c, 3] |
| Medtronic Minimed 780G | ~6800  USD | 9.68 × 5.36 × 2.49 (Pump) + 3.58 × 2.87 × 0.96 (Sensor) = 139.06 cm^3^ | [2c, 4] |
| Tandem Diabetes Care Control-IQ (including t:slim X2 pump and Dexcom G6 CGM) | ~4000 USD | 7.95 × 5.08 × 1.52 (Pump) + 4.57 × 3.05 × 1.52 cm (Sensor) = 82.57 cm^3^ | [5] |
| Insulet Omnipod 5 (including Omnipod insulin pump and Dexcom G6 CGM) | ~700 USD | 5.45 × 3.9 × 1.2 (Pump) + 4.57 × 3.05 × 1.52 (Sensor) =46.39 cm^3^ | [5b, 5d, 6] |
| Mylife camAPS FX  (including mylife Ypsopump and Dexcom G6 CGM) | ~6500 USD | 7.8 × 4.6 × 1.6 (Pump) + 4.57 × 3.05 × 1.52 (Sensor) = 78.59 cm^3^ | [5b, 5d, 7] |
| Xindana insulin pump with CGM produced by Sibionics | ~5500 USD | 8.8 × 5.8 × 2.0 (Pump) + 3.35 × 2.0 × 0.53 (Sensor) = 105.63 cm^3^ | [8] |
| Sooil Dana IIS insulin pump with CGM produced by Sibionics | ~7000 USD | 9.1 × 4.55 × 2.0 (Pump) + 3.35 × 2.0 × 0.53 (Sensor) = 86.36 cm^3^ | [8b, 9] |
| MADDOX insulin pump with CGM produced by Sibionics | ~4200 USD | 9.2 × 5.8 × 2.0 (Pump) + 3.35 × 2.0 × 0.53 (Sensor) = 110.27cm^3^ | [8b, 10] |
| IPELE insulin pump with CGM produced by Sibionics | ~3200 USD | 8.8 × 5.8 × 2.0 (Pump) + 3.35 × 2.0 × 0.53 (Sensor) = 105.63 cm^3^ | [8b, 11] |
| 3D printed microtube, dissolvable microneedle array with modified electroosmotic pump | ~10 USD | ~1.5 × 1.5 × 1 = 2.25 cm^3^ | This work |

**Supplementary Table 3. Comparison of this work and other closed-loop diabetes systems in academic publications.**

| **Device compositions** | **Dimension of the system (Length × Width × Height, cm^3^)** | **Type of study animal and in vivo study time** | **References** |
| --- | --- | --- | --- |
| Silicon-nanowire field-effect transistors microneedle array with a 3D printed syringe | ~0.3 × 0.3 × 0.1 = 0.009 cm^3^ | Diabetic rat for 1 h | [12] |
| Wearable patch based on a sweat biosensor and polymeric thermoresponsive metformin loaded microneedles | ~3 × 8 × 0.1 = 2.4 cm^3^ | Diabetic mouse for 6 h | [13] |
| Disposable sweat biosensor and polymeric thermoresponsive metformin loaded microneedles | ~2 × 4 × 0.2 = 1.6 cm^3^ | Diabetic mouse for 6 h | [14] |
| Mesoporous microneedle array based on iontophoresis | ~1.2 × 1.2 × 1 = 1.44 cm^3^ | Diabetic rat for 6 h | [15] |
| Microneedle array with electroosmotic pump | ~2.5 × 2.5 × 1.5 = 9.38 cm^3^ | Diabetic rat for 5 h | [16] |
| Screen-printed microneedle array with electroosmotic pump | ~2.5 × 2.5 × 1 = 6.25 cm^3^ | Diabetic rat for 5 h | [17] |
| Microtube with electroosmotic pump | ~3 × 3 × 2.5 = 22.5 cm^3^ | Diabetic rat for 4 h | [18] |
| Microneedle array with ultrasound-based pump | ~2.5 × 2.5 × 1 = 6.25 cm^3^ | Diabetic rat for 5 h | [19] |
| Microneedle array with electrochemical pump | ~2.5 × 2.5 × 1 = 6.25 cm^3^ | Diabetic rat for 4 h | [20] |
| Microneedle puncher @ hybrid electrodes sensor, microneedle puncher @ delivery patch integrated with peristaltic micropump | 9 × 6 × 3 = 162 cm^3^ | Diabetic rat for 40 h | [21] |
| Glucose-responsive insulin microneedle array | ~2 × 2 × 0.2 = 0.8 cm^3^ | Diabetic mouse for 12 h and diabetic pig for 24 h | [22] |
| Glucose-responsive closed-loop insulin and glucagon microneedle array | ~2 × 2 × 0.2 = 0.8 cm^3^ | Diabetic mouse for 12 h and diabetic pig for 30 h | [23] |
| Microtube with electroosmotic pump | ~2.5 × 2.5 × 1 = 6.25 cm^3^ | Diabetic rat for 4 h | [24] |
| 3D printed microtube, dissolvable microneedle array with modified electroosmotic pump | ~1.5 × 1.5 × 1 = 2.25 cm^3^ | Diabetic pig for 72 h | This work |

References

[1] a) Dexcom, Dexcom Annual Reports, <https://investors.dexcom.com/financials/quarterly-results/default.aspx>, accessed: 2024-04-13, **2024**; b) Medtronic, Annual Meeting Reports, <https://investorrelations.medtronic.com/annual-meeting-reports>, accessed: 2024-03-12, **2024**; c) T. D. Care, Announces Fourth Quarter Full Year 2023, <https://investor.tandemdiabetes.com/news-releases/news-release-details/tandem-diabetes-care-announces-fourth-quarter-and-full-year-2023>, accessed: 2024-03-12.

[2] a) T. Knebel, J. J. Neumiller, *Clinical. Diabetes* **2019**, *37* (1), 94 ; b) Medtronic, 670G User Guide, <https://www.medtronic.com/content/dam/medtronic-com/ca-en/patients/education/diabetes/670G/MiniMed_670G_User_Guide.pdf>, accessed: 2023-10-28, **2023**; c) A. D. Association, Guardian Sensor 3 with Guardian Link 3 transmitter, <https://consumerguide.diabetes.org/products/guardian-sensor-3>, accessed: 2023-10-28, **2023**.

[3] a) A. Diabetes, Medtronic Minimed 770G Insulin Pump, <https://www.adwdiabetes.com/product/21448/medtronic-minimed-770g-pump>, accessed: 2023-10-28, **2023**; b) Medtronic, 770G User Guide, <https://www.medtronicdiabetes.com/sites/default/files/library/download-library/user-guides/MiniMed_770G_System_User_Guide.pdf>, accessed: 2023-10-28.

[4] a) D. s. Bazaar, Medtronic Minimed 780G System, <https://www.doctorsbazaar.com/app/product/medtronic-minimed-780g-system-/kL86g1n7M>, accessed: 2023-10-28, **2023**; b) Medtronic, 780G User guide, <https://www.medtronicdiabetes.com/sites/default/files/library/download-library/user-guides/MiniMed-780G-system-user-guide-with-Guardian-4-sensor.pdf>, accessed: 2023-10-28, **2023**.

[5] a) Healthline, Tandem t:slim X2 Insulin Pump with Basal-IQ: Hands-On Review, <https://www.healthline.com/health/diabetes/tandem-diabetes-basal-iq-review#bottom-line>, accessed: 2023-10-28, **2023**; b) GoodRX, Dexcom G6 Used for Diabetes Type 2 and Diabetes Type 1, <https://www.goodrx.com/dexcom-g6>, accessed: 2023-10-28, **2023**; c) A. D. Association, t:slim X2 Insulin Pump With Control-IQ, <https://consumerguide.diabetes.org/products/t-slim-x2-insulin-pump-with-control-iq-technology>, accessed: 2023-10-28, **2023**; d) Dexcom G6 CGM System, <https://consumerguide.diabetes.org/products/g6-cgm-system>, accessed: 2023-10-28, **2023**.

[6] a) GoodRX, Omnipod 5 Used for Diabetes Type 1, <https://www.goodrx.com/omnipod-5>, accessed: 2023-10-28, **2023**; b) Insulet, Omipod 5 User Guide, <https://www.omnipod.com/sites/default/files/Omnipod-5_User-guide.pdf>, accessed: 2023-10-28, **2023**.

[7] a) D. Advocacy, YpsoPump® receives Health Canada Approval, <https://www.diabetesadvocacy.com/ypsopump-insulin-pump/>, accessed: 2023-10-28, **2023**; b) Ysomed, Ypsomed partners with CamDiab Ltd to drive on smartphone based adaptive automated insulin delivery, <https://www.ypsomed.com/en-AU/news/news-reader-detail-page/ypsomed-partners-with-camdiab-ltd-to-drive-on-smartphone-based-adaptive-automated-insulin-delivery-aid.html>, accessed: 2023-10-28, **2023**.

[8] a) JD, Xindana insulin pump, <https://item.jd.com/10048956926604.html#crumb-wrap>, accessed: 2023-10-28, **2023**; b) Sibionics CGM, <https://item.jd.com/100023189449.html>, accessed: 2023-10-28, **2023**.

[9] Dana RS Insulin Pump, <https://item.jd.com/67970085334.html#crumb-wrap>, accessed: 2023-11-10, **2023**.

[10] MADDOX Insulin Pump, <https://item.jd.com/57744366213.html#crumb-wrap>, accessed: 2023-10-28, **2023**.

[11] IPELE Insulin Pump, <https://item.jd.com/10025072136633.html#crumb-wrap>, accessed: 2023-10-28, **2023**.

[12] O. Heifler, E. Borberg, N. Harpak, M. Zverzhinetsky, V. Krivitsky, I. Gabriel, V. Fourman, D. Sherman, F. Patolsky, *ACS Nano* **2021**, *15* (7), 12019

[13] H. Lee, T. K. Choi, Y. B. Lee, H. R. Cho, R. Ghaffari, L. Wang, H. J. Choi, T. D. Chung, N. S. Lu, T. Hyeon, S. H. Choi, D. H. Kim, *Nat. Nanotechnol.* **2016**, *11* (6), 566

[14] H. Lee, C. Song, Y. S. Hong, M. S. Kim, H. R. Cho, T. Kang, K. Shin, S. H. Choi, T. Hyeon, D. H. Kim, *Sci. Adv.* **2017**, *3* (3), e1601314

[15] X. L. Li, X. S. Huang, J. S. Mo, H. Wang, Q. Q. Huang, C. Yang, T. Zhang, H. J. Chen, T. Hang, F. M. Liu, L. L. Jiang, Q. N. Wu, H. B. Li, N. Hu, X. Xie, *Adv. Sci.* **2021**, *8* (16), 2100827

[16] X. J. Luo, Q. Yu, Y. Q. Liu, W. X. Gai, L. Ye, L. Yang, Y. Cui, *ACS Sens.* **2022**, *7* (5), 1347

[17] L. Y. Yiqun Liu, Yue Cui, *Microsyst. Nanoeng.* **2024**,

[18] Y. Q. Liu, Q. Yu, X. J. Luo, L. Ye, L. Yang, Y. Cui, *Research* **2022**, *2022*, 9870637

[19] X. J. Luo, Q. Yu, L. Yang, Y. Cui, *ACS Sens.* **2023**, *8* (4), 1710

[20] Y. Q. Liu, Q. Yu, L. Ye, L. Yang, Y. Cui, *Lab Chip* **2023**, *23* (3), 421

[21] J. B. Yang, S. T. Zheng, D. Y. Ma, T. Zhang, X. S. Huang, S. Huang, H. J. Chen, J. Wang, L. L. Jiang, X. Xie, *Sci. Adv.* **2022**, *8* (50),

[22] J. C. Yu, J. Q. Wang, Y. Q. Zhang, G. J. Chen, W. W. Mao, Y. Q. Ye, A. R. Kahkoska, J. B. Buse, R. Langer, Z. Gu, *Nat. Biomed. Eng.* **2020**, *4* (5), 499

[23] C. W. Yang, T. Sheng, W. H. Hou, J. Zhang, L. Cheng, H. Wang, W. Liu, S. Q. Wang, X. M. Yu, Y. Q. Zhang, J. C. Yu, Z. Gu, *Sci. Adv.* **2022**, *8* (48), eadd3197

[24] Y. Liu, L. Yang, Y. Cui, *Microsyst. Nanoeng.* **2024**, *10* (1), 112
